# Incidence of acute complications of herpes zoster among immunocompetent adults in England: a matched cohort study using routine health data*

**DOI:** 10.1111/bjd.19687

**Published:** 2021-01-04

**Authors:** H.J. Forbes, K. Bhaskaran, D. Grint, V.H. Hu, S.M. Langan, H.I. McDonald, C. Morton, L. Smeeth, J.L. Walker, C. Warren‐Gash

**Affiliations:** ^1^ London School of Hygiene & Tropical Medicine London UK; ^2^ NIHR Health Protection Research Unit in Immunisation London UK; ^3^ Statistics, Modelling and Economics Department Public Health England London UK

## Abstract

**Background:**

Herpes zoster can cause rare but serious complications; the frequency of these complications has not been well described.

**Objectives:**

To quantify the risks of acute non‐postherpetic neuralgia (PHN) zoster complications, to inform vaccination policy.

**Methods:**

We conducted a cohort study among unvaccinated immunocompetent adults with incident zoster, and age‐, sex‐ and practice‐matched control adults without zoster, using routinely collected health data from the UK Clinical Practice Research Datalink (years 2001 to 2018). Crude attributable risks of complications were estimated as the difference between Kaplan–Meier‐estimated 3‐month cumulative incidences in patients with zoster vs. controls. We used Cox models to obtain hazard ratios for our primary outcomes in patients with and without zoster. Primary outcomes were ocular, neurological, cutaneous, visceral and zoster‐specific complications. We also assessed whether antivirals during acute zoster protected against the complications.

**Results:**

In total 178 964 incident cases of zoster and 1 799 380 controls were included. The absolute risks of zoster‐specific complications within 3 months of zoster diagnosis were 0·37% [95% confidence interval (CI) 0·34–0·39] for Ramsay Hunt syndrome, 0·01% (95% CI 0·0–0·01) for disseminated zoster, 0·04% (95% CI 0·03–0·05) for zoster death and 0·97% (95% CI 0·92–1·00) for zoster hospitalization. For other complications, attributable risks were 0·48% (95% CI 0·44–0·51) for neurological complications, 1·33% (95% CI 1·28–1·39) for ocular complications, 0·29% (95% CI 0·26–0·32) for cutaneous complications and 0·78% (95% CI 0·73–0·84) for visceral complications. Attributable risks were higher among patients > 50 years old. Patients with zoster had raised risks of all primary outcomes relative to controls. Antiviral prescription was associated with reduced risk of neurological complications (hazard ratio 0·61, 95% CI 0·53–0·70).

**Conclusions:**

Non‐PHN complications of zoster were relatively common, which may affect cost‐effectiveness calculations for zoster vaccination. Clinicians should be aware that zoster can lead to various complications, besides PHN.

## Abstract 2

**What is already known about this topic?**

- Herpes zoster is typically mild and self‐limiting; however, it can cause serious cutaneous, visceral, neurological and ocular complications.
- Some complications lead to severe illness, high healthcare costs and mortality, while others can seriously affect quality of life.
- Better knowledge of the frequency of, and risk factors for, such zoster complications would be helpful to inform zoster vaccination policy.

**What does this study add?**

- In this large cohort study among UK adults with zoster, we found raised risks of neurological, ocular, cutaneous, visceral and zoster‐specific complications in the 3 months after zoster diagnosis.
- Estimated absolute risks attributable to zoster were 0·48% [95% confidence interval (CI) 0·44–0·51] for neurological complications, 1·33% (95% CI 1·28–1·39) for ocular complications, 0·29% (95% CI 0·26–0·32) for cutaneous complications and 0·78% (95% CI 0·73–0·84) for visceral complications.

**Linked Comment:** Kridin and Cohen. *Br J Dermatol* 2021; **184**:994–995.

Herpes zoster arises from the reactivation of latent varicella zoster virus following reduced cell‐mediated immunity.^1^ Zoster is typically mild and self‐limiting, but complications can occur. The most common complication is postherpetic neuralgia (PHN), pain at the site of the original zoster rash, which persists beyond rash healing. Risk factors for zoster and PHN include older age and reduced cell‐mediated immunity.^1^, ^2^, ^3^ As well as PHN, serious cutaneous, visceral, neurological and ocular complications can occur.^4^ Some lead to severe illness, high healthcare costs and mortality,^5^ while others can seriously affect quality of life.

Despite their potential seriousness, our knowledge of the frequency of, and risk factors for, such zoster complications is limited. Previous studies have typically been small and restricted to hospital‐diagnosed cases of zoster within limited age groups.^6^, ^7^, ^8^, ^9^, ^10^ Larger cohort studies have not included a comparison group without zoster or have not examined risk factors for the complications.^11^, ^12^

Accurate quantification of the burden of rare but severe complications and their risk factors, and the effectiveness of antiviral medications could help inform zoster vaccine policy decisions and clinical decisions regarding antiviral therapy. We therefore aimed to quantify the relative and absolute risks of acute complications of zoster within 3 months of diagnosis and to assess whether these risks varied by age, among unvaccinated immunocompetent adults. We also sought to assess whether antiviral drugs prescribed during acute zoster modify the risks of these complications.

## Patients and methods

### Study design and setting

We conducted a matched cohort study using routinely collected, anonymized, primary care data from the UK Clinical Practice Research Datalink (CPRD), a not‐for‐profit research service. CPRD hosts two separate databases, GOLD^13^ and Aurum,^14^ which hold data from practices using two different IT software systems: Vision, and Egton Medical Information Systems (EMIS), respectively. The databases are representative of the UK population^13^ and together encompass over 35 million patients lives. These databases include diagnoses (coded using Read codes in GOLD; and Read, Systematized Nomenclature of Medicine and local EMIS codes in Aurum), prescriptions (coded using British National Formulary codes in GOLD and the Dictionary of Medicines and Devices in Aurum), referrals and some basic demographic information. Approximately 80% of CPRD GOLD practices within England, and all CPRD Aurum practices, have linked data from other sources. CPRD data are supplemented by linked data from Hospital Episodes Statistics (HES), Office of National Statistics (ONS) death data and Index of Multiple Deprivation (IMD) data. HES data contain all National Health Service hospital admissions in England since 1997, including diagnoses [coded using International Classification of Diseases, 10th Revision (ICD‐10)] and procedures codes (Office of Population Censuses and Surveys Classification of Interventions and Procedures version 4). IMD data provide quintiles of deprivation based on the patient or practice postcode. ONS data contain date, place and causes of death (coded using ICD‐10).

### Participants

Eligible patients were those in CPRD (GOLD or Aurum databases) with linked HES, ONS and IMD data. Patients had at least 12 months of follow‐up after the start of their research‐standard CPRD follow‐up (that is, current registration date in CPRD Aurum and latest of current registration date and practice up to standard date in CPRD GOLD).^15^ Patients with zoster were those with a first‐ever record of acute zoster (including no history of PHN), aged ≥ 18 years at zoster diagnosis (as zoster is very rare among children) and diagnosed between 1 January 2001 and 31 July 2018.

Each patient with zoster was randomly matched to 10 patients without zoster (on age within 1 year, practice and sex) to ensure sufficiently precise estimates could be achieved. People not exposed to zoster had to be registered in a practice at the index date of the case (the incident zoster diagnosis date), to have been registered for at least 12 months before and to have no history of zoster at cohort entry. The unexposed cohort was assigned an index date identical to that of their case. Exposure status was time‐varying, so unexposed patients who were matched to an exposed patient with zoster could switch to the exposed category if they subsequently developed zoster. In this situation, the patient was censored as a control at the date of zoster diagnosis, and contributed exposed time from their zoster index date, with their own set of matched patients without zoster. Patients with a history of moderate or severe immunosuppression at the index date, defined as a weakened immune system as the result of conditions or medication, were excluded from the study^16^ (Appendix S1; see Supporting Information).

### Variables

The primary exposure, zoster, was identified in CPRD data and in HES data (all codes listed in Appendix S1; see Supporting Information). If zoster was identified in both HES and CPRD, the earliest recorded zoster diagnosis was used. Hospitalizations were considered related to zoster if the zoster diagnosis was recorded in the primary diagnosis field of any hospitalization. A secondary exposure, primary care prescription of antivirals within 7 days of zoster diagnosis (given for the treatment of acute zoster), was identified in CPRD.

#### Outcomes

We searched for complications in CPRD GOLD and Aurum, as well as linked HES data (first diagnostic field of any episode). Detailed definitions of each outcome are provided in Appendix S1 (see Supporting Information). There were five primary outcome groups (Table 1): (i) acute neurological complications (not including PHN or Ramsay Hunt syndrome); (ii) cutaneous complications (not including disseminated zoster); (iii) visceral or systemic complications; (iv) ocular complications (not including cranial nerve palsies as these were included within neurological complications) and (v) zoster‐specific outcomes. Secondary outcomes were specific types of complications, which collectively comprise the primary outcomes (Table 1). We searched for these outcomes from the day of zoster diagnosis up to 1 year after zoster. Complications were selected based on the existing literature and biological plausibility.

**Table 1:** Zoster complications: the five primary outcomes and the specific conditions comprising each outcome

Neurological: cranial and peripheral nerve palsies (excluding Ramsay Hunt syndrome), encephalitis, transverse myelitis, meningitis, Guillain–Barré syndrome and stroke
Ocular: keratitis, conjunctivitis, iritis, blepharitis, retinitis, optic neuritis, orbital myositis, scleritis, glaucoma, vision loss or blindness, nonspecific eye infections
Cutaneous: secondary bacterial skin infection (cellulitis, necrotizing fasciitis and erysipelas)
Visceral and systemic: pneumonia, hepatitis, pancreatitis, sepsis, pulmonary embolism, osteomyelitis, pleuritis, peritonitis, myocardial infarction, myositis, myocarditis, pericarditis and endocarditis
Zoster‐specific complications: Ramsay Hunt syndrome, varicella zoster virus dissemination, zoster‐related mortality (identified from Office for National Statistics data, defined as any ICD‐9 or ICD‐10 code related to zoster recorded as a cause of death), and zoster‐related hospitalization (defined as a zoster code in the primary field of the first episode)

ICD, International Classification of Diseases.

#### Covariates

Comorbidities included all long‐term health conditions that have been associated with increased risk of zoster and/or PHN, including liver disease, asthma, chronic kidney disease, chronic obstructive pulmonary disease, depression, diabetes and autoimmune disorders (rheumatoid arthritis, systemic lupus erythematosus or inflammatory bowel disease).

Other covariates included age (< 50 and ≥ 50 years), sex (male and female), ethnicity (white and not white, from CPRD and HES data), other health and lifestyle factors (body mass index and smoking status) and finally socioeconomic status (quintiles of the 2015 IMD, using patient IMD and, if missing, practice IMD).

### Statistical analysis

For the main analyses, patient‐level data from CPRD GOLD and Aurum were pooled. The cohort study was restricted to zoster‐unvaccinated person‐time. Follow‐up started at the index date (date of zoster diagnosis for the exposed patient within each matched set) and ended at the earliest of death date (using ONS date or, if missing, CPRD death date), transfer out of practice, last data collection date, 31 July 2018, the patient developing an outcome of interest, zoster vaccination date or 3 months following the index date. Patients contributing at least 1 day of follow‐up were included in the study; a day was added for patients entering and exiting the study on the same day, to include patients who present initially with a zoster‐related complication such as ‘zoster encephalitis’.

We used a Kaplan–Meier approach to calculate the cumulative unadjusted incidence of each primary outcome at 3 months, in the exposed and unexposed groups; the difference in cumulative incidence was the attributable risk.

We used survival analysis to assess the overall association between zoster and each primary and secondary outcome in the 3 months following zoster. We fitted Cox models stratified by matched set, with current age as the underlying timescale, to obtain hazard ratios (HRs) for our primary outcomes in patients with and without zoster. Our models adjusted initially for age and sex, then additional covariates. Relative effect measures were not calculated for the zoster‐specific complications. We went on to stratify our results by patient‐specific characteristics by fitting separate models for patients in each age group and with long‐term health conditions, and tested for interactions using the likelihood ratio test.

Finally, among only patients with zoster, we assessed the incidence and risk of the outcomes according to whether patients were given antivirals within 7 days of diagnosis. We excluded patients whose zoster was diagnosed in HES or had an inpatient HES visit for zoster within 7 days of diagnosis, as secondary care antiviral prescriptions may not be recorded in CPRD.

#### Prespecified sensitivity analysis

We repeated the analysis of the association between zoster and each primary outcome by study database, and combined the effect estimates using random effect meta‐analysis (i.e. two‐stage individual patient data analysis). We also analysed this risk of complications in the 0–6 months and 0–12 months following zoster, in order to confirm the risk of complications was reduced.

### Ethical approval

This study was approved by the Independent Scientific Advisory committee (no. 18_278) and London School of Hygiene & Tropical Medicine ethics (16227).

## Results

The cohort included 178 964 patients with zoster (115 790 from GOLD and 63 174 from Aurum) and 1 799 380 sex‐, age‐ and practice‐matched unexposed patients. Almost 60% of the final cohort were female and the median age at cohort entry was 62 years (Table 2). Patients with zoster had a higher prevalence of medical conditions including depression (3·1% vs. 2·7%), chronic obstructive pulmonary disease (4·9% vs. 3·6%) and chronic kidney disease (7·7% vs. 6·0%) (Table 2). Characteristics of the patients in GOLD and Aurum were similar in terms of age, socioeconomic status and comorbidities (Table S1; see Supporting Information). The mean follow‐up time was 88 days (SD 9 days) in the exposed and in the unexposed.

**Table 2:** Baseline characteristics of patients with zoster and matched patients without zoster in Clinical Practice Research Datalink (*n* = 1 978 344) and by individual database

	With zoster	Without zoster
Number of patients	178 964 (100)	1 799 380 (100)
Database		
GOLD	115 790 (64·7)	1 169 272 (65·0)
Aurum	63 174 (35·3)	630 108 (35·0)
Sex female	105 288 (58·8)	1 050 183 (58·4)
Age at cohort entry (years), median (IQR)	62·1 (48·2–74·1)	61·5 (47·8–73·0)
Ethnicity		
White	120 898 (67·6)	1 134 593 (63·1)
Not white	6972 (3·9)	88 200 (4·9)
Missing	51 094 (28·5)	576 587 (32·0)
Socioeconomic status^a^		
1 (least deprived)	46 031 (25·7)	457 150 (25·4)
2	42 099 (23·5)	420 621 (23·4)
3	36 479 (20·4)	367 608 (20·4)
4	30 979 (17·3)	312 014 (17·3)
5 (most deprived)	23 376 (13·1)	241 987 (13·4)
Body mass index category		
Underweight	3621 (2·0)	35 887 (2·0)
Normal weight	63 049 (35·2)	615 593 (34·2)
Overweight	59 671 (33·3)	576 081 (32·0)
Obese	38 539 (21·5)	370 742 (20·6)
Missing	14 084 (7·9)	201 077 (11·2)
Smoking status		
Never smoked	64 372 (36·0)	669 354 (37·2)
Currently smokes	36 605 (20·5)	394 526 (21·9)
Formerly smoked	76 258 (42·6)	685 093 (38·1)
Missing	1729 (1·0)	50 407 (2·8)
Comorbidity		
Liver disease	543 (0·3)	5461 (0·3)
Asthma	29 302 (16·4)	237 518 (13·2)
Chronic kidney disease	13 829 (7·7)	108 374 (6·0)
Chronic obstructive pulmonary disease	8747 (4·9)	65 245 (3·6)
Depression	5504 (3·1)	49 097 (2·7)
Diabetes	16 306 (9·1)	146 446 (8·1)
Autoimmune disorders^b^	4777 (2·7)	34 800 (1·9)

The data are presented as the number (%) unless otherwise stated. IQR, interquartile range. ^a^Measured by Index of Multiple Deprivation score: patient level, or practice level if patient level unavailable (GOLD, *n* = 1425). ^b^Rheumatoid arthritis, systemic lupus erythematosus or inflammatory bowel disease.

Table 3 shows the percentage of patients with zoster with zoster‐attributable complications in the 3 months following diagnosis, overall and by risk group. In total, 0·48% [95% confidence interval (CI) 0·44–0·51] of patients with zoster developed neurological complications, 1·33% (95% CI 1·28–1·39) ocular complications, 0·29% (95% CI 0·26–0·32) cutaneous complications and 0·78% (95% CI 0·73–0·84) visceral complications, attributable to zoster. Regarding the zoster‐specific complications, 0·37% of patients with zoster developed Ramsay Hunt syndrome, 0·01% developed disseminated zoster, 0·04% died of zoster and 0·97% were hospitalized. A higher proportion of older patients with zoster (≥ 50 years) developed each primary outcome, compared with younger patients (< 50 years), aside from Ramsay Hunt syndrome.

**Table 3:** Overall and age‐specific risk^a^ of complications (percentage with 95% confidence interval) attributable to zoster, among immunocompetent patients in the 3 months following zoster

	All adults	Age at zoster diagnosis	
		< 50 years	≥ 50 years
All neurological^b^	0·48 (0·44–0·51)	0·44 (0·39–0·50)	0·49 (0·45–0·53)
All ocular	1·33 (1·28–1·39)	0·85 (0·78–0·94)	1·50 (1·44–1·57)
All cutaneous^c^	0·29 (0·26–0·32)	0·20 (0·16–0·24)	0·32 (0·28–0·35)
All visceral	0·78 (0·73–0·84)	0·50 (0·44–0·59)	0·89 (0·82–0·96)
Zoster‐specific complications			
Ramsay Hunt syndrome	0·37 (0·34–0·39)	0·43 (0·37–0·49)	0·34 (0·31–0·37)
Disseminated zoster	0·01 (0·00–0·01)	0	0·01 (0·00–0·01)
Zoster death	0·04 (0·03–0·05)	0	0·06 (0·05–0·07)
Zoster hospitalization	0·97 (0·92–1·00)	0·35 (0·30–0·40)	1·19 (1·13–1·25)

^a^Total percentages were calculated using a Kaplan–Meier approach to determine the cumulative incidence of each primary outcome at 3 months, in the exposed and unexposed groups; the difference in cumulative incidence was the attributable risk. ^b^Not including Ramsay Hunt syndrome or postherpetic neuralgia. ^c^Not including disseminated zoster.

Table 4 shows the burden and association with zoster for each primary outcome among all adults in CPRD GOLD or Aurum, overall and by age group. The relative risk of each primary outcome was elevated among all patients with zoster, compared with patients without zoster. The overall relative risk was largest for neurological complications (HR 3·63, 95% CI 3·35–3·94), then ocular (HR 1·99, 95% CI 1·92–2·07), cutaneous (HR 1·64, 95% CI 1·52–1·77) and visceral complications (HR 1·30, 95% CI 1·25–1·35). There was strong evidence that the risks varied by age at zoster diagnosis (*P*‐value for interaction < 0·001); the magnitude of the relative risks were greater in younger patients (< 50 years) than in older patients. Table S2 (see Supporting Information) shows the burden and association between zoster and individual conditions making up the composite primary outcomes. Encephalitis, meningitis and nerve palsies had the strongest associations of the neurological conditions (all HRs > 8), and orbital myositis, iritis and keratitis had strongest associations of the ocular complications (all HRs > 8).

**Table 4:** Burden and association between zoster and each primary outcome among all immunocompetent adults in Clinical Practice Research Datalink GOLD or Aurum, overall and by age at zoster diagnosis, in the 3 months following zoster

	Zoster, *n* (%)	No zoster, *n* (%)	Age‐ and sex‐adjusted HR (95% CI)^a^	Fully adjusted HR (95% CI)^b^	*P*‐value^c^
All neurological^d^	1160 (0·6)	3127 (0·2)	3·78 (3·51–4·08)	3·63 (3·35–3·94)	–
All ocular	4662 (2·6)	23 079 (1·3)	2·08 (2·01–2·15)	1·99 (1·92–2·07)	
All cutaneous^e^	1231 (0·7)	7207 (0·4)	1·66 (1·55–1·77)	1·64 (1·52–1·77)	
All visceral	4719 (2·6)	33 430 (1·9)	1·41 (1·37–1·46)	1·30 (1·25–1·35)	
By age (years)					
All neurological^d^					
< 50	253 (0·5)	361 (0·07)	7·13 (6·07–8·39)	6·58 (5·51–7·85)	< 0·001
≥ 50	907 (0·7)	2766 (0·2)	3·22 (2·96–3·51)	3·13 (2·86–3·44)	
All ocular					
< 50	650 (1·3)	2364 (0·5)	2·81 (2·58–3·07)	2·57 (2·34–2·83)	< 0·001
≥ 50	4012 (3·1)	20 715 (1·6)	1·97 (1·90–2·05)	1·91 (1·84–1·99)	
All cutaneous^e^					
< 50	163 (0·3)	651 (0·1)	2·54 (2·14–3·02)	2·44 (2·03–2·95)	< 0·001
≥ 50	1068 (0·8)	6556 (0·5)	1·55 (1·43–1·67)	1·55 (1·43–1·68)	
All visceral					
< 50	635 (1·3)	3913 (0·8)	1·66 (1·53–1·81)	1·53 (1·39–1·67)	< 0·001
≥ 50	4084 (3·1)	29 517 (2·3)	1·37 (1·32–1·43)	1·27 (1·22–1·32)	

HR, hazard ratio; CI, confidence interval. ^a^Cox model with age as the underlying timescale. ^b^Additionally adjusted for rheumatoid arthritis, inflammatory bowel disease, systemic lupus erythematosus, socioeconomic status, smoking status, body mass index, depression, asthma, chronic obstructive pulmonary disorder, chronic kidney disease, liver disease and diabetes. ^c^ *P*‐value for interaction. ^d^Not including Ramsay Hunt syndrome or postherpetic neuralgia. ^e^Not including disseminated zoster.

Table 5 shows the association between antiviral use and each primary outcome among cases of zoster; 3164 (1·8%) patients were dropped from analysis as they might have received antivirals in hospital. There was good evidence that patients prescribed antivirals in primary care were at reduced risk of certain complications, specifically Ramsay Hunt syndrome (adjusted HR 0·52, 95% CI 0·45–0·62), zoster hospitalization (adjusted HR 0·62, 95% CI 0·50–0·78) and neurological complications (adjusted HR 0·61, 95% CI 0·53–0·70), compared with patients with zoster not prescribed antivirals.

**Table 5:** Association between non‐postherpetic neuralgia complications within 3 months of zoster diagnosis and antiviral use, among immunocompetent cases of zoster unlikely to receive antivirals in hospital (*n* = 175 800)

	Given antivirals, *n* (%)	Not given antivirals, *n* (%)	Fully adjusted HR (95% CI)^a^
Zoster‐specific complications			
Ramsay Hunt syndrome	281 (0·3)	359 (0·5)	0·52 (0·45–0·62)
Disseminated zoster	1 (0·001)	2 (0·003)	0·32 (0·03–3·62)
Zoster death	22 (0·02)	10 (0·01)	2·44 (0·81–7·40)
Zoster hospitalization	189 (0·2)	180 (0·3)	0·62 (0·50–0·78)
All neurological^b^	474 (0·4)	496 (0·7)	0·61 (0·53–0·70)
All ocular	2762 (2·6)	1746 (2·5)	0·98 (0·92–1·04)
All cutaneous^c^	658 (0·6)	452 (0·6)	0·88 (0·78–1·00)
All visceral	2627 (2·5)	1650 (2·4)	0·99 (0·92–1·05)

HR, hazard ratio; CI, confidence interval. ^a^Cox model with age as the underlying timescale adjusted for sex, rheumatoid arthritis, inflammatory bowel disease, systemic lupus erythematosus, socioeconomic status, smoking status, body mass index, depression, asthma, chronic obstructive pulmonary disorder, chronic kidney disease, liver disease and diabetes. ^b^Not including Ramsay Hunt syndrome or postherpetic neuralgia. ^c^Not including disseminated zoster.

### Sensitivity analyses

The associations between zoster and each primary outcome were very similar in both CPRD GOLD and Aurum, and when running a two‐stage individual patient data meta‐analysis (Table S3; see Supporting Information). The magnitude of association between zoster and each complication was reduced in the 0–6 months and 0–12 months following zoster (Table S4; see Supporting Information).

## Discussion

In our cohort study of 178 964 UK adults with zoster, from CPRD GOLD and Aurum, we found raised risks of neurological, ocular, cutaneous, visceral and zoster‐specific complications in the 3 months after zoster diagnosis. Estimated absolute risks attributable to zoster were 0·48% (95% CI 0·44–0·51) for neurological complications, 1·33% (95% CI 1·28–1·39) for ocular complications, 0·29% (95% CI 0·26–0·32) for cutaneous complications and 0·78% (95% CI 0·73–0·84) for visceral complications. The estimated absolute attributable risks of zoster‐specific complications were 0·37% for Ramsay Hunt syndrome, 0·01% for disseminated zoster, 0·04% for death due to zoster and 0·97% for zoster‐related hospitalization. Younger patients with zoster (< 50 years) had higher relative risk, but lower absolute risk, of complications compared with older patients (≥ 50 years). Antiviral use during acute zoster appeared to protect against neurological complications, Ramsay Hunt syndrome and hospitalization. Clinicians should be aware that zoster can lead to various complications, besides PHN.

The UK zoster vaccine cost‐effectiveness analysis estimates that among those aged > 65 years with zoster, 2·0% are hospitalized and 0·06% die;^17^ this is in line with our study, which estimates that (among patients with zoster aged ≥ 50 years) 1·3% are hospitalized and 0·06% die. A study in Germany using a health insurance database containing nationwide routine outpatient data found that 4·1% of immunocompetent patients with zoster (age ≥ 50 years) developed zoster encephalitis, zoster meningitis and zoster with other complications.^12^ A US population‐based study using administrative data supplemented by medical review reported that 3·7% of 859 cases of zoster experienced a non‐PHN complication; those of older age and with certain comorbidities (including inflammatory bowel disease, HIV and diabetes) were more likely to experience complications.^18^ A recent study among 600 immunocompetent unvaccinated patients with zoster (age ≥ 50 years), selected from a large healthcare organization in the USA, found that the proportions of cutaneous, neurological and other complications were higher than in our study, at 6·40%, 0·77% and 1·01%, respectively; however, our estimates fall inside their CIs. This latter study assessed for non‐PHN complications by medical chart review, which may have led to a higher proportion of complications than in our study.^19^

We found that patients with zoster aged < 50 years had a greater relative risk of developing complications than patients ≥ 50 years of age. This is likely because the baseline rate of such complications in younger populations is much lower. However, overall the absolute risks of complications were lower in patients under, compared with over, 50 years of age.

This is the first large‐scale UK‐based epidemiological study examining the risk of non‐PHN complications of zoster using routinely collected health data. Collectively, rarer zoster complications together mean that around one in 20 patients with zoster will develop a complication beyond PHN. Highlighting the risk of complications to the general public may help increase the uptake of shingles vaccination, as qualitative research has shown that the perceived severity of a disease influences individuals’ decisions about whether or not to vaccinate; greater perceived harm due to the infectious agent is associated with higher vaccine uptake.^20^

Zoster diagnoses in UK primary and secondary care are based on clinical judgement, without laboratory confirmation, and have not been validated in CPRD or HES. However, a validation study of zoster in a primary care setting in the Netherlands demonstrated a high positive predictive value of zoster diagnosis of 91%, when confirmed by the presence of varicella zoster virus antibodies.^21^

There might be differential misclassification of antiviral receipt with respect to zoster severity, for example if more severe cases of zoster are treated within secondary care. Prescription data in secondary care were not available, and although we removed patients with a zoster‐related hospitalization within 7 days of zoster diagnosis, misclassification of antiviral status may lead to those ‘not given’ antivirals representing more severe cases receiving treatment in hospital. There was strong evidence that patients given antivirals were less likely to have a hospitalization for zoster and Ramsay Hunt syndrome, which may reflect this misclassification.

There may be misclassification of some zoster complications. We included nonspecific zoster complication codes (such as encephalitis), potentially overestimating zoster‐attributable complications. However, by comparing with a cohort without zoster we were able to account for the baseline rate of each complication in the general population. Alternatively, we may have underestimated zoster complications. A US study comparing zoster complication rates among 1959 patients with zoster obtained from administrative data and supplemented by medical record review found that administrative data alone significantly underestimated complication rates (0·6% vs. 6·4%), for all complications besides PHN and ophthalmic zoster.^22^ However, only zoster‐specific codes were used to identify complications (specifically ICD‐9 codes beginning O.53); our study has supplemented specific zoster codes with nonspecific zoster codes, and therefore we have made some progress in improving the estimates of our outcomes.

The Aurum and GOLD databases have some differences, including how diagnoses are coded and when patients’ follow‐up is initiated. However, in sensitivity analyses the findings were very similar in both the CPRD GOLD and Aurum data, suggesting that combining the databases did not bias our main analysis.

There is a possibility of uncontrolled confounding in the comparison of the Kaplan–Meier cumulative incidences, in which it was not possible to adjust for potential confounders. However, matching (on age, sex and practice) should have reduced key differences, and the Cox modelling analysis suggested minimal confounding from other variables, so any residual confounding is likely to be small.

This study indicates that antivirals may reduce the risk of developing neurological complications. This supports existing guidelines to prescribe oral antivirals (aciclovir, valaciclovir or famciclovir) to those over 50 years of age with zoster. We know from previous research that oral antivirals are underprescribed in UK primary care.^23^ Current cost‐effectiveness analyses for zoster vaccination in the UK account for PHN and hospitalization for zoster only. Although individual complications are rare, considered collectively they are not infrequent, which may mean we are underestimating the costs of zoster. We would therefore suggest that these non‐PHN complications could be incorporated into future cost‐effectiveness work on zoster vaccinations. The study also highlights for clinicians that zoster can lead to various complications, besides PHN.

In conclusion, we observed clinically significant raised risks of neurological, ocular, cutaneous, visceral and zoster‐specific complications among patients with zoster, within 3 months of zoster diagnosis. Including non‐PHN complications in cost‐effectiveness models of the zoster vaccine might alter the recommendations, as some complications would have significant cost implications for the healthcare system, as well as important impacts on quality of life. Antiviral use during acute zoster appeared to protect against neurological zoster complications, an observation that may help encourage general practitioners to adhere to antiviral prescribing guidelines during acute zoster.

## Author Contribution

**Harriet Forbes:** Conceptualization (lead); Data curation (lead); Formal analysis (lead); Funding acquisition (lead); Investigation (lead); Methodology (lead); Project administration (lead); Resources (lead); Software (lead); Validation (lead); Visualization (lead); Writing‐original draft (lead); Writing‐review & editing (lead). **Krishnan Bhaskaran:** Methodology (equal); Supervision (equal); Writing‐review & editing (equal). **Daniel Grint:** Writing‐review & editing (equal). **Victor Hu:** Methodology (equal); Writing‐review & editing (equal). **Sinead Langan:** Methodology (equal); Writing‐review & editing (equal). **Helen McDonald:** Methodology (equal); Writing‐review & editing (equal). **Caroline Morton:** Methodology (equal); Writing‐review & editing (equal). **Liam Smeeth:** Methodology (equal); Writing‐review & editing (equal). **Jemma Walker:** Methodology (equal); Writing‐review & editing (equal). **Charlotte Warren‐Gash:** Conceptualization (equal); Methodology (equal); Supervision (lead); Writing‐review & editing (equal).

## Supporting information


**Appendix S1.** Definition of severe immunosuppression, and code lists.
**Table S1.** Baseline characteristics of patients with zoster and matched patients without zoster in Clinical Practice Research Datalink and by individual database.
**Table S2.** Burden and association between zoster and each individual outcome among all immunocompetent adults in Clinical Practice Research Datalink GOLD or Aurum.
**Table S3.** Burden and association between zoster and each primary outcome among all immunocompetent adults in Clinical Practice Research Datalink GOLD or Aurum, in the 3 months following zoster diagnosis.
**Table S4.** Burden and association between zoster and each primary outcome among all immunocompetent adults in Clinical Practice Research Datalink GOLD or Aurum, stratified by time since cohort entry.
