# Supplementary material for: Incidence of acute complications of herpes zoster among immunocompetent adults in England: a matched cohort study using routine health data
Source: Br J Dermatol. 2021 Jan 4;184(6):1077–84. doi: 10.1111/bjd.19687 (PMC8607468; doi:10.1111/bjd.19687)
Supplement: Supplementary file 1 — Appendix S1. Definition of severe immunosuppression, and code lists. Table S1. Baseline characteristics of patients with zoster and matched patients without zoster in Clinical Practice Research Datalink and by individual database. Table S2. Burden and association between zoster and each individual outcome among all immunocompetent adults in Clinical Practice Research Datalink GOLD or Aurum. Table S3. Burden and association between zoster and each primary outcome among all immunocompetent adults in Clinical Practice Research Datalink GOLD or Aurum, in the 3 months following zoster diagnosis. Table S4. Burden and association between zoster and each primary outcome among all immunocompetent adults in Clinical Practice Research Datalink GOLD or Aurum, stratified by time since cohort entry. [file BJD-184-1077-s001.docx]

**Appendix**

Contents

[Additional results 3](#_Toc40277267)

[**Table S1**: Baseline characteristics of zoster and matched non-zoster patients in CPRD, and by individual database. Figures are numbers (percentage) unless otherwise stated. 4](#_Toc40277268)

[**Table S2**: Burden and association between zoster and each individual outcome among all immunocompetent adults in CPRD GOLD or Aurum. 6](#_Toc40277269)

[**Table S3**: Burden and association between zoster and each primary outcome among all immunocompetent adults in CPRD GOLD or Aurum, in the three months following zoster diagnosis. 7](#_Toc40277270)

[**Table S4**: Burden and association between zoster and each primary outcome among all immunocompetent adults in CPRD GOLD or Aurum, stratified by time since cohort entry. 9](#_Toc40277271)

[Definition of severe immunosuppression 11](#_Toc40277272)

[Code lists 12](#_Toc40277273)

[Zoster 13](#_Toc40277274)

[Postherpetic Neuralgia 15](#_Toc40277275)

[Ramsay-Hunt syndrome 16](#_Toc40277276)

[Neurological complications 17](#_Toc40277277)

[Other cranial and peripheral nerve palsies 18](#_Toc40277278)

[Encephalitis 20](#_Toc40277279)

[Transverse myelitis 22](#_Toc40277280)

[Meningitis 23](#_Toc40277281)

[Guillain-Barré syndrome 25](#_Toc40277282)

[Stroke 26](#_Toc40277283)

[Ophthalmic complications 31](#_Toc40277284)

[Blepharitis 32](#_Toc40277285)

[Conjunctivitis 33](#_Toc40277286)

[Retinitis 36](#_Toc40277287)

[Optic neuritis 38](#_Toc40277288)

[Orbital myositis 39](#_Toc40277289)

[Iritis 40](#_Toc40277290)

[Keratitis 42](#_Toc40277291)

[Keratitis – surgical codes 45](#_Toc40277292)

[Episcleritis/ Scleritis 47](#_Toc40277293)

[Glaucoma - diagnoses 48](#_Toc40277294)

[Glaucoma – surgical codes 50](#_Toc40277295)

[Vision loss/Blindness 52](#_Toc40277296)

[Non-specific eye infection 55](#_Toc40277297)

[Eye treatments (antivirals/antibacterials) 56](#_Toc40277298)

[Cutaneous 62](#_Toc40277299)

[Disseminated zoster 63](#_Toc40277300)

[Cellulitis 64](#_Toc40277301)

[Necrotising fasciitis 68](#_Toc40277302)

[Erysipelas 69](#_Toc40277303)

[Visceral 70](#_Toc40277304)

[Septicaemia (bacterial or viral) 71](#_Toc40277305)

[Pulmonary embolism/DVT 75](#_Toc40277306)

[Pneumonia (bacterial or viral) 77](#_Toc40277307)

[Acute hepatitis 83](#_Toc40277308)

[Acute pancreatitis 85](#_Toc40277309)

[Acute osteomyelitis 86](#_Toc40277310)

[Acute pleuritic 91](#_Toc40277311)

[Peritonitis 92](#_Toc40277312)

[Myositis 93](#_Toc40277313)

[Myocarditis 95](#_Toc40277314)

[Pericarditis 96](#_Toc40277315)

[Endocarditis 97](#_Toc40277316)

# Additional results

### **Table S1**: Baseline characteristics of zoster and matched non-zoster patients in CPRD, and by individual database. Figures are numbers (percentage) unless otherwise stated.

|  |  | **CPRD all (N=1,978,344)** | | **GOLD (N=1,285,062)** | | **Aurum (N=693,282)** | |
| --- | --- | --- | --- | --- | --- | --- | --- |
|  |  | **Zoster n (%)** | **Non-zoster n (%)** | **Zoster n (%)** | **Non-zoster n (%)** | **Zoster n (%)** | **Non-zoster n (%)** |
| No of patients |  | 178964 (100) | 1799380 (100) | 115790 (100) | 1169272 (100) | 63174 (100) | 630108 (100) |
| Females |  | 105288 (58.8) | 1050183 (58.4) | 68562 (59.2) | 688096 (58.8) | 36726 (58.1) | 362087 (57.5) |
| Age at cohort entry in years (median, IQR) |  | 62.1 (48.2-74.1) | 61.5 (47.8-73.0) | 62.3 (48.7-74.3) | 62.0 (48.5-73.5) | 61.6 (47.1-73.7) | 60.8 (46.5-72.1) |
| Ethnicity | White | 120898 (67.6) | 1134593 (63.1) | 73693 (63.6) | 695848 (59.5) | 47205 (74.7) | 438745 (69.6) |
|  | Non-white | 6972 ( 3.9) | 88200 ( 4.9) | 3129 ( 2.7) | 41099 ( 3.5) | 3843 ( 6.1) | 47101 ( 7.5) |
|  | Missing | 51094 (28.5) | 576587 (32.0) | 38968 (33.7) | 432325 (37.0) | 12126 (19.2) | 144262 (22.9) |
| Socioeconomic status* | 1 (least deprived) | 46031 (25.7) | 457150 (25.4) | 28976 (25.0) | 288996 (24.7) | 17055 (27.0) | 168154 (26.7) |
|  | 2 | 42099 (23.5) | 420621 (23.4) | 26916 (23.2) | 271138 (23.2) | 15183 (24.0) | 149483 (23.7) |
|  | 3 | 36479 (20.4) | 367608 (20.4) | 24664 (21.3) | 250119 (21.4) | 11815 (18.7) | 117489 (18.6) |
|  | 4 | 30979 (17.3) | 312014 (17.3) | 19922 (17.2) | 200583 (17.2) | 11057 (17.5) | 111431 (17.7) |
|  | 5 (most deprived) | 23376 (13.1) | 241987 (13.4) | 15312 (13.2) | 158436 (13.5) | 8064 (12.8) | 83551 (13.3) |
| Body mass index | Underweight | 3621 ( 2.0) | 35887 ( 2.0) | 2318 ( 2.0) | 22984 ( 2.0) | 1303 ( 2.1) | 12903 ( 2.0) |
|  | Normal Weight | 63049 (35.2) | 615593 (34.2) | 40743 (35.2) | 398764 (34.1) | 22306 (35.3) | 216829 (34.4) |
|  | Overweight | 59671 (33.3) | 576081 (32.0) | 38704 (33.4) | 375872 (32.1) | 20967 (33.2) | 200209 (31.8) |
|  | Obese | 38539 (21.5) | 370742 (20.6) | 24793 (21.4) | 240116 (20.5) | 13746 (21.8) | 130626 (20.7) |
|  | Missing | 14084 ( 7.9) | 201077 (11.2) | 9232 ( 8.0) | 131536 (11.2) | 4852 ( 7.7) | 69541 (11.0) |
| Smoking status | Non-smoker | 64372 (36.0) | 669354 (37.2) | 42788 (37.0) | 443933 (38.0) | 21584 (34.2) | 225421 (35.8) |
|  | Current smoker | 36605 (20.5) | 394526 (21.9) | 24810 (21.4) | 267713 (22.9) | 11795 (18.7) | 126813 (20.1) |
|  | Ex-smoker | 76258 (42.6) | 685093 (38.1) | 47127 (40.7) | 425912 (36.4) | 29131 (46.1) | 259181 (41.1) |
|  | Missing | 1729 ( 1.0) | 50407 ( 2.8) | 1065 ( 0.9) | 31714 ( 2.7) | 664 ( 1.1) | 18693 ( 3.0) |
| Alcoholic liver disease |  | 543 ( 0.3) | 5461 ( 0.3) | 244 ( 0.2) | 2536 ( 0.2) | 299 ( 0.5) | 2925 ( 0.5) |
| Asthma |  | 29302 (16.4) | 237518 (13.2) | 18993 (16.4) | 155608 (13.3) | 10309 (16.3) | 81910 (13.0) |
| Chronic kidney disease |  | 13829 ( 7.7) | 108374 ( 6.0) | 8616 ( 7.4) | 69769 ( 6.0) | 5213 ( 8.3) | 38605 ( 6.1) |
| Chronic obstructive pulmonary disorder |  | 8747 ( 4.9) | 65245 ( 3.6) | 5685 ( 4.9) | 42708 ( 3.7) | 3062 ( 4.8) | 22537 ( 3.6) |
| Depression |  | 5504 ( 3.1) | 49097 ( 2.7) | 3741 ( 3.2) | 33732 ( 2.9) | 1763 ( 2.8) | 15365 ( 2.4) |
| Diabetes |  | 16306 ( 9.1) | 146446 ( 8.1) | 9855 ( 8.5) | 91258 ( 7.8) | 6451 (10.2) | 55188 ( 8.8) |
| Autoimmune disorders** |  | 4777 ( 2.7) | 34800 ( 1.9) | 3374 ( 2.9) | 24249 ( 2.1) | 1403 ( 2.2) | 10551 ( 1.7) |
| *Measured by Index of Multiple deprivation score: patient level, or practice level if patients level unavailable (GOLD, n=1425). **Rheumatoid arthritis, Systemic lupus erythematosus or Inflammatory bowel disease. |  |  |  |  |  |  |  |

**Table S2**: Burden and association between zoster and each individual outcome among all immunocompetent adults in CPRD GOLD or Aurum.

|  | **Zoster, n (%)** | **Non-zoster, n (%)** | **Age and sex-adjusted HR (95% CI)†** | **Fully adjusted HR (95% CI)‡** |
| --- | --- | --- | --- | --- |
|  |  |  |  |  |
| **All neurological complications** | 1160 (0.648) | 3127 (0.174) | 3.78 (3.51-4.08) | 3.63 (3.35-3.94) |
| Nerve palsies* | 257 (0.144) | 275 (0.015) | 8.91 (7.37-10.76) | 8.20 (6.70-10.02) |
| Encephalitis | 108 (0.060) | 14 (0.001) | 52.62 (28.54-97.01) | 56.28 (25.56-123.91) |
| Meningitis | 42 (0.023) | 23 (0.001) | 16.82 (9.86-28.69) | 17.80 (9.21-34.40) |
| Guillain-Barré syndrome | 4 (0.002) | 25 (0.001) | 1.94 (0.66-5.71) | 1.40 (0.40-4.93) |
| Stroke | 341 (0.191) | 1787 (0.099) | 1.84 (1.61-2.11) | 1.73 (1.49-2.01) |
| **All ocular complications** | 4662 (2.605) | 23079 (1.283) | 2.08 (2.01-2.15) | 1.99 (1.92-2.07) |
| Blepharitis | 402 (0.225) | 2669 (0.148) | 1.48 (1.32-1.66) | 1.40 (1.24-1.59) |
| Conjunctivitis | 1467 (0.820) | 7350 (0.408) | 2.04 (1.92-2.17) | 1.98 (1.86-2.11) |
| Retinitis | 7 (0.004) | 20 (0.001) | 3.25 (1.36-7.77) | 2.15 (0.77-6.01) |
| Optic neuritis | 10 (0.006) | 50 (0.003) | 2.23 (1.07-4.62) | 2.44 (1.10-5.42) |
| Orbital myositis | 13 (0.007) | 8 (0.000) | 26.86 (8.58-84.14) | 23.44 (5.08-108.15) |
| Iritis | 412 (0.230) | 369 (0.021) | 10.05 (8.62-11.73) | 9.32 (7.92-10.96) |
| Keratitis | 365 (0.204) | 421 (0.023) | 8.51 (7.27-9.95) | 8.54 (7.20-10.13) |
| Episcleritis/ Scleritis | 76 (0.042) | 192 (0.011) | 3.93 (2.97-5.19) | 3.78 (2.81-5.07) |
| Glaucoma | 427 (0.239) | 3323 (0.185) | 1.29 (1.16-1.45) | 1.24 (1.10-1.39) |
| Vision loss/Blindness | 5 (0.003) | 30 (0.002) | 2.27 (0.73-7.04) | 2.49 (0.71-8.76) |
| Non-specific eye infection | 39 (0.022) | 59 (0.003) | 6.87 (4.43-10.66) | 7.28 (4.45-11.91) |
| **Dermatological** | 1231 (0.688) | 7207 (0.401) | 1.66 (1.55-1.77) | 1.64 (1.52-1.77) |
| Cellulitis | 1162 (0.649) | 7118 (0.396) | 1.57 (1.46-1.68) | 1.55 (1.44-1.68) |
| Necrotising fasciitis | 5 (0.003) | 7 (0.000) | 8.25 (2.32-29.37) | 5.16 (1.09-24.40) |
| Erysipelas | 64 (0.036) | 94 (0.005) | 7.60 (5.38-10.74) | 8.10 (5.53-11.88) |
| **All visceral complications** | 4719 (2.637) | 33430 (1.858) | 1.41 (1.37-1.46) | 1.30 (1.25-1.35) |
| Septicaemia (bacterial or viral) | 183 (0.102) | 865 (0.048) | 2.04 (1.67-2.50) | 2.02 (1.61-2.53) |
| Pulmonary embolism/DVT | 213 (0.119) | 1332 (0.074) | 1.67 (1.43-1.96) | 1.58 (1.33-1.87) |
| Pneumonia (bacterial or viral) | 3906 (2.183) | 28467 (1.582) | 1.37 (1.32-1.42) | 1.25 (1.20-1.30) |
| Acute hepatitis | 10 (0.006) | 32 (0.002) | 3.60 (1.74-7.48) | 4.97 (1.97-12.50) |
| Acute pancreatitis | 41 (0.023) | 223 (0.012) | 1.79 (1.24-2.58) | 1.83 (1.24-2.70) |
| Acute osteomyelitis | 19 (0.011) | 96 (0.005) | 2.03 (1.16-3.54) | 2.49 (1.19-5.23) |
| Acute pleuritic | 41 (0.023) | 236 (0.013) | 1.66 (1.16-2.36) | 1.65 (1.12-2.44) |
| Gastrointestinal ulcer | 137 (0.077) | 744 (0.041) | 1.92 (1.57-2.33) | 1.77 (1.43-2.18) |
| Peritonitis | 26 (0.015) | 144 (0.008) | 1.75 (1.11-2.75) | 1.61 (0.98-2.66) |
| Myositis | 9 (0.005) | 43 (0.002) | 2.17 (1.00-4.71) | 2.56 (1.05-6.24) |
| Myocarditis | 2 (0.001) | 9 (0.001) | 2.18 (0.47-10.09) | 1.04 (0.09-11.44) |
| Pericarditis | 7 (0.004) | 41 (0.002) | 1.95 (0.85-4.46) | 2.18 (0.89-5.32) |
| Endocarditis | 2 (0.001) | 22 (0.001) | 1.15 (0.26-5.22) | 0.97 (0.17-5.56) |
| HR, hazard ratio. CI, confidence interval. *Not including Ramsay Hunt Syndrome or Post-herpetic neuralgia. **Not including disseminated zoster. ‡ Cox model with age as underlying time scale adjusted for sex, rheumatoid arthritis, inflammatory bowel disease, systemic lupus erythematosus, socioeconomic status, smoking status, body mass index, depression, asthma, chronic obstructive pulmonary disorder, chronic kidney disease, alcoholic liver disease and diabetes. | | | | |

### **Table S3**: Burden and association between zoster and each primary outcome among all immunocompetent adults in CPRD GOLD or Aurum, in the three months following zoster diagnosis.

|  | | | | | |
| --- | --- | --- | --- | --- | --- |
|  |  |  |  |  |  |
|  |  | **Zoster, n (%)** | **Non-zoster, n (%)** | **Age and sex-adjusted HR (95% CI)†** | **Fully adjusted HR (95% CI)‡** |
| **All neurological*** | GOLD | 877 (0.757) | 2387 (0.204) | 3.71 (3.41-4.04) | 3.56 (3.25-3.91) |
|  | AURUM | 283 (0.448) | 740 (0.117) | 4.06 (3.45-4.78) | 3.88 (3.26-4.62) |
|  | GOLD and Aurum | 1160 (0.648) | 3127 (0.174) | 3.78 (3.51-4.08) | 3.63 (3.35-3.94) |
|  | MA of database specific HRs¹ | - | - | - | 3.63 (3.34-3.94) |
| **All ocular** | GOLD | 3581 (3.093) | 18064 (1.545) | 2.05 (1.97-2.14) | 1.97 (1.89-2.05) |
|  | AURUM | 1081 (1.711) | 5015 (0.796) | 2.17 (2.01-2.34) | 2.10 (1.94-2.27) |
|  | GOLD and Aurum | 4662 (2.605) | 23079 (1.283) | 2.08 (2.01-2.15) | 1.99 (1.92-2.07) |
|  | MA of database specific HRs¹ |  |  |  | 2.02 (1.90-2.14) |
| **All cutaneous**** | GOLD | 821 (0.709) | 4862 (0.416) | 1.62 (1.49-1.76) | 1.60 (1.46-1.75) |
|  | AURUM | 410 (0.649) | 2345 (0.372) | 1.75 (1.55-1.98) | 1.73 (1.52-1.98) |
|  | GOLD and Aurum | 1231 (0.688) | 7207 (0.401) | 1.66 (1.55-1.77) | 1.64 (1.52-1.77) |
|  | MA of database specific HRs¹ |  |  |  | 1.64 (1.52-1.77) |
| **All visceral** | GOLD | 3080 (2.660) | 22683 (1.940) | 1.37 (1.31-1.42) | 1.25 (1.20-1.31) |
|  | AURUM | 1639 (2.594) | 10747 (1.706) | 1.52 (1.43-1.62) | 1.40 (1.31-1.49) |
|  | GOLD and Aurum | 4719 (2.637) | 33430 (1.858) | 1.41 (1.37-1.46) | 1.30 (1.25-1.35) |
|  | MA of database specific HRs¹ | - | - | - | 1.32 (1.18-1.47) |
| HR, hazard ratio. CI, confidence interval. *Not including Ramsay Hunt Syndrome or Post-herpetic neuralgia. **Not including disseminated zoster. ‡ Cox model with age as underlying time scale adjusted for sex, rheumatoid arthritis, inflammatory bowel disease, systemic lupus erythematosus, socioeconomic status, smoking status, body mass index, depression, asthma, chronic obstructive pulmonary disorder, chronic kidney disease, alcoholic liver disease and diabetes. ¹Random effects meta-analysis. | | | | | |

### **Table S4**: Burden and association between zoster and each primary outcome among all immunocompetent adults in CPRD GOLD or Aurum, stratified by time since cohort entry.

|  | ***0-3 months*** | | | ***0-6 months*** | | | ***0-12 months*** | | |
| --- | --- | --- | --- | --- | --- | --- | --- | --- | --- |
|  | **Zoster, n (%)** | **Non-zoster, n (%)** | **Fully adjusted HR (95% CI)‡** | **Zoster, n (%)** | **Non-zoster, n (%)** | **Fully adjusted HR (95% CI)‡** | **Zoster, n (%)** | **Non-zoster, n (%)** | **Fully adjusted HR (95% CI)‡** |
| **All neurological*** | 1160 (0.648) | 3127 (0.174) | 3.63 (3.35-3.94) | 1538 (0.859) | 6131 (0.341) | 2.42 (2.26-2.59) | 2212 (1.236) | 11890 (0.661) | 1.79 (1.70-1.89) |
| **All ocular** | 4662 (2.605) | 23079 (1.283) | 1.99 (1.92-2.07) | 7074 (3.953) | 41866 (2.327) | 1.67 (1.62-1.72) | 11070 (6.186) | 73813 (4.102) | 1.48 (1.45-1.51) |
| **All cutaneous**** | 1231 (0.688) | 7207 (0.401) | 1.64 (1.52-1.77) | 1978 (1.105) | 13265 (0.737) | 1.39 (1.31-1.47) | 3352 (1.873) | 24249 (1.348) | 1.30 (1.24-1.36) |
| **All visceral** | 4719 (2.637) | 33430 (1.858) | 1.30 (1.25-1.35) | 8346 (4.664) | 62577 (3.478) | 1.25 (1.22-1.29) | 14609 (8.163) | 113151 (6.288) | 1.22 (1.20-1.25) |
| HR, hazard ratio. CI, confidence interval. *Not including Ramsay Hunt Syndrome or Post-herpetic neuralgia. **Not including disseminated zoster. †Cox model with age as underlying time scale. ‡Additionally adjusted for rheumatoid arthritis, inflammatory bowel disease, systemic lupus erythematosus, socioeconomic status, smoking status, body mass index, depression, asthma, chronic obstructive pulmonary disorder, chronic kidney disease, alcoholic liver disease and diabetes. | | | | | | | | | |

# Definition of severe immunosuppression

Severe immunosuppression was defined as a weakened immune system as the result of conditions or medication. Severely immunosuppressive conditions were determined by the live zoster vaccine (Zostavax) contraindications set out in the UK vaccine guidelines, the Green Book.^1^ As this zoster vaccine is a live vaccine, patients with primary or acquired immunodeficiency are assumed to be at risk of developing a varicella-like or zoster illness from the vaccine virus strain, if vaccinated. Therefore, the contraindications provide a list of conditions considered to result in severe immunosuppression.

Immunosuppressive conditions included: leukaemia, lymphoma, myeloma, other plasma cell dyscrasias, stem cell and bone marrow transplants, solid organ transplants, Human Immunodeficiency Virus infection and cellular immune deficiency. The immunosuppressive treatments will include were: methotrexate, azathioprine, 6-mercaptopurine, biological therapies, steroids, other immunosuppressive agents (e.g. tacrolimus), other disease modifying anti-rheumatic drugs (e.g. ciclosporin), chemotherapy and radiotherapy. Data on immunosuppressive diagnoses and medications were obtained from primary and secondary care records, as per previous studies.^2^

# Code lists

### Zoster

**Read codes**

| medcode | readterm | site |
| --- | --- | --- |
| 390 | Herpes zoster | Site Unspecified |
| 516 | Shingles | Site Unspecified |
| 7331 | Ramsay - Hunt syndrome | Non-truncal (exclud. HZO) |
| 8936 | Ophthalmic herpes zoster infection | HZO |
| 14718 | Herpes zoster with ophthalmic complication | HZO |
| 14793 | Herpes zoster otitis externa | Non-truncal (exclud. HZO) |
| 18918 | Herpes zoster ophthalmicus | HZO |
| 21069 | Herpes zoster with unspecified complication | Site Unspecified |
| 21471 | Herpes zoster NOS | Site Unspecified |
| 25320 | Herpes zoster with dermatitis of eyelid | HZO |
| 27403 | Geniculate herpes zoster | Non-truncal (exclud. HZO) |
| 27546 | Herpes zoster with keratoconjunctivitis | HZO |
| 31681 | Herpes zoster - otitis externa | Non-truncal (exclud. HZO) |
| 33810 | Herpes zoster with other ophthalmic complication | HZO |
| 38531 | Herpes zoster with other specified complication NOS | Site Unspecified |
| 39692 | Polyneuropathy in herpes zoster | Site Unspecified |
| 43235 | Herpes zoster with other specified complication | Site Unspecified |
| 44944 | Herpes zoster with meningitis | Non-truncal (exclud. HZO) |
| 47375 | Zoster encephalitis | Site Unspecified |
| 50537 | Herpes zoster with other CNS complications | Non-truncal (exclud. HZO) |
| 51692 | Encephalitis due to herpes zoster | Site Unspecified |
| 52126 | Herpes zoster with other central nervous system complication | Non-truncal (exclud. HZO) |
| 52319 | Disseminated zoster | Site Unspecified |
| 55940 | Herpes zoster iridocyclitis | HZO |
| 57895 | Herpes zoster meningitis | Non-truncal (exclud. HZO) |
| 62558 | Infective otitis externa due to herpes zoster | Non-truncal (exclud. HZO) |
| 63739 | Herpes zoster with other CNS complication NOS | Non-truncal (exclud. HZO) |
| 69405 | Herpes zoster encephalitis | Site Unspecified |
| 70197 | [X]Zoster without complications | Site Unspecified |
| 71464 | Meningitis due to herpes zoster virus | Non-truncal (exclud. HZO) |
| 105157 | Hutchinson's sign - herpes zoster involving nose tip | HZO |

**CPRD Aurum codes**

| medcodeid | term | site |
| --- | --- | --- |
| 801821000033116 | *herpes zoster | Site Unspecified |
| 18244018 | herpes zoster with meningitis | Non-truncal (exclud. HZO) |
| 286722010 | herpes zoster with unspecified complication | Site Unspecified |
| 822331000006110 | herpes zoster with other cns complications | Site Unspecified |
| 1219080012 | herpes zoster meningitis | Non-truncal (exclud. HZO) |
| 625341000006116 | disseminated zoster | Site Unspecified |
| 822201000006110 | herpes zoster - otitis externa | Non-truncal (exclud. HZO) |
| 1139221000000111 | hutchinson's sign - herpes zoster involving nose tip | HZO |
| 395349015 | herpes zoster with other central nervous system complication | Site Unspecified |
| 18585010 | herpes zoster iridocyclitis | HZO |
| 36819018 | geniculate herpes zoster | Non-truncal (exclud. HZO) |
| 288153012 | [x]zoster with other complications | Site Unspecified |
| 191651000006117 | ramsay - hunt syndrome | Non-truncal (exclud. HZO) |
| 145090014 | herpes zoster ophthalmicus | HZO |
| 988701000006119 | ophthalmic herpes zoster | HZO |
| 344958013 | herpes zoster encephalitis | Site Unspecified |
| 44081000006115 | ophthalmic herpes zoster infection | HZO |
| 8904017 | shingles | Site Unspecified |
| 286718017 | herpes zoster with other specified complication | Site Unspecified |
| 344957015 | zoster encephalitis | Site Unspecified |
| 8902018 | herpes zoster | Site Unspecified |
| 640911000006110 | encephalitis due to herpes zoster | Site Unspecified |
| 286723017 | herpes zoster nos | Site Unspecified |
| 288154018 | [x]zoster without complications | Site Unspecified |
| 879151000006112 | other herpes zoster | Site Unspecified |
| 905671000006114 | [rfc] shingles | Site Unspecified |
| 1229869010 | herpes zoster with keratoconjunctivitis | HZO |
| 286717010 | herpes zoster with other ophthalmic complication | HZO |
| 286715019 | herpes zoster with dermatitis of eyelid | HZO |
| 481174015 | herpes zoster otitis externa | Non-truncal (exclud. HZO) |
| 879141000006110 | ophthalmic herpes zoster | HZO |
| 706441000006117 | meningitis due to herpes zoster virus | Non-truncal (exclud. HZO) |
| 778951000006117 | infective otitis externa due to herpes zoster | Non-truncal (exclud. HZO) |
| 286714015 | herpes zoster with ophthalmic complication | HZO |
| 822321000006112 | herpes zoster with other cns complication nos | Site Unspecified |
| 905931000006117 | [rfc] shingles | Site Unspecified |
| 286721015 | herpes zoster with other specified complication nos | Site Unspecified |

**ICD-10 codes**

| code | description | site |
| --- | --- | --- |
| B02.1 | Zoster meningitis | Non-truncal (exclud. HZO) |
| B02.9 | Zoster without complication | Site Unspecified |
| B02.7 | Disseminated zoster | Site Unspecified |
| B02.0 | Zoster encephalitis | Site Unspecified |
| B02 | Zoster [herpes zoster] | Site Unspecified |
| B02.8 | Zoster with other complications | Site Unspecified |
| B02.3 | Zoster ocular disease | HZO |

### Postherpetic Neuralgia

**Read codes**

| medcode | readterm |
| --- | --- |
| 1598 | Post-herpetic neuralgia |
| 7584 | Post-herpetic trigeminal neuralgia |
| 10223 | Postherpetic neuralgia |
| 11498 | Postherpetic trigeminal neuralgia |
| 17180 | Postzoster neuralgia |
| 31709 | Postherpetic polyneuropathy |

**CPRD Aurum codes**

| medcodeid | term |
| --- | --- |
| 297557016 | polyneuropathy in herpes zoster |
| 30355011 | post-herpetic trigeminal neuralgia |
| 4765019 | postherpetic neuralgia |
| 212551000006110 | postzoster neuralgia |
| 211231000006113 | postherpetic polyneuropathy |
| 217001000006117 | post-herpetic neuralgia |
| 211241000006115 | postherpetic trigeminal neuralgia |

**ICD-10 codes**

| code | description |
| --- | --- |
| G53.0 | Postzoster neuralgia |
| B02.2 | Zoster with other nervous system involvement |

### Ramsay-Hunt syndrome

**Read codes**

| medcode | readterm | zoster_spec_code |
| --- | --- | --- |
| 32335 | Geniculate ganglionitis |  |
| 7331 | Ramsay - Hunt syndrome |  |

**CPRD Aurum codes**

| medcodeid | term | zoster_spec_code |
| --- | --- | --- |
| 120984019 | geniculate ganglionitis |  |
| 191651000006117 | ramsay - hunt syndrome |  |

**ICD-10 codes**

None

## Neurological complications

#### Definitions

| **Outcome** | **Definition** |
| --- | --- |
| [Encephalitis](#_Toc522795070) | Codes within 12 months following zoster, and no record of the condition in the 4 weeks prior to zoster. |
| [Transverse myelitis](#_Toc522795071) |  |
| [Meningitis](#_Toc522795072) |  |
| Guillain-Barré syndrome |  |
| [Other cranial and peripheral nerve palsies](#_Toc522795069) |  |
| [First ever stroke](#_Toc522795073) |  |

### Other cranial and peripheral nerve palsies

**Read codes**

| medcode | readterm | zoster_spec_code |
| --- | --- | --- |
| 110277 | O/E - cranials 9+10 paralysis |  |
| 2073 | Acute radial nerve palsy |  |
| 97358 | O/E-cranial nerve 12 paralysis |  |
| 89932 | Conjugate gaze palsy |  |
| 44022 | O/E - cranial nerve 6 - palsy |  |
| 66260 | O/E - cranials 9 + 10 palsy |  |
| 112597 | O/E - cranial nerve 11 - palsy |  |
| 45302 | O/E -cranial nerve 7-palsy-LMN |  |
| 31140 | Third nerve palsy - total |  |
| 37984 | Pupillary paralysis |  |
| 73518 | O/E - cranial nerve 12 - palsy |  |
| 26083 | Partial oculomotor nerve palsy |  |
| 27593 | Third nerve palsy - partial |  |
| 15710 | Total oculomotor nerve palsy |  |
| 46127 | Multiple cranial nerve palsies NOS |  |
| 23665 | Trochlear (fourth) nerve palsy |  |
| 62900 | O/E -cranial nerve -palsy -UMN |  |
| 94255 | O/E -cranial nerve 3-paralysis |  |
| 4494 | Abducens (sixth) nerve palsy |  |
| 63709 | O/E -cranial nerve 6-paralysis |  |
| 65247 | O/E - cranial nerve 5 - palsy |  |
| 27792 | O/E - cranial nerve 3 - palsy |  |
| 98631 | O/E -cranial 7 -paralysis -LMN |  |
| 54229 | Multiple cranial nerve palsies |  |
| 98772 | Convergence palsy |  |
| 52102 | O/E - cranial nerve 4 - palsy |  |
| 56968 | O/E - cranial 7 -paralysis-UMN |  |
| 206 | Bell's (facial) palsy |  |

**CPRD Aurum codes**

| medcodeid | term | zoster_spec_code |
| --- | --- | --- |
| 298822011 | partial oculomotor nerve palsy |  |
| 88511000006119 | trochlear (fourth) nerve palsy |  |
| 255478012 | o/e -cranial nerve -palsy -umn |  |
| 255484010 | o/e - cranials 9 + 10 palsy |  |
| 8879010 | acute radial nerve palsy |  |
| 255479016 | o/e - cranial 7 -paralysis-umn |  |
| 255463017 | o/e -cranial nerve 4-paralysis |  |
| 695681000006110 | multiple cranial nerve palsies |  |
| 255472013 | o/e -cranial nerve 5-paralysis |  |
| 298824012 | third nerve palsy - partial |  |
| 255485011 | o/e - cranials 9+10 paralysis |  |
| 298826014 | third nerve palsy - total |  |
| 399041000006118 | [x]multip cranial nerve palsies/infectous+parasitc diseas ce |  |
| 504611000006114 | bell's (facial) palsy |  |
| 255462010 | o/e - cranial nerve 4 - palsy |  |
| 297442013 | multiple cranial nerve palsies nos |  |
| 255491013 | o/e-cranial nerve 12 paralysis |  |
| 1786558018 | abducens (sixth) nerve palsy |  |
| 298844018 | convergence palsy |  |
| 255466013 | o/e -cranial nerve 6-paralysis |  |
| 477321015 | conjugate gaze palsy |  |
| 255459012 | o/e - cranial nerve 3 - palsy |  |
| 255475010 | o/e -cranial 7 -paralysis -lmn |  |
| 255465012 | o/e - cranial nerve 6 - palsy |  |
| 255471018 | o/e - cranial nerve 5 - palsy |  |
| 255474014 | o/e -cranial nerve 7-palsy-lmn |  |
| 255488013 | o/e-cranial nerve 11 paralysis |  |
| 255460019 | o/e -cranial nerve 3-paralysis |  |
| 298827017 | total oculomotor nerve palsy |  |
| 255487015 | o/e - cranial nerve 11 - palsy |  |
| 255490014 | o/e - cranial nerve 12 - palsy |  |
| 9909019 | pupillary paralysis |  |

**ICD-10 codes**

| code | description | zoster_spec_code |
| --- | --- | --- |
| H49.2 | Sixth [abducent] nerve palsy |  |
| H49.0 | Third [oculomotor] nerve palsy |  |
| G53.1 | Multiple cranial nerve palsies in infectious and parasitic diseases classified elsewhere |  |
| G51.0 | Bell palsy |  |
| H49.1 | Fourth [trochlear] nerve palsy |  |
| H51.0 | Palsy of conjugate gaze |  |

### Encephalitis

**Read codes**

| medcode | readterm | zoster_spec_code |
| --- | --- | --- |
| 29781 | Late effects of viral encephalitis |  |
| 36335 | Postvaricella encephalitis | 1 |
| 104680 | [X]Encephalitis,myelitis+encephalomyelitis/viral disease CE |  |
| 29901 | Post-encephalitic syndrome |  |
| 48018 | Postinfectious encephalitis |  |
| 45838 | Post-encephalitis syndrome |  |
| 4311 | Herpetic meningoencephalitis |  |
| 99730 | [X]Encephalitis,myelitis+encephalomyelitis/other diseases CE |  |
| 2423 | Encephalitis NOS |  |
| 42240 | Encephalitis in viral disease NOS |  |
| 35344 | Unspecified encephalitis due to other infection EC |  |
| 65624 | Postinfectious encephalitis NOS |  |
| 69405 | Herpes zoster encephalitis | 1 |
| 51105 | Postencephalitic parkinsonism |  |
| 100599 | [X]Other specified viral encephalitis |  |
| 47375 | Zoster encephalitis | 1 |
| 110877 | [X]Sequelae of viral encephalitis |  |
| 51692 | Encephalitis due to herpes zoster | 1 |
| 51384 | Encephalitis due to varicella | 1 |
| 100360 | Post-encephalitic seborrhoea |  |
| 35375 | Encephalitis due to other infection EC |  |
| 58574 | Encephalitis in viral disease EC |  |
| 50084 | [X]Postencephalitic syndrome |  |
| 5398 | Viral encephalitis NOS |  |
| 73146 | [X]Unspecified viral encephalitis |  |
| 53080 | Other causes of encephalitis |  |

**CPRD Aurum codes**

| medcodeid | term | zoster_spec_code |
| --- | --- | --- |
| 61171000006112 | viral encephalitis nos |  |
| 641091000006111 | encephalitis due to varicella | 1 |
| 296867011 | unspecified encephalitis due to other infection ec |  |
| 175040015 | late effects of viral encephalitis |  |
| 296856010 | encephalitis due to other infection ec |  |
| 423111000006119 | [x]postencephalitic syndrome |  |
| 211311000006111 | postinfectious encephalitis nos |  |
| 882881000006114 | encephalitis/myelitis |  |
| 640911000006110 | encephalitis due to herpes zoster | 1 |
| 426441000006115 | [x]sequelae of viral encephalitis |  |
| 296836014 | encephalitis in viral disease ec |  |
| 286691018 | postvaricella encephalitis | 1 |
| 296852012 | encephalitis in viral disease nos |  |
| 288125012 | [x]other specified viral encephalitis |  |
| 67837015 | post-encephalitic syndrome |  |
| 211301000006113 | postinfectious encephalitis |  |
| 309152014 | post-encephalitic seborrhoea |  |
| 379721000006117 | [x]encephalitis,myelitis+encephalomyelitis/other diseases ce |  |
| 379731000006119 | [x]encephalitis,myelitis+encephalomyelitis/viral disease ce |  |
| 344957015 | zoster encephalitis | 1 |
| 344958013 | herpes zoster encephalitis | 1 |
| 399392017 | encephalitis nos |  |
| 399391012 | other causes of encephalitis |  |
| 1229666017 | post-encephalitis syndrome |  |
| 33622011 | postencephalitic parkinsonism |  |
| 16921016 | herpetic meningoencephalitis |  |
| 288126013 | [x]unspecified viral encephalitis |  |
| 882891000006112 | postinfective encephalitis |  |
| 882871000006111 | encephalitis/myelitis nos |  |

**ICD-10 codes**

| code | description | zoster_spec_code |
| --- | --- | --- |
| A85 | Other viral encephalitis, not elsewhere classified |  |
| G04.0 | Acute disseminated encephalitis |  |
| B02.0 | Zoster encephalitis | 1 |
| G21.3 | Postencephalitic parkinsonism |  |
| A86 | Unspecified viral encephalitis |  |
| A85.8 | Other specified viral encephalitis |  |
| B00.4 | Herpesviral encephalitis |  |
| B94.1 | Sequelae of viral encephalitis |  |
| B01.1 | Varicella encephalitis | 1 |
| F07.1 | Postencephalitic syndrome |  |

### Transverse myelitis

**Read codes**

| medcode | readterm | zoster_spec_code |
| --- | --- | --- |
| 6494 | Transverse myelitis |  |
| 92694 | Varicella transverse myelitis | 1 |
| 5463 | Transverse myelitis |  |

**CPRD Aurum codes**

| medcodeid | term | zoster_spec_code |
| --- | --- | --- |
| 91711000006118 | transverse myelitis |  |
| 28148010 | transverse myelitis |  |
| 344996014 | varicella transverse myelitis | 1 |

**ICD-10 codes**

| code | description |
| --- | --- |
| G37.3 | Acute transverse myelitis in demyelinating disease of central nervous system |

### Meningitis

**Read codes**

| medcode | readterm | zoster_spec_code |
| --- | --- | --- |
| 61924 | [X]Meningitis due to other specified causes |  |
| 11929 | Meningitis - viral NOS |  |
| 3945 | Meningitis of unspecified cause |  |
| 2386 | Unspecified meningitis |  |
| 50054 | [X]Other viral meningitis |  |
| 57895 | Herpes zoster meningitis | 1 |
| 65853 | Other specified viral meningitis |  |
| 27627 | Meningitis due to other organisms |  |
| 37292 | [X]Viral meningitis, unspecified |  |
| 41811 | Other viral meningitis |  |
| 71464 | Meningitis due to herpes zoster virus | 1 |
| 100619 | [X]Meningitis/other specifd infectious+parasitic diseases CE |  |
| 7946 | Notification of acute meningit |  |
| 44944 | Herpes zoster with meningitis | 1 |
| 2169 | Viral meningitis NOS |  |
| 6902 | Meningitis due to viral organisms EC |  |
| 43411 | Varicella meningitis | 1 |
| 100075 | [X]Meningitis in viral diseases classified elsewhere |  |
| 34412 | Meningitis due to organism NOS |  |

**CPRD Aurum codes**

| medcodeid | term | zoster_spec_code |
| --- | --- | --- |
| 706441000006117 | meningitis due to herpes zoster virus | 1 |
| 296826018 | meningitis of unspecified cause |  |
| 1219080012 | herpes zoster meningitis | 1 |
| 882861000006116 | other meningitis nos |  |
| 882851000006118 | meningitis |  |
| 1219078018 | varicella meningitis | 1 |
| 296541000006111 | notification of acute meningit |  |
| 299292015 | [x]meningitis due to other specified causes |  |
| 288129018 | [x]viral meningitis, unspecified |  |
| 296825019 | meningitis due to organism nos |  |
| 925751000006117 | notification of acute meningit |  |
| 286654012 | other specified viral meningitis |  |
| 395347018 | viral meningitis nos |  |
| 288127016 | [x]other viral meningitis |  |
| 18244018 | herpes zoster with meningitis | 1 |
| 988971000006110 | meningitis |  |
| 299289019 | [x]meningitis in viral diseases classified elsewhere |  |
| 296815012 | other viral meningitis |  |
| 906821000006117 | [rfc] meningitis |  |
| 706611000006119 | meningitis due to viral organisms ec |  |
| 296830015 | unspecified meningitis |  |
| 296804019 | meningitis due to other organisms |  |
| 397401000006115 | [x]meningitis/other specifd infectious+parasitic diseases ce |  |
| 399389016 | meningitis - viral nos |  |

**ICD-10 codes**

| code | description | zoster_spec_code |
| --- | --- | --- |
| A87.8 | Other viral meningitis |  |
| G03 | Meningitis due to other and unspecified causes |  |
| G02.0 | Meningitis in viral diseases classified elsewhere |  |
| G03.9 | Meningitis, unspecified |  |
| G02.8 | Meningitis in other specified infectious and parasitic diseases classified elsewhere |  |
| B02.1 | Zoster meningitis | 1 |
| A87 | Viral meningitis |  |
| A87.9 | Viral meningitis, unspecified |  |
| G03.8 | Meningitis due to other specified causes |  |
| G02 | Meningitis in other infectious and parasitic diseases classified elsewhere |  |
| B01.0 | Varicella meningitis | 1 |
| B00.3 | Herpesviral meningitis |  |

### Guillain-Barré syndrome

**Read codes**

| medcode | readterm | zoster_spec_code |
| --- | --- | --- |
| 6376 | Acute infective polyneuritis |  |
| 28294 | Polyneuritis cranialis |  |
| 24216 | Postinfectious polyneuritis |  |
| 63544 | Acute infective polyneuritis NOS |  |
| 1607 | Guillain-Barre syndrome |  |

**Aurum codes**

| medcodeid | term | zoster_spec_code |
| --- | --- | --- |
| 94456015 | polyneuritis cranialis |  |
| 297539017 | acute infective polyneuritis nos |  |
| 68321016 | guillain-barre syndrome |  |
| 211331000006117 | postinfectious polyneuritis |  |
| 207847010 | acute infective polyneuritis |  |

**ICD-10 codes**

| code | description | zoster_spec_code |
| --- | --- | --- |
| G61.0 | Guillain-BarrÃ© syndrome |  |

### Stroke

**Read codes**

| medcode | readterm |
| --- | --- |
| 569 | Infarction - cerebral |
| 1298 | CVA unspecified |
| 1469 | Stroke and cerebrovascular accident unspecified |
| 1786 | Subarachnoid haemorrhage |
| 3149 | Cerebral infarction NOS |
| 3535 | Intracerebral haemorrhage NOS |
| 4152 | Thrombosis, carotid artery |
| 4240 | Carotid artery occlusion |
| 5051 | Intracerebral haemorrhage |
| 5185 | Lateral medullary syndrome |
| 5363 | CVA - cerebral artery occlusion |
| 5602 | Cerebellar infarction |
| 6116 | CVA - Cerebrovascular accident unspecified |
| 6155 | Stroke due to cerebral arterial occlusion |
| 6253 | Stroke unspecified |
| 6960 | CVA - cerebrovascular accid due to intracerebral haemorrhage |
| 7780 | Left sided CVA |
| 7912 | Pontine haemorrhage |
| 8443 | Brain stem stroke syndrome |
| 8837 | Cerebral arterial occlusion |
| 9696 | Subarachnoid haemorrhage from posterior communicating artery |
| 9985 | Left sided cerebral infarction |
| 10504 | Right sided cerebral infarction |
| 12833 | Right sided CVA |
| 13564 | Cerebellar haemorrhage |
| 15019 | Cerebral embolism |
| 15252 | Brainstem infarction NOS |
| 16517 | Cerebral thrombosis |
| 17322 | Cerebellar stroke syndrome |
| 17326 | Subarachnoid haemorrh from intracranial artery, unspecif |
| 18604 | Stroke due to intracerebral haemorrhage |
| 18689 | Middle cerebral artery syndrome |
| 19201 | Right sided intracerebral haemorrhage, unspecified |
| 19260 | Posterior cerebral artery syndrome |
| 19280 | Anterior cerebral artery syndrome |
| 19412 | Subarachnoid haemorrhage from middle cerebral artery |
| 20284 | Intracranial haemorrhage NOS |
| 23580 | Subarachnoid haemorrhage NOS |
| 23671 | Cerebral infarct due to thrombosis of precerebral arteries |
| 24446 | Cerebral infarction due to embolism of precerebral arteries |
| 25615 | Brainstem infarction |
| 26424 | Infarction of basal ganglia |
| 27975 | Cerebral infarction due to embolism of cerebral arteries |
| 28314 | Left sided intracerebral haemorrhage, unspecified |
| 28807 | Subarachnoid haemorrhage following injury |
| 29939 | Ruptured berry aneurysm |
| 30045 | External capsule haemorrhage |
| 30202 | Intracerebral haemorrhage, intraventricular |
| 31060 | Intracerebral haemorrhage in hemisphere, unspecified |
| 31595 | Cortical haemorrhage |
| 31805 | Other and unspecified intracranial haemorrhage |
| 32447 | Basilar artery occlusion |
| 33499 | Pure motor lacunar syndrome |
| 33543 | Cerebrl infarctn due/unspcf occlusn or sten/cerebrl artrs |
| 34758 | Cerebral embolus |
| 36717 | Cerebral infarction due to thrombosis of cerebral arteries |
| 38304 | Closed traumatic subarachnoid haemorrhage |
| 39344 | Cereb infarct due cerebral venous thrombosis, nonpyogenic |
| 40338 | Internal capsule haemorrhage |
| 40758 | Cereb infarct due unsp occlus/stenos precerebr arteries |
| 40847 | Vertebral artery occlusion |
| 41910 | Subarachnoid haemorrhage from basilar artery |
| 42331 | Subarachnoid haemorrhage from anterior communicating artery |
| 45781 | Precerebral arterial occlusion |
| 46316 | Basal nucleus haemorrhage |
| 47607 | CVA - cerebrovascular accident in the puerperium |
| 47642 | Wallenberg syndrome |
| 51326 | Other precerebral artery occlusion |
| 51759 | Occlusion and stenosis of middle cerebral artery |
| 51767 | Pure sensory lacunar syndrome |
| 53745 | [X]Other cerebral infarction |
| 53810 | [X]Other intracerebral haemorrhage |
| 55602 | Occlusion and stenosis of cerebellar arteries |
| 56007 | Subarachnoid haemorrhage from carotid siphon and bifurcation |
| 56279 | Stroke in the puerperium |
| 57315 | Intracerebral haemorrhage, multiple localized |
| 57495 | Infarction - precerebral |
| 57527 | Occlusion and stenosis of anterior cerebral artery |
| 58545 | Traumatic subarachnoid haemorrhage |
| 60692 | Subarachnoid haemorrhage from vertebral artery |
| 62342 | Bulbar haemorrhage |
| 65745 | [X]Other subarachnoid haemorrhage |
| 65770 | Occlusion and stenosis of posterior cerebral artery |
| 70536 | Acute cerebrovascular insufficiency NOS |
| 71274 | Occlusion+stenosis of multiple and bilat cerebral arteries |
| 71585 | Precerebral artery occlusion NOS |
| 90572 | [X]Occlusion and stenosis of other precerebral arteries |
| 91627 | [X]Cerebrl infarctn due/unspcf occlusn or sten/cerebrl artrs |
| 92036 | [X]Occlusion and stenosis of other cerebral arteries |
| 93459 | [X]Other lacunar syndromes |
| 94482 | [X]Cereb infarct due unsp occlus/stenos precerebr arteries |
| 96630 | [X]Intracerebral haemorrhage in hemisphere, unspecified |
| 96717 | Open traumatic subarachnoid haemorrhage |
| 98642 | Multiple and bilateral precerebral arterial occlusion |
| 107440 | Lobar cerebral haemorrhage |
| 108630 | [X]Subarachnoid haemorrh from intracranial artery, unspecif |
| 108668 | [X]Subarachnoid haemorrhage from other intracranial arteries |

**CPRD Aurum codes**

| medcodeid | term |
| --- | --- |
| 118689010 | Cerebral thrombosis |
| 125470015 | Cerebral embolism |
| 130375018 | Lateral medullary syndrome |
| 158118014 | Cerebellar infarction |
| 299342019 | [X]Other lacunar syndromes |
| 300242011 | Ruptured berry aneurysm |
| 300244012 | Subarachnoid haemorrhage from carotid siphon and bifurcation |
| 300253017 | Subarachnoid haemorrhage from vertebral artery |
| 300257016 | Subarachnoid haemorrhage NOS |
| 300276019 | External capsule haemorrhage |
| 300277011 | Intracerebral haemorrhage, intraventricular |
| 300287010 | Intracerebral haemorrhage NOS |
| 300290016 | Other and unspecified intracranial haemorrhage |
| 300298011 | Intracranial haemorrhage NOS |
| 300303013 | Basilar artery occlusion |
| 300309012 | Vertebral artery occlusion |
| 300310019 | Multiple and bilateral precerebral arterial occlusion |
| 300311015 | Other precerebral artery occlusion |
| 300312010 | Cerebral infarct due to thrombosis of precerebral arteries |
| 300313017 | Cerebral infarction due to embolism of precerebral arteries |
| 300314011 | Precerebral artery occlusion NOS |
| 300321011 | Cerebral infarction due to thrombosis of cerebral arteries |
| 300322016 | Cerebral infarction due to embolism of cerebral arteries |
| 300362017 | Middle cerebral artery syndrome |
| 300363010 | Anterior cerebral artery syndrome |
| 300364016 | Posterior cerebral artery syndrome |
| 300366019 | Cerebellar stroke syndrome |
| 300370010 | Left sided CVA |
| 300371014 | Right sided CVA |
| 300380014 | Acute cerebrovascular insufficiency NOS |
| 300395011 | Occlusion and stenosis of middle cerebral artery |
| 300396012 | Occlusion and stenosis of anterior cerebral artery |
| 300398013 | Occlusion and stenosis of posterior cerebral artery |
| 300399017 | Occlusion and stenosis of cerebellar arteries |
| 300935019 | [X]Subarachnoid haemorrhage from other intracranial arteries |
| 300936018 | [X]Other subarachnoid haemorrhage |
| 300939013 | [X]Other intracerebral haemorrhage |
| 300956017 | [X]Intracerebral haemorrhage in hemisphere, unspecified |
| 320735017 | Open traumatic subarachnoid haemorrhage |
| 345639010 | Infarction - precerebral |
| 345650013 | Brainstem infarction NOS |
| 345655015 | Pure motor lacunar syndrome |
| 345658018 | Pure sensory lacunar syndrome |
| 345675012 | Lobar cerebral haemorrhage |
| 391042012 | Subarachnoid haemorrhage following injury |
| 391043019 | Traumatic subarachnoid haemorrhage |
| 395777014 | Precerebral arterial occlusion |
| 395778016 | Carotid artery occlusion |
| 395780010 | Cerebral infarction NOS |
| 402929011 | Closed traumatic subarachnoid haemorrhage |
| 405339016 | Stroke and cerebrovascular accident unspecified |
| 411416011 | Stroke in the puerperium |
| 451133011 | Left sided cerebral infarction |
| 451134017 | Right sided cerebral infarction |
| 481028017 | Subarachnoid haemorrhage |
| 495394013 | Cortical haemorrhage |
| 496232015 | Internal capsule haemorrhage |
| 502878012 | Cerebellar haemorrhage |
| 503469016 | Pontine haemorrhage |
| 505324014 | Traumatic cerebral haemorrhage |
| 1212072018 | Occlusive stroke |
| 1222398015 | Cerebral arterial occlusion |
| 2474651019 | Infarction of basal ganglia |
| 57341000006119 | Wallenberg syndrome |
| 100771000006112 | Thrombosis, carotid artery |
| 122361000006113 | Stroke due to cerebral arterial occlusion |
| 122371000006118 | Stroke due to intracerebral haemorrhage |
| 122401000006115 | Stroke unspecified |
| 123441000006112 | Subarachnoid haemorrh from intracranial artery, unspecif |
| 123481000006118 | Subarachnoid haemorrhage from anterior communicating artery |
| 123491000006115 | Subarachnoid haemorrhage from basilar artery |
| 123511000006114 | Subarachnoid haemorrhage from middle cerebral artery |
| 123521000006118 | Subarachnoid haemorrhage from posterior communicating artery |
| 163261000006119 | Right sided intracerebral haemorrhage, unspecified |
| 218511000000117 | Infarction - cerebral |
| 267311000006118 | Occlusion+stenosis of multiple and bilat cerebral arteries |
| 370701000006118 | [X]Cerebrl infarctn due/unspcf occlusn or sten/cerebrl artrs |
| 428181000006115 | [X]Subarachnoid haemorrh from intracranial artery, unspecif |
| 503791000006114 | Basal nucleus haemorrhage |
| 524511000006116 | Brain stem stroke syndrome |
| 524541000006117 | Brainstem infarction |
| 542251000006112 | Cereb infarct due cerebral venous thrombosis, nonpyogenic |
| 542831000006116 | Cerebral embolus |
| 543141000006110 | Cerebrl infarctn due/unspcf occlusn or sten/cerebrl artrs |
| 605461000006117 | CVA - cerebral artery occlusion |
| 605471000006112 | CVA - cerebrovascular accid due to intracerebral haemorrhage |
| 605481000006110 | CVA - cerebrovascular accident in the puerperium |
| 605491000006113 | CVA - Cerebrovascular accident unspecified |
| 605501000006117 | CVA unspecified |
| 744901000006114 | Intracerebral haemorrhage |
| 744921000006116 | Intracerebral haemorrhage in hemisphere, unspecified |
| 746571000006116 | Intracerebral haemorrhage, multiple localized |
| 748941000006115 | Left sided intracerebral haemorrhage, unspecified |
| 884421000006119 | Cerebral haemorrhage |
| 884451000006111 | Cerebral haemorrhage NOS |
| 884501000006113 | Cerebral A. occlusion NOS |
| 884521000006115 | Stroke/CVA - undefined |
| 884531000006117 | Stroke |
| 907581000006119 | [RFC] Stroke/CVA |
| 907591000006116 | [RFC] Stroke |
| 908801000006114 | [RFC] Stroke |
| 989201000006117 | Cerebral haemorrhage |
| 989211000006119 | Cerebral haemorrhage NOS |
| 1573101000006112 | Cerebral infarction with haemorrhagic transformation |
| 1576261000006112 | Cause of Death- Cerebral Infarct |
| 1729331000006116 | Cerebral venous thrombosis |
| 1900651000006110 | SCPE class predom patt C.3 infarct of middle cerebral artery |
| 300941014 | [X]Other cerebral infarction |
| 300942019 | [X]Occlusion and stenosis of other precerebral arteries |
| 300943012 | [X]Occlusion and stenosis of other cerebral arteries |
| 483988011 | Bulbar haemorrhage |
| 370661000006114 | [X]Cereb infarct due unsp occlus/stenos precerebr arteries |
| 542261000006114 | Cereb infarct due unsp occlus/stenos precerebr arteries |

**ICD-10 codes**

| code | description |
| --- | --- |
| I60 | Subarachnoid haemorrhage |
| I60.0 | Subarachnoid haemorrhage from carotid siphon and bifurcation |
| I60.1 | Subarachnoid haemorrhage from middle cerebral artery |
| I60.2 | Subarachnoid haemorrhage from anterior communicating artery |
| I60.3 | Subarachnoid haemorrhage from posterior communicating artery |
| I60.4 | Subarachnoid haemorrhage from basilar artery |
| I60.5 | Subarachnoid haemorrhage from vertebral artery |
| I60.6 | Subarachnoid haemorrhage from other intracranial arteries |
| I60.7 | Subarachnoid haemorrhage from intracranial artery, unspecified |
| I60.8 | Other subarachnoid haemorrhage |
| I60.9 | Subarachnoid haemorrhage, unspecified |
| I61 | Intracerebral haemorrhage |
| I61.0 | Intracerebral haemorrhage in hemisphere, subcortical |
| I61.1 | Intracerebral haemorrhage in hemisphere, cortical |
| I61.2 | Intracerebral haemorrhage in hemisphere, unspecified |
| I61.3 | Intracerebral haemorrhage in brain stem |
| I61.4 | Intracerebral haemorrhage in cerebellum |
| I61.5 | Intracerebral haemorrhage, intraventricular |
| I61.6 | Intracerebral haemorrhage, multiple localized |
| I61.8 | Other intracerebral haemorrhage |
| I61.9 | Intracerebral haemorrhage, unspecified |
| I63 | Cerebral infarction |
| I63.0 | Cerebral infarction due to thrombosis of precerebral arteries |
| I63.1 | Cerebral infarction due to embolism of precerebral arteries |
| I63.2 | Cerebral infarction due to unspecified occlusion or stenosis of precerebral arteries |
| I63.3 | Cerebral infarction due to thrombosis of cerebral arteries |
| I63.4 | Cerebral infarction due to embolism of cerebral arteries |
| I63.5 | Cerebral infarction due to unspecified occlusion or stenosis of cerebral arteries |
| I63.6 | Cerebral infarction due to cerebral venous thrombosis, nonpyogenic |
| I63.8 | Other cerebral infarction |
| I63.9 | Cerebral infarction, unspecified |
| I64 | Stroke, not specified as haemorrhage or infarction |

## Ophthalmic complications

#### Definitions

| **Outcome** | **Definition** |
| --- | --- |
| Blepharitis | A diagnosis of the acute eye condition within 12 month of zoster or first ever evidence of the eye condition becoming chronic within 12 months. |
| Conjunctivitis |  |
| Retinitis |  |
| Optic neuritis |  |
| Orbital myositis |  |
| Iritis | A diagnosis of the acute eye condition within 12 month of zoster; or first ever evidence of the eye condition becoming chronic within 12 months; or evidence of having had surgery for the eye condition within 12 months. |
| Keratitis |  |
| Episcleritis/ Scleritis |  |
| Glaucoma |  |
| Vision loss/Blindness | A zoster-related blinding complication (iritis, glaucoma, optic neuritis or retinitis – see above) followed by a blindness/loss of vision code within 12 months of zoster. |
| Non-specific eye infection | Diagnostic code or treatment (with topical eye-specific antibiotics or antivirals) within 1 month of zoster. |

### Blepharitis

**Read codes**

| medcode | readterm | zoster_spec_code |
| --- | --- | --- |
| 266 | Blepharitis |  |
| 2168 | Blepharoconjunctivitis |  |
| 3544 | Unspecified blepharitis |  |
| 6875 | Squamous blepharitis |  |
| 7620 | Inflammation of eyelids |  |
| 11108 | Meibomianitis |  |
| 15492 | Eyelid inflammation NOS |  |
| 15611 | Blepharitis NOS |  |
| 19990 | Ulcerative blepharitis |  |
| 28303 | Blepharoconjunctivitis NOS |  |
| 38644 | Other eyelid inflammation |  |
| 101554 | [X]Other specified inflammation of eyelid |  |

**CPRD Aurum codes**

| medcodeid | term | zoster_spec_code |
| --- | --- | --- |
| 69126011 | blepharitis |  |
| 97906011 | squamous blepharitis |  |
| 114036017 | blepharoconjunctivitis |  |
| 151866011 | ulcerative blepharitis |  |
| 298484019 | unspecified blepharoconjunctivitis |  |
| 298487014 | blepharoconjunctivitis nos |  |
| 298539019 | unspecified blepharitis |  |
| 298540017 | blepharitis nos |  |
| 298567011 | other eyelid inflammation |  |
| 298569014 | eyelid inflammation nos |  |
| 299416018 | [x]other specified inflammation of eyelid |  |
| 779311000006111 | inflammation of eyelids |  |

**ICD-10 codes**

| code | description | zoster_spec_code |
| --- | --- | --- |
| H01 | Other inflammation of eyelid |  |
| H01.0 | Blepharitis |  |
| H01.8 | Other specified inflammation of eyelid |  |
| H01.9 | Inflammation of eyelid, unspecified |  |
| H10.5 | Blepharoconjunctivitis |  |

### Conjunctivitis

**Read codes**

| medcode | readterm | zoster_spec_code |
| --- | --- | --- |
| 174 | Conjunctivitis |  |
| 1080 | Red eye NOS |  |
| 1864 | Acute conjunctivitis |  |
| 1928 | Keratoconjunctivitis sicca |  |
| 2168 | Blepharoconjunctivitis |  |
| 2398 | Chronic conjunctivitis |  |
| 3400 | Other viral conjunctivitis |  |
| 3498 | Chronic follicular conjunctivitis |  |
| 3581 | Unspecified keratoconjunctivitis |  |
| 3690 | Bacterial conjunctivitis |  |
| 6057 | Red eye symptom |  |
| 6198 | Acute follicular conjunctivitis |  |
| 7710 | Viral conjunctivitis |  |
| 10202 | Other conjunctivitis NOS |  |
| 10679 | Conjunctivitis |  |
| 12131 | Keratoconjunctivitis |  |
| 12595 | Acute mucopurulent conjunctivitis |  |
| 15083 | Unspecified acute conjunctivitis |  |
| 15102 | Unspecified chronic conjunctivitis |  |
| 15645 | Acute conjunctivitis NOS |  |
| 15686 | Other keratoconjunctivitis NOS |  |
| 16136 | [X]Viral conjunctivitis, unspecified |  |
| 16453 | Has a red eye |  |
| 17725 | Other and unspecified conjunctivitis |  |
| 19941 | Chronic conjunctivitis NOS |  |
| 20060 | Catarrhal conjunctivitis |  |
| 20305 | Other superficial keratitis without conjunctivitis NOS |  |
| 21193 | Serous conjunctivitis |  |
| 23657 | Keratoconjunctivitis sicca (excluding Sjogren's syndrome) |  |
| 24948 | Specific keratoconjunctivitis |  |
| 27324 | Viral pharyngoconjunctivitis |  |
| 27545 | Other superficial keratitis without conjunctivitis |  |
| 27546 | Herpes zoster with keratoconjunctivitis | 1 |
| 28303 | Blepharoconjunctivitis NOS |  |
| 29375 | Unspecified conjunctivitis |  |
| 29714 | Simple chronic conjunctivitis |  |
| 36188 | Pseudomembranous conjunctivitis |  |
| 38763 | Viral conjunctivitis, unspecified |  |
| 38814 | [X]Conjunctivitis in other diseases classified elsewhere |  |
| 44027 | Other keratoconjunctivitis |  |
| 44620 | Specific keratoconjunctivitis NOS |  |
| 46476 | Membranous conjunctivitis |  |
| 49802 | Neurotrophic keratoconjunctivitis |  |
| 52111 | [X]Other acute conjunctivitis |  |
| 53752 | [X]Other conjunctivitis |  |
| 72913 | [X]Other superficial keratitis without conjunctivitis |  |
| 88481 | [X]Other viral conjunctivitis |  |
| 94899 | Keratitis or keratoconjunctivitis in other exanthemata |  |
| 95894 | Conjunctivitis with mucocutaneous disorder |  |
| 101578 | [X]Keratitis+keratoconjunctivitis in other diseases CE |  |

**CPRD Aurum codes**

| medcodeid | term | zoster_spec_code |
| --- | --- | --- |
| 14550017 | simple chronic conjunctivitis |  |
| 17160012 | conjunctivitis |  |
| 66132019 | chronic follicular conjunctivitis |  |
| 68902015 | acute follicular conjunctivitis |  |
| 75462011 | viral conjunctivitis |  |
| 89308010 | acute conjunctivitis |  |
| 114036017 | blepharoconjunctivitis |  |
| 114437019 | catarrhal conjunctivitis |  |
| 119833011 | pseudomembranous conjunctivitis |  |
| 122494014 | chronic conjunctivitis |  |
| 127966019 | neurotrophic keratoconjunctivitis |  |
| 146137017 | keratoconjunctivitis |  |
| 165686018 | membranous conjunctivitis |  |
| 206640018 | bacterial conjunctivitis |  |
| 286934013 | viral pharyngoconjunctivitis |  |
| 288199014 | [x]other viral conjunctivitis |  |
| 288200012 | [x]viral conjunctivitis, unspecified |  |
| 298336010 | specific keratoconjunctivitis |  |
| 298341019 | keratoconjunctivitis sicca (excluding sjogren's syndrome) |  |
| 298343016 | specific keratoconjunctivitis nos |  |
| 298344010 | other keratoconjunctivitis |  |
| 298345011 | unspecified keratoconjunctivitis |  |
| 298346012 | keratitis or keratoconjunctivitis in other exanthemata |  |
| 298347015 | other keratoconjunctivitis nos |  |
| 298466016 | serous conjunctivitis |  |
| 298476018 | acute conjunctivitis nos |  |
| 298477010 | unspecified chronic conjunctivitis |  |
| 298483013 | chronic conjunctivitis nos |  |
| 298484019 | unspecified blepharoconjunctivitis |  |
| 298487014 | blepharoconjunctivitis nos |  |
| 298488016 | other and unspecified conjunctivitis |  |
| 298489012 | unspecified conjunctivitis |  |
| 298490015 | conjunctivitis with mucocutaneous disorder |  |
| 298492011 | other conjunctivitis nos |  |
| 299432015 | [x]other acute conjunctivitis |  |
| 299433013 | [x]other conjunctivitis |  |
| 299437014 | [x]conjunctivitis in other diseases classified elsewhere |  |
| 299443011 | [x]other superficial keratitis without conjunctivitis |  |
| 395364015 | other viral conjunctivitis |  |
| 397987018 | has a red eye |  |
| 399467019 | unspecified acute conjunctivitis |  |
| 399468012 | acute mucopurulent conjunctivitis |  |
| 444889012 | keratoconjunctivitis sicca |  |
| 1229869010 | herpes zoster with keratoconjunctivitis | 1 |
| 61121000006111 | viral conjunctivitis, unspecified |  |
| 183481000006113 | red eye symptom |  |
| 394621000006118 | [x]keratitis+keratoconjunctivitis in other diseases ce |  |
| 589531000006119 | conjunctivitis |  |
| 883431000006119 | right conjunctivitis |  |
| 883441000006112 | left conjunctivitis |  |
| 907851000006112 | [rfc] conjunctivitis |  |
| 183471000006110 | red eye nos |  |

**ICD-10 codes**

| code | description | zoster_spec_code |
| --- | --- | --- |
| B30 | Viral conjunctivitis |  |
| B30.2 | Viral pharyngoconjunctivitis |  |
| B30.8 | Other viral conjunctivitis |  |
| B30.9 | Viral conjunctivitis, unspecified |  |
| H10 | Conjunctivitis |  |
| H10.0 | Mucopurulent conjunctivitis |  |
| H10.2 | Other acute conjunctivitis |  |
| H10.3 | Acute conjunctivitis, unspecified |  |
| H10.4 | Chronic conjunctivitis |  |
| H10.5 | Blepharoconjunctivitis |  |
| H10.8 | Other conjunctivitis |  |
| H10.9 | Conjunctivitis, unspecified |  |
| H13.2 | Conjunctivitis in other diseases classified elsewhere |  |
| H16.1 | Other superficial keratitis without conjunctivitis |  |
| H16.2 | Keratoconjunctivitis |  |
| H19.1 | Herpesviral keratitis and keratoconjunctivitis |  |
| H19.3 | Keratitis and keratoconjunctivitis in other diseases classified elsewhere |  |

### Retinitis

**Read codes**

| medcode | readterm | zoster_spec_code |
| --- | --- | --- |
| 4785 | Choroiditis NOS |  |
| 6929 | Chorioretinal inflammations scars and other disorders |  |
| 10305 | Chorioretinal scars NOS |  |
| 10999 | Posterior uveitis NOS |  |
| 12249 | Chorioretinal scars |  |
| 16194 | Other chorioretinitis or retinochoroiditis NOS |  |
| 16264 | Retinitis NOS |  |
| 16629 | Retinitis and chorioretinitis |  |
| 27424 | Focal macular retinochoroiditis |  |
| 32239 | Focal chorioretinitis or retinochoroiditis NOS |  |
| 34170 | Focal chorioretinitis and retinochoroiditis |  |
| 39637 | Retinitis proliferans |  |
| 39964 | Unspecified focal chorioretinitis |  |
| 40527 | Peripheral chorioretinal scars |  |
| 43496 | Disseminated chorioretinitis and retinochoroiditis |  |
| 45908 | Focal juxtapapillary choroiditis |  |
| 48117 | Unspecified chorioretinal scar |  |
| 53869 | [X]Other chorioretinal inflammations |  |
| 55312 | Peripheral focal retinochoroiditis |  |
| 55978 | Peripheral focal chorioretinitis |  |
| 58055 | Other chorioretinitis and retinochoroiditis |  |
| 59128 | [X]Other chorioretinal disorders in diseases CE |  |
| 65993 | General disseminated chorioretinitis |  |
| 66369 | Unspecified disseminated chorioretinitis |  |
| 94848 | Other posterior pole focal chorioretinitis |  |
| 96303 | Focal juxtapapillary retinitis |  |
| 97455 | Other posterior pole focal retinitis |  |
| 99779 | Disseminated chorioretinitis and retinochoroiditis NOS |  |
| 110821 | Disseminated posterior pole chorioretinitis |  |

**CPRD Aurum codes**

| medcodeid | term | zoster_spec_code |
| --- | --- | --- |
| 297887018 | unspecified focal chorioretinitis |  |
| 297888011 | focal juxtapapillary choroiditis |  |
| 297891011 | other posterior pole focal chorioretinitis |  |
| 297893014 | other posterior pole focal retinitis |  |
| 297894015 | peripheral focal retinochoroiditis |  |
| 297895019 | focal chorioretinitis or retinochoroiditis nos |  |
| 297897010 | unspecified disseminated chorioretinitis |  |
| 297898017 | disseminated posterior pole chorioretinitis |  |
| 297899013 | disseminated peripheral chorioretinitis |  |
| 297900015 | general disseminated chorioretinitis |  |
| 297903018 | disseminated chorioretinitis and retinochoroiditis nos |  |
| 297904012 | other chorioretinitis and retinochoroiditis |  |
| 297905013 | choroiditis nos |  |
| 297906014 | retinitis nos |  |
| 297907017 | posterior uveitis nos |  |
| 297913014 | other chorioretinitis or retinochoroiditis nos |  |
| 297914015 | unspecified chorioretinal scar |  |
| 297917010 | peripheral chorioretinal scars |  |
| 297918017 | disseminated chorioretinal scars |  |
| 297919013 | chorioretinal scars nos |  |
| 299471017 | [x]other chorioretinal inflammations |  |
| 347653014 | retinitis proliferans |  |
| 1231652019 | peripheral focal chorioretinitis |  |
| 170161000006113 | retinitis and chorioretinitis |  |
| 407441000006110 | [x]other chorioretinal disorders in diseases ce |  |
| 552681000006116 | chorioretinal inflammations scars and other disorders |  |
| 552701000006118 | chorioretinal scars |  |
| 625191000006111 | disseminated chorioretinitis and retinochoroiditis |  |
| 763071000006113 | focal chorioretinitis and retinochoroiditis |  |
| 763131000006111 | focal juxtapapillary retinitis |  |
| 763141000006118 | focal macular retinochoroiditis |  |
| 931901000006118 | choroiditis |  |

**ICD-10 codes**

| code | description | zoster_spec_code |
| --- | --- | --- |
| H30 | Chorioretinal inflammation |  |
| H30.0 | Focal chorioretinal inflammation |  |
| H30.1 | Disseminated chorioretinal inflammation |  |
| H30.8 | Other chorioretinal inflammations |  |
| H30.9 | Chorioretinal inflammation, unspecified |  |
| H31.0 | Chorioretinal scars |  |
| H32 | Chorioretinal disorders in diseases classified elsewhere |  |
| H32.8 | Other chorioretinal disorders in diseases classified elsewhere |  |

### Optic neuritis

**Read codes**

| medcode | readterm | zoster_spec_code |
| --- | --- | --- |
| 3339 | Unspecified optic neuritis |  |
| 3771 | Optic neuritis |  |
| 3826 | Optic atrophy |  |
| 16228 | Unspecified optic atrophy |  |
| 20230 | Optic disc glaucomatous atrophy |  |
| 26835 | Optic neuritis NOS |  |
| 41833 | Optic atrophy NOS |  |
| 52741 | Partial optic atrophy |  |
| 59595 | Postinflammatory optic atrophy |  |

**CPRD Aurum codes**

| medcodeid | term | zoster_spec_code |
| --- | --- | --- |
| 40297018 | postinflammatory optic atrophy |  |
| 110903014 | optic neuritis |  |
| 127783019 | optic atrophy |  |
| 178771016 | partial optic atrophy |  |
| 298735011 | unspecified optic atrophy |  |
| 298744012 | optic atrophy nos |  |
| 298751015 | unspecified optic neuritis |  |
| 298753017 | optic neuritis nos |  |
| 299497011 | [x]optic atrophy in diseases classified elsewhere |  |
| 1220514012 | optic disc glaucomatous atrophy |  |

**ICD-10 codes**

| code | description | zoster_spec_code |
| --- | --- | --- |
| H46 | Optic neuritis |  |
| H47.2 | Optic atrophy |  |
| H48.0 | Optic atrophy in diseases classified elsewhere |  |

### Orbital myositis

**Read codes**

| medcode | readterm | zoster_spec_code |
| --- | --- | --- |
| 17007 | Orbital myositis |  |
| 42537 | Acute inflammation of orbit |  |
| 63561 | Acute inflammation of orbit NOS |  |
| 67505 | Unspecified chronic inflammation of orbit |  |
| 67869 | Chronic inflammation of orbit |  |
| 71969 | Chronic inflammation of orbit NOS |  |

**CPRD Aurum codes**

| medcodeid | term | zoster_spec_code |
| --- | --- | --- |
| 34588011 | acute inflammation of orbit |  |
| 74618015 | chronic inflammation of orbit |  |
| 133866014 | orbital myositis |  |
| 298681017 | acute inflammation of orbit nos |  |
| 298682012 | unspecified chronic inflammation of orbit |  |
| 298686010 | chronic inflammation of orbit nos |  |

**ICD-10 codes**

| code | description | zoster_spec_code |
| --- | --- | --- |
| H05.0 | Acute inflammation of orbit |  |
| H05.1 | Chronic inflammatory disorders of orbit |  |

### Iritis

**Read codes**

| medcode | readterm | zoster_spec_code |
| --- | --- | --- |
| 477 | Uveitis NOS |  |
| 478 | Iritis - acute |  |
| 2245 | Anterior uveitis |  |
| 2703 | Iridocyclitis |  |
| 5556 | Chronic anterior uveitis |  |
| 6849 | Acute or subacute iritis NOS |  |
| 7882 | Pars planitis |  |
| 10579 | Acute and subacute iridocyclitis |  |
| 10999 | Posterior uveitis NOS |  |
| 14731 | Unspecified iridocyclitis |  |
| 17480 | Chronic iritis |  |
| 19645 | Posterior cyclitis |  |
| 21163 | Recurrent iridocyclitis |  |
| 21685 | Panuveitis |  |
| 37140 | Chronic iridocyclitis |  |
| 38716 | Unspecified chronic iridocyclitis |  |
| 43159 | Unspecified acute iridocyclitis |  |
| 54389 | Secondary noninfected iridocyclitis |  |
| 55940 | Herpes zoster iridocyclitis | 1 |
| 63210 | Unspecified subacute iridocyclitis |  |
| 64052 | Chronic iridocyclitis due to disease EC |  |
| 64804 | Chronic iridocyclitis NOS |  |
| 69723 | Primary iridocyclitis |  |
| 72481 | Certain types of iridocyclitis |  |
| 91596 | Certain types of cyclitis NOS |  |
| 92888 | Secondary infected iridocyclitis |  |
| 98094 | Iritis |  |
| 110971 | [X]Other iridocyclitis |  |

**CPRD Aurum codes**

| medcodeid | term | zoster_spec_code |
| --- | --- | --- |
| 12361010 | recurrent iridocyclitis |  |
| 18585010 | herpes zoster iridocyclitis | 1 |
| 21698018 | primary iridocyclitis |  |
| 76191012 | pars planitis |  |
| 108146018 | iritis |  |
| 125588013 | panuveitis |  |
| 129408016 | anterior uveitis |  |
| 297907017 | posterior uveitis nos |  |
| 297958015 | unspecified acute iridocyclitis |  |
| 297959011 | unspecified subacute iridocyclitis |  |
| 297962014 | secondary infected iridocyclitis |  |
| 297965011 | acute or subacute iritis nos |  |
| 297969017 | unspecified chronic iridocyclitis |  |
| 297970016 | chronic iridocyclitis due to disease ec |  |
| 297972012 | chronic iridocyclitis nos |  |
| 297973019 | certain types of iridocyclitis |  |
| 297980017 | certain types of cyclitis nos |  |
| 299456016 | [x]other iridocyclitis |  |
| 299461019 | [x]iridocyclitis in other diseases classified elsewhere |  |
| 399440013 | acute and subacute iridocyclitis |  |
| 399442017 | unspecified iridocyclitis |  |
| 458789018 | posterior cyclitis |  |
| 1777714015 | chronic iridocyclitis |  |
| 1786145010 | chronic anterior uveitis |  |
| 2477006014 | chronic iritis |  |
| 71731000006116 | uveitis nos |  |
| 151821000006115 | secondary noninfected iridocyclitis |  |
| 745731000006116 | iritis - acute |  |
| 883281000006117 | acute iritis (iridocyclitis) |  |
| 989051000006110 | acute iritis (iridocyclitis) |  |
| 129405018 | iridocyclitis |  |

**ICD-10 codes**

| code | description | zoster_spec_code |
| --- | --- | --- |
| H20 | Iridocyclitis |  |
| H20.0 | Acute and subacute iridocyclitis |  |
| H20.1 | Chronic iridocyclitis |  |
| H20.8 | Other iridocyclitis |  |
| H20.9 | Iridocyclitis, unspecified |  |
| H22.1 | Iridocyclitis in other diseases classified elsewhere |  |
| H30.2 | Posterior cyclitis |  |

### Keratitis

**Read codes**

| medcodeid | term | zoster_spec_code |
| --- | --- | --- |
| 10808019 | keratitis |  |
| 13244016 | central corneal ulcer |  |
| 21608010 | nummular keratitis |  |
| 29014019 | diffuse interstitial keratitis |  |
| 46674018 | sclerosing keratitis |  |
| 65872016 | striate keratitis |  |
| 70919018 | punctate keratitis |  |
| 77683012 | perforated corneal ulcer |  |
| 79026014 | marginal corneal ulcer |  |
| 85429013 | filamentary keratitis |  |
| 92637018 | peripheral opacity of cornea |  |
| 94006018 | central opacity of cornea |  |
| 95157012 | bullous keratopathy |  |
| 109784013 | minor opacity of cornea |  |
| 127966019 | neurotrophic keratoconjunctivitis |  |
| 146137017 | keratoconjunctivitis |  |
| 151604014 | corneal ulcer |  |
| 178764014 | ring corneal ulcer |  |
| 298318010 | unspecified corneal ulcer |  |
| 298324016 | corneal ulcer nos |  |
| 298327011 | unspecified superficial keratitis |  |
| 298336010 | specific keratoconjunctivitis |  |
| 298341019 | keratoconjunctivitis sicca (excluding sjogren's syndrome) |  |
| 298343016 | specific keratoconjunctivitis nos |  |
| 298344010 | other keratoconjunctivitis |  |
| 298345011 | unspecified keratoconjunctivitis |  |
| 298346012 | keratitis or keratoconjunctivitis in other exanthemata |  |
| 298347015 | other keratoconjunctivitis nos |  |
| 298348013 | interstitial and deep keratitis |  |
| 298350017 | unspecified interstitial keratitis |  |
| 298356011 | interstitial and deep keratitis nos |  |
| 298357019 | unspecified corneal neovascularisation |  |
| 298362018 | corneal neovascularisation nos |  |
| 298363011 | other forms of keratitis |  |
| 298364017 | keratitis nos |  |
| 298368019 | corneal scars and opacities |  |
| 298369010 | unspecified corneal opacity |  |
| 298375018 | corneal scar or opacity nos |  |
| 299443011 | [x]other superficial keratitis without conjunctivitis |  |
| 299444017 | [x]other keratitis |  |
| 299445016 | [x]other central corneal opacity |  |
| 399462013 | corneal opacity and other disorders of cornea |  |
| 444889012 | keratoconjunctivitis sicca |  |
| 479166012 | corneal neovascularisation |  |
| 1216088016 | corneal vascularisation |  |
| 1216120011 | deep corneal vascularisation |  |
| 1229869010 | herpes zoster with keratoconjunctivitis | 1 |
| 1490262010 | localised corneal vascularisation |  |
| 155971000006110 | sclerokeratitis |  |
| 394621000006118 | [x]keratitis+keratoconjunctivitis in other diseases ce |  |
| 883401000006110 | corneal opacity/disorders nos |  |
| 883411000006113 | corneal scar/opacity |  |
| 299446015 | [x]other corneal scars and opacities |  |

**CPRD Aurum codes**

| medcodeid | term | zoster_spec_code |
| --- | --- | --- |
| 10808019 | keratitis |  |
| 13244016 | central corneal ulcer |  |
| 21608010 | nummular keratitis |  |
| 29014019 | diffuse interstitial keratitis |  |
| 46674018 | sclerosing keratitis |  |
| 65872016 | striate keratitis |  |
| 70919018 | punctate keratitis |  |
| 77683012 | perforated corneal ulcer |  |
| 79026014 | marginal corneal ulcer |  |
| 85429013 | filamentary keratitis |  |
| 92637018 | peripheral opacity of cornea |  |
| 94006018 | central opacity of cornea |  |
| 95157012 | bullous keratopathy |  |
| 109784013 | minor opacity of cornea |  |
| 127966019 | neurotrophic keratoconjunctivitis |  |
| 146137017 | keratoconjunctivitis |  |
| 151604014 | corneal ulcer |  |
| 178764014 | ring corneal ulcer |  |
| 298318010 | unspecified corneal ulcer |  |
| 298324016 | corneal ulcer nos |  |
| 298327011 | unspecified superficial keratitis |  |
| 298336010 | specific keratoconjunctivitis |  |
| 298341019 | keratoconjunctivitis sicca (excluding sjogren's syndrome) |  |
| 298343016 | specific keratoconjunctivitis nos |  |
| 298344010 | other keratoconjunctivitis |  |
| 298345011 | unspecified keratoconjunctivitis |  |
| 298346012 | keratitis or keratoconjunctivitis in other exanthemata |  |
| 298347015 | other keratoconjunctivitis nos |  |
| 298348013 | interstitial and deep keratitis |  |
| 298350017 | unspecified interstitial keratitis |  |
| 298356011 | interstitial and deep keratitis nos |  |
| 298357019 | unspecified corneal neovascularisation |  |
| 298362018 | corneal neovascularisation nos |  |
| 298363011 | other forms of keratitis |  |
| 298364017 | keratitis nos |  |
| 298368019 | corneal scars and opacities |  |
| 298369010 | unspecified corneal opacity |  |
| 298375018 | corneal scar or opacity nos |  |
| 299443011 | [x]other superficial keratitis without conjunctivitis |  |
| 299444017 | [x]other keratitis |  |
| 299445016 | [x]other central corneal opacity |  |
| 399462013 | corneal opacity and other disorders of cornea |  |
| 444889012 | keratoconjunctivitis sicca |  |
| 479166012 | corneal neovascularisation |  |
| 1216088016 | corneal vascularisation |  |
| 1216120011 | deep corneal vascularisation |  |
| 1229869010 | herpes zoster with keratoconjunctivitis | 1 |
| 1490262010 | localised corneal vascularisation |  |
| 155971000006110 | sclerokeratitis |  |
| 394621000006118 | [x]keratitis+keratoconjunctivitis in other diseases ce |  |
| 883401000006110 | corneal opacity/disorders nos |  |
| 883411000006113 | corneal scar/opacity |  |
| 299446015 | [x]other corneal scars and opacities |  |

**ICD-10 codes**

| code | description | zoster_spec_code |
| --- | --- | --- |
| H16 | Keratitis |  |
| H16.0 | Corneal ulcer |  |
| H16.1 | Other superficial keratitis without conjunctivitis |  |
| H16.2 | Keratoconjunctivitis |  |
| H16.3 | Interstitial and deep keratitis |  |
| H16.4 | Corneal neovascularization |  |
| H16.8 | Other keratitis |  |
| H16.9 | Keratitis, unspecified |  |
| H17 | Corneal scars and opacities |  |
| H17.1 | Other central corneal opacity |  |
| H17.8 | Other corneal scars and opacities |  |
| H17.9 | Corneal scar and opacity, unspecified |  |
| H18.1 | Bullous keratopathy |  |
| H19.1 | Herpesviral keratitis and keratoconjunctivitis |  |
| H19.3 | Keratitis and keratoconjunctivitis in other diseases classified elsewhere |  |

### Keratitis – surgical codes

**Read codes**

| medcode | readterm |
| --- | --- |
| 1159 | Other operation on cornea NOS |
| 1568 | Tarsorrhaphy |
| 5873 | Cornea operations |
| 5874 | [X]Corneal graft rejection |
| 9381 | [V]Cornea transplanted |
| 10930 | Botulinum toxin injection into eye muscle |
| 11545 | Other operations on cornea |
| 28412 | Other specified plastic operation on cornea |
| 29541 | Plastic operation on cornea NOS |
| 31562 | Conjunctiva and cornea operations |
| 32545 | Lateral tarsorrhaphy |
| 33822 | Other specified operations on conjunctiva or cornea |
| 33968 | Incision of cornea |
| 35908 | Plastic operations on cornea |
| 37360 | Revision of tarsorrhaphy |
| 39090 | Tarsorrhaphy NEC |
| 39136 | Amniotic membrane graft to cornea |
| 41644 | Other specified other operation on cornea |
| 48740 | Central tarsorrhaphy |
| 50511 | Conjunctiva and cornea operations NOS |
| 50955 | Medial tarsorrhaphy |
| 63057 | Incision of cornea NOS |
| 64746 | Other specified incision of cornea |
| 65744 | Tissue glue protective tarsorrhaphy |
| 90595 | Hydrogel prosthetic keratoplasty |
| 94627 | Other specified other plastic operations on cornea |
| 95004 | Other plastic operations on cornea |
| 95404 | Other plastic operations on cornea NOS |

**CPRD Aurum**

| medcodeid | term |
| --- | --- |
| 1740015 | tarsorrhaphy |
| 104596018 | incision of cornea |
| 266698016 | lateral tarsorrhaphy |
| 266699012 | medial tarsorrhaphy |
| 266700013 | tarsorrhaphy nec |
| 266701012 | revision of tarsorrhaphy |
| 266739016 | tissue glue protective tarsorrhaphy |
| 266940010 | plastic operations on cornea |
| 266943012 | other specified plastic operation on cornea |
| 266944018 | plastic operation on cornea nos |
| 266959012 | other specified incision of cornea |
| 266960019 | incision of cornea nos |
| 266966013 | other specified other operation on cornea |
| 266967016 | other operation on cornea nos |
| 266968014 | other specified operations on conjunctiva or cornea |
| 266969018 | conjunctiva and cornea operations nos |
| 347221013 | botulinum toxin injection into eye muscle |
| 394013015 | conjunctiva and cornea operations |
| 460649019 | [v]cornea transplanted |
| 501618019 | central tarsorrhaphy |
| 2675924018 | amniotic membrane graft to cornea |
| 22141000006116 | other operations on cornea |
| 375741000006114 | [x]corneal graft rejection |
| 482411000000115 | other plastic operations on cornea |
| 482471000000113 | other specified other plastic operations on cornea |
| 482491000000112 | other plastic operations on cornea nos |
| 482751000000110 | hydrogel prosthetic keratoplasty |
| 595111000006119 | cornea operations |
| 859361000006110 | corneal operations nos |
| 859371000006115 | corneal operations |
| 859491000006112 | corneal transplant |
| 859501000006116 | other keratoplasty |
| 932091000006115 | therapeut inject botulin toxin |
| 933321000006111 | corneal transplant |
| 988021000006110 | corneal operations nos |

**OPCS codes**

| opcscode | opcsterm |
| --- | --- |
| C16.1 | Central tarsorrhaphy |
| C16.2 | Lateral tarsorrhaphy |
| C16.3 | Medial tarsorrhaphy |
| C16.4 | Tarsorrhaphy NEC |
| C16.5 | Revision of tarsorrhaphy |
| C44 | Other plastic operations on cornea |
| C44.1 | Hydrogel prosthetic keratoplasty |
| C44.8 | Other specified other plastic operations on cornea |
| C44.9 | Unspecified other plastic operations on cornea |
| C46 | Plastic operations on cornea |
| C46.6 | Amniotic membrane graft to cornea |
| C46.8 | Other specified plastic operations on cornea |
| C46.9 | Unspecified plastic operations on cornea |
| C49 | Incision of cornea |
| C49.8 | Other specified incision of cornea |
| C49.9 | Unspecified incision of cornea |
| C51 | Other operations on cornea |
| C51.8 | Other specified other operations on cornea |
| C51.9 | Unspecified other operations on cornea |

### Episcleritis/ Scleritis

**Read codes**

| medcode | readterm | zoster_spec_code |
| --- | --- | --- |
| 136 | Episcleritis |  |
| 5318 | Scleritis |  |
| 6783 | Nodular episcleritis |  |
| 15996 | Scleritis and episcleritis |  |
| 28123 | Posterior scleritis |  |
| 35599 | Unspecified scleritis |  |
| 40365 | Anterior scleritis |  |
| 44859 | Scleritis or episcleritis NOS |  |
| 101293 | [X]Scleritis+episcleritis in diseases CE |  |

**CPRD Aurum codes**

| medcodeid | term | zoster_spec_code |
| --- | --- | --- |
| 2432010 | episcleritis |  |
| 105477014 | anterior scleritis |  |
| 117201018 | nodular episcleritis |  |
| 130057013 | scleritis |  |
| 298859012 | unspecified scleritis |  |
| 298867016 | scleritis or episcleritis nos |  |
| 399488011 | scleritis and episcleritis |  |
| 399489015 | posterior scleritis |  |
| 425741000006117 | [x]scleritis+episcleritis in diseases ce |  |
| 883591000006116 | scleritis/episcleritis |  |

**ICD-10 codes**

| code | description | zoster_spec_code |
| --- | --- | --- |
| H15.0 | Scleritis |  |
| H15.1 | Episcleritis |  |
| H19.0 | Scleritis and episcleritis in diseases classified elsewhere |  |

### Glaucoma - diagnoses

**Read codes**

| medcode | readterm | zoster_spec_code |
| --- | --- | --- |
| 1611 | Ocular hypertension |  |
| 1798 | Open-angle glaucoma |  |
| 2074 | Glaucoma |  |
| 2399 | Glaucoma monitoring |  |
| 8001 | Glaucoma NOS |  |
| 20230 | Optic disc glaucomatous atrophy |  |
| 22805 | Glaucoma due to unspecified ocular disorder |  |
| 22904 | Referral to glaucoma clinic |  |
| 26870 | Glaucoma due to ocular inflammation |  |
| 28189 | Open-angle glaucoma NOS |  |
| 28505 | Other specified forms of glaucoma |  |
| 29764 | Glaucomatocyclitic crises |  |
| 35528 | Steroid-induced glaucoma |  |
| 41804 | Glaucoma due to disease NOS |  |
| 41854 | Glaucoma due to disease EC |  |
| 42447 | Unspecified open-angle glaucoma |  |
| 44295 | Other specified glaucoma NOS |  |
| 44338 | Glaucoma - absolute |  |
| 48132 | Steroid-induced glaucoma NOS |  |
| 52888 | [X]Glaucoma |  |
| 53127 | Glaucoma associated with other ocular disorders |  |
| 53521 | Glaucoma associated with other ocular disorders NOS |  |
| 68094 | Steroid-induced glaucoma residual stage |  |
| 70195 | [X]Other glaucoma |  |
| 72394 | Open-angle glaucoma residual stage |  |

**CPRD Aurum codes**

| medcodeid | term | zoster_spec_code |
| --- | --- | --- |
| 8093016 | ocular hypertension |  |
| 40268016 | glaucoma |  |
| 140102012 | open-angle glaucoma |  |
| 264949013 | glaucoma monitoring |  |
| 298034019 | unspecified open-angle glaucoma |  |
| 298042018 | open-angle glaucoma nos |  |
| 298050010 | steroid-induced glaucoma glaucomatous stage |  |
| 298051014 | steroid-induced glaucoma residual stage |  |
| 298052019 | steroid-induced glaucoma nos |  |
| 298053012 | glaucoma due to disease ec |  |
| 298059011 | glaucoma due to disease nos |  |
| 298061019 | glaucoma associated with other ocular disorders |  |
| 298062014 | glaucoma due to unspecified ocular disorder |  |
| 298071017 | glaucoma associated with other ocular disorders nos |  |
| 298072012 | other specified forms of glaucoma |  |
| 298074013 | other specified glaucoma nos |  |
| 298075014 | glaucoma nos |  |
| 299482013 | [x]other glaucoma |  |
| 299484014 | [x]glaucoma in other diseases classified elsewhere |  |
| 1220514012 | optic disc glaucomatous atrophy |  |
| 1221368012 | steroid-induced glaucoma |  |
| 1226368013 | glaucomatocyclitic crises |  |
| 2533408017 | referral to glaucoma clinic |  |
| 47081000006113 | open-angle glaucoma residual stage |  |
| 388091000006115 | [x]glaucoma |  |
| 556201000006114 | chronic primary angle-closure glaucoma |  |
| 802901000006112 | glaucoma - absolute |  |
| 803011000006118 | glaucoma due to ocular inflammation |  |
| 856591000006115 | glaucoma right eye |  |
| 856601000006111 | glaucoma left eye |  |
| 883301000006118 | secondary/other glaucoma |  |
| 907841000006110 | [rfc] glaucoma |  |

**ICD-10 codes**

| code | description | zoster_spec_code |
| --- | --- | --- |
| H40 | Glaucoma |  |
| H40.0 | Glaucoma suspect |  |
| H40.5 | Glaucoma secondary to other eye disorders |  |
| H40.8 | Other glaucoma |  |
| H40.9 | Glaucoma, unspecified |  |
| H42 | Glaucoma in diseases classified elsewhere |  |
| H42.8 | Glaucoma in other diseases classified elsewhere |  |

### Glaucoma – surgical codes

**Read codes**

| medcode | readterm |
| --- | --- |
| 2077 | Trabeculectomy |
| 2824 | Incision of iris |
| 3577 | Iridectomy NEC |
| 5685 | Laser trabeculoplasty |
| 6590 | Laser iridotomy |
| 10540 | Iridoplasty NEC |
| 10557 | Surgical iridotomy |
| 10987 | Trabeculotomy |
| 11117 | Iridotomy NEC |
| 15591 | Surgical iridectomy |
| 15694 | Incision of iris NOS |
| 18343 | Irrigation of anterior chamber of eye |
| 20236 | Other specified filtering operation on iris |
| 30212 | Revision of trabeculectomy |
| 30528 | Transcleral diode laser cycloablation |
| 31361 | Filtering operations on iris |
| 35768 | Filtering operation on iris NOS |
| 40076 | Goniotomy |
| 40987 | Trephining of sclera and iridectomy |
| 40989 | Insertion Molteno implantation tube in anterior chamber eye |
| 41836 | Washout of anterior chamber of eye |
| 42778 | Viscocanulostomy |
| 47221 | Elliot trephining of sclera and iridectomy |
| 51128 | Resuturing of trabeculectomy |
| 51243 | Iridocyclectomy |
| 54469 | Other specified operations on sclera or iris |
| 68013 | Viscocanalostomy |
| 83561 | Deep sclerectomy without spacer |
| 92300 | Other specified incision of iris |
| 95423 | Deep sclerectomy with spacer |

**CPRD Aurum**

| medcodeid | term |
| --- | --- |
| 34312019 | iridocyclectomy |
| 82109011 | irrigation of anterior chamber of eye |
| 137251018 | trabeculotomy |
| 267035010 | laser trabeculoplasty |
| 267053016 | other specified incision of iris |
| 267054010 | incision of iris nos |
| 347247016 | iridotomy nec |
| 347248014 | iridectomy nec |
| 394017019 | surgical iridectomy |
| 394018012 | iridoplasty nec |
| 394020010 | goniotomy |
| 394022019 | surgical iridotomy |
| 456790015 | viscocanulostomy |
| 458411014 | revision of trabeculectomy |
| 458430014 | resuturing of trabeculectomy |
| 484355015 | trephining of sclera and iridectomy |
| 484726014 | trabeculectomy |
| 1208748016 | viscocanalostomy |
| 1210015016 | laser iridotomy |
| 1480749010 | iridotomy |
| 1491630011 | incision of iris |
| 2474291014 | transcleral diode laser cycloablation |
| 2675997018 | deep sclerectomy with spacer |
| 2675998011 | deep sclerectomy without spacer |
| 6981000006116 | other specified operations on sclera or iris |
| 11311000006118 | other specified filtering operation on iris |
| 58381000006114 | washout of anterior chamber of eye |
| 638641000006119 | elliot trephining of sclera and iridectomy |
| 709911000006117 | filtering operation on iris nos |
| 709921000006113 | filtering operations on iris |
| 772991000006113 | insertion molteno implantation tube in anterior chamber eye |

**OPCS codes**

| opcscode | opcsterm |
| --- | --- |
| C52.1 | Deep sclerectomy with spacer |
| C52.2 | Deep sclerectomy without spacer |
| C59.1 | Iridocyclectomy |
| C59.2 | Surgical iridectomy |
| C60 | Filtering operations on iris |
| C60.1 | Trabeculectomy |
| C60.4 | Iridoplasty NEC |
| C60.5 | Insertion of tube into anterior chamber of eye to assist drainage of aqueous humour |
| C60.6 | Viscocanulostomy |
| C60.8 | Other specified filtering operations on iris |
| C60.9 | Unspecified filtering operations on iris |
| C61.1 | Laser trabeculoplasty |
| C61.2 | Trabeculotomy |
| C61.3 | Goniotomy |
| C62 | Incision of iris |
| C62.2 | Surgical iridotomy |
| C62.3 | Laser iridotomy |
| C62.8 | Other specified incision of iris |
| C62.9 | Unspecified incision of iris |
| C69.4 | Irrigation of anterior chamber of eye |

### Vision loss/Blindness

**Read codes**

| medcode | readterm | zoster_spec_code |
| --- | --- | --- |
| 746 | Blurred vision NOS |  |
| 1990 | Blindness, both eyes |  |
| 3268 | Loss of vision |  |
| 3553 | Double vision |  |
| 3839 | O/E - has one eye |  |
| 3851 | Blindness, one eye, unspecified |  |
| 3852 | O/E - blind L-eye |  |
| 3976 | Partial sight |  |
| 4147 | Other binocular vision disorders |  |
| 5991 | Diplopia/double vision |  |
| 6020 | Impaired vision |  |
| 6661 | [V]Problems with sight |  |
| 7203 | Low vision, both eyes |  |
| 8186 | Deteriorating vision |  |
| 8367 | Blindness and low vision |  |
| 9645 | Sight impaired |  |
| 10388 | O/E - blind R-eye |  |
| 10868 | Blind hypotensive eye |  |
| 12493 | Low vision |  |
| 15269 | Profound impairment, one eye |  |
| 16125 | Blind spot scotoma |  |
| 18385 | Patient concerned about eyesight |  |
| 18462 | Diplopia (double vision) |  |
| 20607 | Low vision, one eye, unspecified |  |
| 21748 | Cloudy vision NOS |  |
| 23742 | Acquired blindness |  |
| 25547 | Visual loss, one eye, unqualified |  |
| 25831 | Low vision, one eye |  |
| 29387 | One eye blind, one eye low vision |  |
| 33014 | O/E - R-eye completely blind |  |
| 33016 | O/E - L-eye completely blind |  |
| 33447 | Low vision, both eyes NOS |  |
| 37086 | Blind hypertensive eye |  |
| 37146 | Unspecified binocular vision disorder |  |
| 37893 | Low vision, one eye NOS |  |
| 38000 | Acquired blindness, one eye |  |
| 40451 | Low vision, both eyes unspecified |  |
| 40955 | Dull vision NOS |  |
| 42262 | Acquired blindness, both eyes |  |
| 44954 | Profound impairment one eye NOS |  |
| 47956 | Blindness both eyes NOS |  |
| 51274 | O/E - pinhole R-eye completely blind |  |
| 54513 | O/E - pinhole L-eye completely blind |  |
| 54562 | Other binocular vision disturbance NOS |  |
| 54962 | One eye blind, one eye low vision NOS |  |
| 55108 | Unspecified blindness both eyes |  |
| 55188 | Blind spot area scotoma NOS |  |
| 55846 | Blind rehabilitation |  |
| 59214 | Binocular vision suppression |  |
| 67126 | Blind rehabilitation |  |
| 73508 | Blind telephone user |  |
| 98637 | [X]Visual disturbances and blindness |  |
| 104077 | Blindness, monocular |  |
| 111782 | [X]Other disorders of binocular vision |  |

**CPRD Aurum codes**

| medcodeid | term | zoster_spec_code |
| --- | --- | --- |
| 1529013 | blind hypertensive eye |  |
| 14191011 | impaired vision |  |
| 41871011 | double vision |  |
| 255275013 | o/e - blind r-eye |  |
| 255276014 | o/e - r-eye completely blind |  |
| 255292016 | o/e - blind l-eye |  |
| 255293014 | o/e - l-eye completely blind |  |
| 283416014 | blind rehabilitation |  |
| 298183018 | other binocular vision disorders |  |
| 298190011 | other binocular vision disturbance nos |  |
| 298199012 | blind spot scotoma |  |
| 298203012 | blind spot area scotoma nos |  |
| 298252011 | unspecified blindness both eyes |  |
| 298262016 | blindness both eyes nos |  |
| 298264015 | one eye blind, one eye low vision |  |
| 298276015 | one eye blind, one eye low vision nos |  |
| 298277012 | low vision, both eyes |  |
| 298278019 | low vision, both eyes unspecified |  |
| 298284016 | low vision, both eyes nos |  |
| 298287011 | profound impairment, one eye |  |
| 298288018 | blindness, one eye, unspecified |  |
| 298299016 | profound impairment one eye nos |  |
| 298300012 | low vision, one eye |  |
| 298301011 | low vision, one eye, unspecified |  |
| 298308017 | low vision, one eye nos |  |
| 298309013 | visual loss, one eye, unqualified |  |
| 299506011 | [x]visual disturbances and blindness |  |
| 299507019 | [x]other disorders of binocular vision |  |
| 338995019 | deteriorating vision |  |
| 347842017 | acquired blindness |  |
| 399453011 | blurred vision nos |  |
| 456798010 | acquired blindness, both eyes |  |
| 456799019 | acquired blindness, one eye |  |
| 460633016 | [v]problems with sight |  |
| 493267010 | blind hypotensive eye |  |
| 1780217018 | patient concerned about eyesight |  |
| 2163958019 | o/e - pinhole l-eye completely blind |  |
| 2163962013 | o/e - pinhole r-eye completely blind |  |
| 2164154014 | low vision |  |
| 2164155010 | sight impaired |  |
| 2164158012 | partial sight |  |
| 515731000006113 | blind rehabilitation |  |
| 515791000006112 | blindness and low vision |  |
| 515811000006111 | blindness, both eyes |  |
| 570481000006117 | cloudy vision nos |  |
| 622011000006112 | diplopia (double vision) |  |
| 622021000006116 | diplopia/double vision |  |
| 631921000006112 | dull vision nos |  |
| 734661000006116 | loss of vision |  |
| 855291000006110 | blind (subjectively) |  |
| 856171000006114 | partially sighted (subjectively) |  |
| 883331000006114 | diplopia - double vision |  |
| 883361000006117 | blind/low vision - both eyes |  |
| 883371000006112 | blind - both eyes |  |
| 883381000006110 | blind/low vision -one eye only |  |
| 883391000006113 | blindness/low vision nos |  |
| 907781000006114 | [rfc] sudden onset squints/double vision |  |
| 907831000006117 | [rfc] advice/care for the blind |  |
| 915221000006114 | patient concerned about eyesight |  |
| 982291000006118 | sight impairment |  |
| 988991000006111 | blind/low vision - both eyes |  |
| 989001000006111 | blind/low vision -one eye only |  |
| 1809401000006114 | vision change |  |
| 1816041000006111 | blindness, monocular |  |
| 2267571000000111 | deafblind |  |
| 298184012 | unspecified binocular vision disorder |  |

**ICD-10 codes**

| code | description | zoster_spec_code |
| --- | --- | --- |
| H53.3 | Other disorders of binocular vision |  |
| H54 | Visual impairment including blindness (binocular or monocular) |  |
| H54.0 | Blindness, binocular |  |
| H54.4 | Blindness, monocular |  |

### Non-specific eye infection

**Read codes**

| medcode | readterm | zoster_spec_code |
| --- | --- | --- |
| 3790 | Eye infection |  |
| 9176 | Eye infection |  |
| 53384 | Other eyelid infective dermatitis |  |
| 63904 | Infective eyelid dermatitis of types resulting in deformity |  |
| 111438 | [X]Involvement of eyelid in other infectious diseases CE |  |

**CPRD Aurum codes**

| medcodeid | term | zoster_spec_code |
| --- | --- | --- |
| 206641019 | eye infection |  |
| 298564016 | other eyelid infective dermatitis |  |
| 394481000006117 | [x]involvement of eyelid in other infectious diseases ce |  |
| 660601000006111 | eye infection |  |
| 779631000006110 | infective eyelid dermatitis of types resulting in deformity |  |

**ICD-10 codes**

| code | description | zoster_spec_code |
| --- | --- | --- |
| H03.1 | Involvement of eyelid in other infectious diseases classified elsewhere |  |

### Eye treatments (antivirals/antibacterials)

| prodcode | av | productname |
| --- | --- | --- |
| 2392 | 1 | aciclovir 3% eye ointment |
| 19660 | 1 | aciclovir eye |
| 11825 | 1 | ganciclovir 0.15% eye gel |
| 10437 | 1 | idoxene 0.50% eye ointment (spodefell ltd) |
| 26319 | 1 | idoxene eye |
| 10826 | 1 | idoxuridine 0.50% eye ointment |
| 22508 | 1 | idoxuridine drops .5 % eye |
| 10503 | 1 | idoxuridine oint eye |
| 17777 | 1 | idoxuridine with polyvinyl alcohol ophthalmic solution |
| 12476 | 1 | kerecid eye drops (allergan ltd) |
| 59558 | 1 | trifluridine 1% eye drops |
| 41153 | 1 | virgan 0.15% eye gel (spectrum thea pharmaceuticals ltd) |
| 17518 | 1 | virgan 0.15% eye gel (thea pharmaceuticals ltd) |
| 57553 | 1 | zovirax 3% ophthalmic ointment (dowelhurst ltd) |
| 2509 | 1 | zovirax 3% ophthalmic ointment (glaxosmithkline uk ltd) |
| 67243 | 1 | zovirax 3% ophthalmic ointment (lexon (uk) ltd) |
| 45744 |  | achromycin 1% eye drops (wyeth pharmaceuticals) |
| 1479 |  | achromycin 1% eye ointment (wyeth pharmaceuticals) |
| 21600 |  | achromycin eye |
| 7847 |  | achromycin eye 1 % dro |
| 12027 |  | albucid drops 20 % eye |
| 11998 |  | albucid drops 30 % eye |
| 22299 |  | albucid eye |
| 23188 |  | albucid eye |
| 12006 |  | albucid oint 6 % eye |
| 1578 |  | aureomycin 1% eye ointment (wyeth pharmaceuticals) |
| 4567 |  | aureomycin 3% cream (wyeth pharmaceuticals) |
| 7679 |  | aureomycin 3% ointment (wyeth pharmaceuticals) |
| 20678 |  | aureomycin eye |
| 46603 |  | azithromycin 15mg/g eye drops 0.25g unit dose preservative free |
| 46994 |  | azyter 15mg/g eye drops 0.25g unit dose (thea pharmaceuticals ltd) |
| 13269 |  | betamethasone 0.1% / neomycin 0.5% ear/eye/nose drops |
| 4164 |  | betamethasone sodium phosphate 0.1% with neomycin 0.5% eye drops |
| 34628 |  | betamethasone with neomycin eye drops (c p pharmaceuticals ltd) |
| 48697 |  | blephasol lotion (thea pharmaceuticals ltd) |
| 35930 |  | brochlor 0.5% eye drops (sanofi) |
| 37159 |  | brochlor 1% eye ointment (sanofi) |
| 70562 |  | brolene antibiotic 0.5% eye drops (sanofi) |
| 33076 |  | cefotoxamine drops eye |
| 68106 |  | ceftazidime 5% eye drops preservative free |
| 41185 |  | cefuroxime 5% eye drops |
| 46402 |  | cefuroxime 5% eye drops preservative free |
| 61860 |  | cefuroxime 50mg powder for solution for injection vials |
| 79 |  | chloramphenicol 0.5% eye drops |
| 34368 |  | chloramphenicol 0.5% eye drops (a a h pharmaceuticals ltd) |
| 34764 |  | chloramphenicol 0.5% eye drops (actavis uk ltd) |
| 57925 |  | chloramphenicol 0.5% eye drops (alliance healthcare (distribution) ltd) |
| 66626 |  | chloramphenicol 0.5% eye drops (almus pharmaceuticals ltd) |
| 52272 |  | chloramphenicol 0.5% eye drops (amco) |
| 34462 |  | chloramphenicol 0.5% eye drops (arrow generics ltd) |
| 62489 |  | chloramphenicol 0.5% eye drops (kent pharmaceuticals ltd) |
| 24004 |  | chloramphenicol 0.5% eye drops (martindale pharmaceuticals ltd) |
| 66647 |  | chloramphenicol 0.5% eye drops (phoenix healthcare distribution ltd) |
| 54601 |  | chloramphenicol 0.5% eye drops (sigma pharmaceuticals plc) |
| 34467 |  | chloramphenicol 0.5% eye drops (teva uk ltd) |
| 58648 |  | chloramphenicol 0.5% eye drops (vantage) |
| 60077 |  | chloramphenicol 0.5% eye drops (waymade healthcare plc) |
| 5566 |  | chloramphenicol 0.5% eye drops 0.5ml unit dose preservative free |
| 68707 |  | chloramphenicol 0.5% eye drops preservative free |
| 433 |  | chloramphenicol 0.5% viscous eye drops |
| 34971 |  | chloramphenicol 0.50% eye drops (c p pharmaceuticals ltd) |
| 47787 |  | chloramphenicol 0.50% eye drops (celltech pharma europe ltd) |
| 34623 |  | chloramphenicol 0.50% eye drops (co-pharma ltd) |
| 29338 |  | chloramphenicol 0.50% eye drops (schering-plough ltd) |
| 41518 |  | chloramphenicol 0.50% eye drops (teva uk ltd) |
| 170 |  | chloramphenicol 1% eye ointment |
| 19177 |  | chloramphenicol 1% eye ointment (a a h pharmaceuticals ltd) |
| 34510 |  | chloramphenicol 1% eye ointment (actavis uk ltd) |
| 61284 |  | chloramphenicol 1% eye ointment (alliance healthcare (distribution) ltd) |
| 55818 |  | chloramphenicol 1% eye ointment (almus pharmaceuticals ltd) |
| 59097 |  | chloramphenicol 1% eye ointment (amco) |
| 42899 |  | chloramphenicol 1% eye ointment (arrow generics ltd) |
| 34688 |  | chloramphenicol 1% eye ointment (co-pharma ltd) |
| 65941 |  | chloramphenicol 1% eye ointment (de pharmaceuticals) |
| 33246 |  | chloramphenicol 1% eye ointment (ivax pharmaceuticals uk ltd) |
| 68202 |  | chloramphenicol 1% eye ointment (kent pharmaceuticals ltd) |
| 30766 |  | chloramphenicol 1% eye ointment (martindale pharmaceuticals ltd) |
| 58666 |  | chloramphenicol 1% eye ointment (phoenix healthcare distribution ltd) |
| 41545 |  | chloramphenicol 1% eye ointment (schering-plough ltd) |
| 23239 |  | chloramphenicol 1% eye ointment (teva uk ltd) |
| 49651 |  | chloramphenicol 1% eye ointment (vantage) |
| 56878 |  | chloramphenicol 1% eye ointment (waymade healthcare plc) |
| 22300 |  | chloramphenicol 1%/hydrocortis.0.5% eye |
| 48296 |  | chloramphenicol antibiotic 0.5% eye drops (numark ltd) |
| 19572 |  | chloramphenicol eye |
| 19713 |  | chloramphenicol eye |
| 34126 |  | chloramphenicol eye ointment (thornton & ross ltd) |
| 2877 |  | chloramphenicol oint eye |
| 1777 |  | chloramphenicol with hydrocortisone eye ointment |
| 3543 |  | chloramphenicol&hydrocortisone oint eye |
| 23500 |  | chloramphenicol/polyvinyl alcohol eye |
| 7958 |  | chloramphenicol/polyvinyl alcohol eye dro |
| 1769 |  | chloromycetin 1% eye ointment (amco) |
| 19515 |  | chloromycetin eye |
| 19517 |  | chloromycetin eye |
| 21650 |  | chloromycetin hydrocortisone eye |
| 2076 |  | chloromycetin redidrops 0.5% (amco) |
| 2338 |  | chloromycetin with hydrocortisone 1%+0.5% eye ointment (parke-davis research laboratories) |
| 293 |  | chlortetracycline 1% eye ointment |
| 7596 |  | chlortetracycline 1% eye ointment |
| 54482 |  | chlortetracycline 1% eye ointment |
| 27473 |  | chlortetracycline hydrochloride eye |
| 2813 |  | chlortetraycline 3% cream |
| 27032 |  | cidomycin ear/eye |
| 3573 |  | cidomycin ear/eye dro |
| 4568 |  | cidomycin eye 3 % oin |
| 20083 |  | cidomycin eye drops (hoechst marion roussel) |
| 26882 |  | cidomycin eye ointment (hoechst marion roussel) |
| 5631 |  | ciloxan 0.3% eye drops (novartis pharmaceuticals uk ltd) |
| 19512 |  | ciloxan 3mg/g eye ointment (alcon laboratories (uk) ltd) |
| 50141 |  | ciprofloxacin 0.2% eye drops preservative free |
| 528 |  | ciprofloxacin 0.3% eye drops |
| 14233 |  | ciprofloxacin 3mg/g eye ointment |
| 29569 |  | clobetasone 0.1%/neomycin 0.5% eye |
| 17743 |  | clobetasone butyrate with neomycin eye drops |
| 11628 |  | dexamethasone 0.1% / tobramycin 0.3% eye drops |
| 7360 |  | dexamethasone with framycetin with gramicidin ear/eye drops |
| 13257 |  | dexamethasone with framycetin with gramicidin eye drops |
| 14665 |  | dexamethasone with framycetin with gramicidin eye ointment |
| 5006 |  | dexamethasone with neomycin with polymyxin eye drops |
| 14051 |  | dexamethasone with neomycin with polymyxin eye ointment |
| 58487 |  | erythromycin 0.5% eye ointment |
| 4069 |  | exocin 0.3% eye drops (allergan ltd) |
| 66824 |  | exocin 0.3% eye drops (mawdsley-brooks & company ltd) |
| 19716 |  | fluorometholone 0.1%/neomycin 0.5% eye % dro |
| 24876 |  | fluorometholone with neomycin eye drops |
| 2939 |  | framycetin 0.5% eye drops |
| 411 |  | framycetin 0.5% eye ointment |
| 27902 |  | framycetin 0.5%/hydrocort. 0.5% ear/eye |
| 27864 |  | framycetin 0.5%/hydrocortisone 0.5% eye |
| 21597 |  | framycetin sulphate eye |
| 22102 |  | framycetin sulphate eye |
| 27073 |  | framycetin with dexamethasone and gramicidin ear/eye drops solution |
| 14565 |  | framycetin with dexamethasone and gramicidin eye drops |
| 14606 |  | framycetin with dexamethasone and gramicidin eye gel |
| 17374 |  | framycetin with dexamethasone and gramicidin eye gel |
| 12267 |  | framycetin with hydrocortisone eye drops |
| 17839 |  | framycetin with hydrocortisone eye ointment |
| 19634 |  | framycort ear/eye |
| 27533 |  | framycort eye |
| 2692 |  | framycort eye drops (rpr / fisons) |
| 13480 |  | framycort eye ointment (rpr / fisons) |
| 23658 |  | framygen eye |
| 10450 |  | framygen eye drops (rpr / fisons) |
| 13339 |  | framygen eye ointment (rpr / fisons) |
| 63597 |  | fucithalmic (roi) 1% eye drops (leo pharma) |
| 1078 |  | fucithalmic 1% eye drops (amco) |
| 62631 |  | fucithalmic 1% eye drops (dowelhurst ltd) |
| 3171 |  | fusidic acid 1% modified-release eye drops |
| 61382 |  | fusidic acid 1% modified-release eye drops (a a h pharmaceuticals ltd) |
| 63373 |  | fusidic acid 1% modified-release eye drops (alliance healthcare (distribution) ltd) |
| 61771 |  | fusidic acid 1% modified-release eye drops (amco) |
| 66625 |  | fusidic acid 1% modified-release eye drops (waymade healthcare plc) |
| 53386 |  | fusidic acid 1% unit dose eye drops |
| 62444 |  | garamycin 0.3% eye/ear drops (schering-plough ltd) |
| 21881 |  | garamycin ear/eye |
| 3889 |  | garamycin eye drops (schering-plough ltd) |
| 27858 |  | garamycin oint eye |
| 56225 |  | generic sofradex ear/eye drops |
| 14320 |  | gentamicin 0.3% ear/eye drops |
| 66909 |  | gentamicin 0.3% ear/eye drops (a a h pharmaceuticals ltd) |
| 64228 |  | gentamicin 0.3% ear/eye drops (alliance healthcare (distribution) ltd) |
| 64421 |  | gentamicin 0.3% ear/eye drops (amco) |
| 70925 |  | gentamicin 0.3% ear/eye drops (de pharmaceuticals) |
| 65612 |  | gentamicin 0.3% ear/eye drops (mawdsley-brooks & company ltd) |
| 1129 |  | gentamicin 0.3% eye drops |
| 11503 |  | gentamicin 0.3% eye drops 0.5ml unit dose preservative free |
| 1690 |  | gentamicin 0.3% eye ointment |
| 47385 |  | gentamicin 1.5% eye drops |
| 70110 |  | gentamicin 1.5% eye drops |
| 51662 |  | gentamicin 1.5% eye drops preservative free |
| 19650 |  | gentamicin ear/eye |
| 19759 |  | gentamicin eye |
| 22175 |  | gentamicin eye |
| 47619 |  | gentamicin eye drops |
| 40471 |  | genticin 0.3% eye/ear drops (amco) |
| 71319 |  | genticin 0.3% eye/ear drops (sigma pharmaceuticals plc) |
| 67260 |  | genticin 0.3% eye/ear drops (waymade healthcare plc) |
| 13275 |  | genticin 0.30% ear/eye drops solution (roche products ltd) |
| 19527 |  | genticin ear/eye |
| 19679 |  | genticin eye |
| 1494 |  | genticin eye drops (roche products ltd) |
| 2477 |  | genticin eye ointment (roche products ltd) |
| 36843 |  | golden eye antibiotic 0.5% eye drops (cambridge healthcare supplies ltd) |
| 38947 |  | golden eye antibiotic 1% eye ointment (cambridge healthcare supplies ltd) |
| 25933 |  | golden oint eye |
| 30546 |  | gramicidin 0.025%/neomycin 0.25% eye |
| 10821 |  | gramicidin 0.025%/neomycin 0.25% eye oin |
| 32826 |  | gramicidin/neomycin/polymixin eye 25 i/u dro |
| 12159 |  | graneodin eye ointment (e r squibb and sons ltd) |
| 21495 |  | hydrocortisone & neomycin drops 1 % eye |
| 57459 |  | hydrocortisone 1.5% / neomycin 0.5% ear/eye ointment |
| 22481 |  | hydrocortisone 1.5%/neomycin 0.5% ear/ey |
| 20583 |  | hydrocortisone with neomycin 1%+0.5% eye drops |
| 33941 |  | levofloxacin 5mg/ml eye drops |
| 68576 |  | levofloxacin 5mg/ml eye drops 0.3ml unit dose preservative free |
| 38239 |  | levofloxacin 5mg/ml eye drops 0.5ml unit dose preservative free |
| 22925 |  | lomefloxacin hc 3mg/ml eye drops |
| 1895 |  | maxitrol eye drops (novartis pharmaceuticals uk ltd) |
| 7032 |  | maxitrol eye ointment (novartis pharmaceuticals uk ltd) |
| 4363 |  | minims chloramphenicol 0.5% eye drops 0.5ml unit dose (bausch & lomb uk ltd) |
| 24229 |  | minims chloramphenicol eye drops |
| 16513 |  | minims gentamicin sulfate 0.3% eye drops 0.5ml unit dose (bausch & lomb uk ltd) |
| 18951 |  | minims neomycin sulphate 0.50% eye drops (chauvin pharmaceuticals ltd) |
| 45987 |  | moxifloxacin 0.5% eye drops |
| 54585 |  | moxivig 0.5% eye drops (novartis pharmaceuticals uk ltd) |
| 21596 |  | neo-cortef ear/eye |
| 22247 |  | neo-cortef ear/eye |
| 52083 |  | neo-cortef ear/eye drops (pliva pharma ltd) |
| 30228 |  | neo-cortef ear/eye drops solution (dominion pharma) |
| 34147 |  | neo-cortef ear/eye drops solution (dominion pharma) |
| 51684 |  | neo-cortef ear/eye ointment (pliva pharma ltd) |
| 3292 |  | neo-cortef eye drops (dominion pharma) |
| 14811 |  | neo-cortef eye drops (pliva pharma ltd) |
| 12723 |  | neo-cortef eye ointment (dominion pharma) |
| 15282 |  | neo-cortef eye ointment (pliva pharma ltd) |
| 27000 |  | neomycin & bacitracin oint eye |
| 7529 |  | neomycin 0.5% eye drops |
| 71058 |  | neomycin 0.5% eye drops (alliance healthcare (distribution) ltd) |
| 34108 |  | neomycin 0.5% eye drops (martindale pharmaceuticals ltd) |
| 26936 |  | neomycin 0.5% eye drops 0.5ml unit dose preservative free |
| 3251 |  | neomycin 0.5% eye ointment |
| 38442 |  | neomycin 0.5% eye ointment (martindale pharmaceuticals ltd) |
| 30110 |  | neomycin 0.5%/prednisolone 0.5% ear/eye |
| 8894 |  | neomycin 0.5%/prednisolone 0.5% ear/eye % dro |
| 8210 |  | neomycin drops .25 % eye |
| 34118 |  | neomycin eye drops (c p pharmaceuticals ltd) |
| 34116 |  | neomycin eye ointment (c p pharmaceuticals ltd) |
| 10371 |  | neomycin oint .5 % eye |
| 10440 |  | neomycin sulphate .5 % eye |
| 27867 |  | neomycin sulphate eye |
| 11434 |  | neomycin sulphate with betamethasone sodium phosphate 0.5% + 0.1% eye drops |
| 21591 |  | neomycin sulphate with betamethasone sodium phosphate 0.5% + 0.1% eye drops |
| 25785 |  | neomycin sulphate with betamethasone sodium phosphate 0.5% + 0.1% eye/ear/nose drops |
| 18019 |  | neomycin sulphate with gramicidin and polymyxin eye drops |
| 41392 |  | neomycin sulphate with gramicidin eye ointment |
| 8353 |  | neomycin sulphate with hydrocortisone eye ointment |
| 12653 |  | neomycin sulphate with hydrocortisone eye ointment |
| 11788 |  | neomycin sulphate with prednisolone 0.5% + 0.5% eye drops |
| 22383 |  | neomycin sulphate with prednisolone 0.5% + 0.5% eye/ear drops |
| 23644 |  | neosporin eye |
| 13468 |  | neosporin eye drops (pliva pharma ltd) |
| 20187 |  | norfloxacin 0.3% eye drops |
| 26586 |  | noroxin 0.30% eye drops (msd thomas morson pharmaceuticals) |
| 3199 |  | ofloxacin 0.3% eye drops |
| 36340 |  | oftaquix 5mg/ml eye drops (santen uk ltd) |
| 68750 |  | oftaquix 5mg/ml eye drops 0.3ml unit dose (santen uk ltd) |
| 40022 |  | oftaquix 5mg/ml eye drops 0.5ml unit dose (santen uk ltd) |
| 32833 |  | okacyn 3mg/ml eye drops (novartis pharmaceuticals uk ltd) |
| 29307 |  | optrex infected eyes 0.5% eye drops (reckitt benckiser healthcare (uk) ltd) |
| 37084 |  | optrex infected eyes 1% eye ointment (reckitt benckiser healthcare (uk) ltd) |
| 20542 |  | opulets chloramphenicol 0.50% eye drops |
| 20055 |  | oxyphenbutazone with chloramphenicol eye ointment |
| 19005 |  | oxytetracycline/polymyxin oint eye |
| 24508 |  | polyfax eye |
| 5686 |  | polyfax ophthalmic ointment (teva uk ltd) |
| 28316 |  | polymixin b 10,000u/bacitracin 500u eye |
| 10833 |  | polymixin b 10,000u/bacitracin 500u eye oin |
| 13778 |  | polymyxin b 10,000units/g / bacitracin 500units/g eye ointment |
| 15973 |  | polymyxin b 10,000units/g / trimethoprim 5mg/g eye ointment |
| 15972 |  | polymyxin b 10,000units/ml / trimethoprim 1mg/ml eye drops |
| 21917 |  | polytrim eye |
| 8309 |  | polytrim eye drops (pliva pharma ltd) |
| 12465 |  | polytrim ophthalmic ointment (pliva pharma ltd) |
| 18602 |  | prednisolone 0.5% / neomycin 0.5% ear/eye drops |
| 11170 |  | prednisolone sodium phosphate 0.5% with neomycin 0.5% eye drops |
| 3936 |  | predsol -n eye drops (ucb pharma ltd) |
| 43088 |  | predsol-n ear/eye drops (focus pharmaceuticals ltd) |
| 10865 |  | sno-phenicol 0.50% eye drops (chauvin pharmaceuticals ltd) |
| 21994 |  | sno-phenicol eye |
| 20660 |  | sofradex ear/eye |
| 39845 |  | sofradex ear/eye drops (sanofi) |
| 7289 |  | sofradex ear/eye drops solution (distriphar (uk)) |
| 34137 |  | sofradex ear/eye drops solution (distriphar (uk)) |
| 4627 |  | sofradex eye drops (distriphar (uk)) |
| 1846 |  | sofradex eye ointment (distriphar (uk)) |
| 6392 |  | soframycin 0.5% eye drops (sanofi) |
| 991 |  | soframycin 0.5% eye ointment (sanofi) |
| 992 |  | soframycin 0.50% eye drops (sanofi) |
| 19534 |  | soframycin eye |
| 19748 |  | soframycin eye |
| 16652 |  | tanderil chloramphenicol eye ointment (novartis consumer health uk ltd) |
| 27843 |  | terramycin + polymyxin b ophthalmic oin |
| 799 |  | tetracycline 1% eye drops |
| 3211 |  | tetracycline 1% eye ointment |
| 21654 |  | tetracycline ear/eye |
| 21629 |  | tetracycline eye |
| 6463 |  | tobradex 3mg/ml / 1mg/ml eye drops (novartis pharmaceuticals uk ltd) |
| 12510 |  | tobralex 0.30% eye drops (alcon laboratories (uk) ltd) |
| 13566 |  | tobramycin 3mg/ml eye drops |
| 25758 |  | tobramycin eye |
| 45746 |  | tobravisc 3mg/ml eye drops (alcon laboratories (uk) ltd) |
| 25074 |  | trimethoprim 0.1%/polymixin b 10000u eye |
| 15062 |  | trimethoprim 0.1%/polymixin b 10000u eye dro |
| 12466 |  | trimethoprim 1mg/polymixin b 10000u eye oin |
| 1743 |  | vista-methasone -n eye drops (martindale pharmaceuticals ltd) |
| 44636 |  | vivioptal junior liquid (scope ophthalmics ltd) |
| 19816 |  | zovirax eye |

## Cutaneous

#### Definitions

| **Outcome** | **Definition** |
| --- | --- |
| Disseminated zoster | Code within 12 months following zoster |
| Cellulitis | Diagnosis codes for the skin condition within 12 month following zoster, without a record of the skin condition in the 4 weeks prior to index date (as this may indicate a recurrent/continuous episode, not related to zoster). |
| Necrotising fasciitis |  |
| Erysipelas |  |

### Disseminated zoster

**Read codes**

| medcode | readterm |
| --- | --- |
| 52319 | Disseminated zoster |

**CPRD Aurum codes**

| medcodeid | term |
| --- | --- |
| 625341000006116 | disseminated zoster |

**ICD-10 codes**

| code | description |
| --- | --- |
| B02.7 | Disseminated zoster |

### Cellulitis

**Read codes**

| medcode | readterm |
| --- | --- |
| 205 | Cellulitis of external ear |
| 309 | Cellulitis and abscess NOS |
| 680 | Cellulitis and abscess of leg NOS |
| 1315 | [X]Cellulitis of other parts of limb |
| 1415 | Cellulitis and abscess of hand |
| 1772 | Cellulitis and abscess of axilla |
| 1874 | Cellulitis and abscess of back |
| 1923 | Cellulitis and abscess of groin |
| 2089 | Cellulitis and abscess of foot unspecified |
| 2216 | Cellulitis and abscess of knee |
| 2658 | Cellulitis and abscess of cheek |
| 2711 | Cellulitis and abscess of neck |
| 2847 | Cellulitis and abscess of thigh |
| 2897 | Cellulitis and abscess of buttock |
| 2914 | Cellulitis and abscess of hand unspecified |
| 3223 | Cellulitis and abscess of elbow |
| 3461 | Cellulitis and abscess of arm |
| 3465 | Cellulitis and abscess of wrist |
| 3597 | Cellulitis and abscess of hip |
| 3998 | Cellulitis and abscess of face |
| 4207 | Cellulitis NOS |
| 4394 | Cellulitis and abscess of chest wall |
| 4400 | Cellulitis and abscess of perineum |
| 4456 | Cellulitis of scrotum |
| 4748 | Oral cellulitis and abscess |
| 4973 | Cellulitis and abscess of abdominal wall |
| 5089 | Cellulitis and abscess of shoulder |
| 5228 | Cellulitis of floor of mouth |
| 6368 | Cellulitis of leg |
| 6833 | Cutaneous cellulitis |
| 7684 | Cellulitis of foot |
| 7821 | Cellulitis of eyelid |
| 7865 | Cellulitis and abscess of leg excluding foot |
| 7972 | Cellulitis of face |
| 8852 | Cellulitis, external ear |
| 9648 | Cellulitis of arm |
| 10326 | Cellulitis and abscess of leg |
| 10485 | Cellulitis and abscess of nose |
| 10974 | Cellulitis and abscess of ankle |
| 12167 | Cellulitis and abscess NOS |
| 14937 | Cellulitis and abscess of umbilicus |
| 14972 | Other specified cellulitis and abscess |
| 15327 | Cellulitis and abscess of submandibular region |
| 15336 | Oral soft tissue cellulitis unspecified |
| 15475 | Cellulitis and abscess of forehead |
| 15549 | Cellulitis and abscess of chin |
| 16011 | Cellulitis of eyelids |
| 16032 | Cellulitis and abscess of temple region |
| 16176 | Cellulitis and abscess of breast |
| 16304 | Cellulitis of penis |
| 16536 | Other cellulitis and abscess |
| 17226 | Cellulitis of axilla |
| 17562 | Cellulitis of lip |
| 19944 | Oral cellulitis and abscess NOS |
| 20389 | Cellulitis and abscess of face NOS |
| 21208 | Cellulitis of dorsum of hand |
| 21580 | Cellulitis and abscess of nose (external) |
| 23585 | Cellulitis and abscess of flank |
| 23604 | Cellulitis and abscess of hand NOS |
| 24401 | Cellulitis and abscess of cheek (external) |
| 24960 | Cellulitis and abscess of head unspecified |
| 25039 | Cellulitis of trunk |
| 25890 | Cellulitis and abscess of lower leg |
| 27619 | Cellulitis of palm of hand |
| 27681 | Cellulitis of face |
| 27717 | Cellulitis and abscess of trunk |
| 27757 | Cellulitis and abscess of foot |
| 27903 | Cellulitis and abscess of forearm |
| 28181 | Cellulitis of neck |
| 29113 | Cellulitis and abscess of foot NOS |
| 29345 | [X]Cellulitis of breast |
| 30260 | [X]Cellulitis of other sites |
| 31534 | Cellulitis of ankle |
| 36349 | Cellulitis and abscess of trunk NOS |
| 44034 | Cellulitis and abscess of upper arm |
| 48630 | Cellulitis and abscess of arm NOS |
| 52366 | Cellulitis of trunk |

**CPRD Aurum codes**

| medcodeid | term |
| --- | --- |
| 25168017 | cellulitis of penis |
| 32771017 | cellulitis of axilla |
| 40768015 | cellulitis of ankle |
| 58130016 | cellulitis of scrotum |
| 62096018 | cellulitis of neck |
| 78133018 | cellulitis of trunk |
| 85030014 | cellulitis of lip |
| 158680013 | cellulitis of external ear |
| 206541013 | cellulitis of foot |
| 302273016 | oral soft tissue cellulitis unspecified |
| 308300010 | cellulitis and abscess of face |
| 308307013 | cellulitis and abscess of chin |
| 308308015 | cellulitis and abscess of submandibular region |
| 308309011 | cellulitis and abscess of forehead |
| 308310018 | cellulitis and abscess of temple region |
| 308311019 | cellulitis of face |
| 308312014 | cellulitis and abscess of face nos |
| 308316012 | cellulitis and abscess of trunk |
| 308317015 | cellulitis and abscess of chest wall |
| 308318013 | cellulitis and abscess of breast |
| 308319017 | cellulitis and abscess of back |
| 308320011 | cellulitis and abscess of abdominal wall |
| 308321010 | cellulitis and abscess of umbilicus |
| 308322015 | cellulitis and abscess of flank |
| 308323013 | cellulitis and abscess of groin |
| 308324019 | cellulitis and abscess of perineum |
| 308325018 | cellulitis and abscess of trunk nos |
| 308326017 | cellulitis and abscess of arm |
| 308327014 | cellulitis and abscess of shoulder |
| 308328016 | cellulitis and abscess of axilla |
| 308329012 | cellulitis and abscess of upper arm |
| 308330019 | cellulitis and abscess of elbow |
| 308331015 | cellulitis and abscess of forearm |
| 308332010 | cellulitis and abscess of arm nos |
| 308342012 | cellulitis and abscess of wrist |
| 308343019 | cellulitis and abscess of hand nos |
| 308344013 | cellulitis and abscess of buttock |
| 308349015 | cellulitis and abscess of hip |
| 308350015 | cellulitis and abscess of thigh |
| 308351016 | cellulitis and abscess of knee |
| 308352011 | cellulitis and abscess of lower leg |
| 308353018 | cellulitis and abscess of ankle |
| 308354012 | cellulitis and abscess of leg nos |
| 308358010 | cellulitis and abscess of foot unspecified |
| 308361011 | cellulitis and abscess of foot nos |
| 308363014 | other specified cellulitis and abscess |
| 308367010 | cellulitis and abscess nos |
| 308368017 | cellulitis nos |
| 308446011 | [x]cellulitis of breast |
| 308453019 | [x]cellulitis of other sites |
| 347348011 | cellulitis of eyelid |
| 357317012 | cellulitis of dorsum of hand |
| 357321017 | cellulitis of palm of hand |
| 357328011 | cellulitis of leg |
| 396325011 | oral cellulitis and abscess nos |
| 399894010 | other cellulitis and abscess |
| 399895011 | cellulitis and abscess of cheek (external) |
| 399896012 | cellulitis and abscess of cheek |
| 399897015 | cellulitis and abscess of nose (external) |
| 399898013 | cellulitis and abscess of neck |
| 399900010 | cellulitis and abscess of hand unspecified |
| 399901014 | cellulitis and abscess of leg |
| 399904018 | cellulitis and abscess of head unspecified |
| 423153016 | cellulitis of arm |
| 472876012 | cutaneous cellulitis |
| 42341000006115 | oral cellulitis and abscess |
| 219471000000116 | cellulitis, external ear |
| 370601000006113 | [x]cellulitis of other parts of limb |
| 540621000006119 | cellulitis and abscess nos |
| 540851000006112 | cellulitis and abscess of foot |
| 540921000006112 | cellulitis and abscess of hand |
| 541011000006113 | cellulitis and abscess of leg excluding foot |
| 541051000006114 | cellulitis and abscess of nose |
| 541281000006113 | cellulitis of eyelids |
| 541291000006111 | cellulitis of face |
| 541421000006113 | cellulitis of trunk |
| 886951000006111 | penile cellulitis/abscess/boil |
| 888981000006116 | cellulitis/abscess nos |
| 888991000006118 | cellulitis/abscess - face |
| 889001000006118 | cellulitis/abscess - neck |
| 889011000006115 | cellulitis/abscess - trunk |
| 889021000006111 | cellulitis/abscess - arm |
| 889041000006116 | cellulitis/abscess - buttock |
| 889051000006119 | cellulitis - leg - excl. foot |
| 906751000006115 | [rfc] cellulitis |

**ICD-10 codes**

| code | description |
| --- | --- |
| H60.1 | Cellulitis of external ear |
| K12.2 | Cellulitis and abscess of mouth |
| L03 | Cellulitis |
| L03.1 | Cellulitis of other parts of limb |
| L03.2 | Cellulitis of face |
| L03.3 | Cellulitis of trunk |
| L03.8 | Cellulitis of other sites |
| L03.9 | Cellulitis, unspecified |

### Necrotising fasciitis

**Read codes**

| medcode | readterm |
| --- | --- |
| 11692 | Necrotising fasciitis |

**CPRD Aurum codes**

| medcodeid | term |
| --- | --- |
| 496327019 | necrotising fasciitis |

**ICD-10 codes**

| code | description |
| --- | --- |
| M72.6 | Necrotizing fasciitis |
| M72.60 | Necrotizing fasciitis |
| M72.61 | Necrotizing fasciitis |
| M72.62 | Necrotizing fasciitis |
| M72.63 | Necrotizing fasciitis |
| M72.64 | Necrotizing fasciitis |
| M72.65 | Necrotizing fasciitis |
| M72.66 | Necrotizing fasciitis |
| M72.67 | Necrotizing fasciitis |
| M72.68 | Necrotizing fasciitis |
| M72.69 | Necrotizing fasciitis |

### Erysipelas

**Read codes**

| medcode | readterm |
| --- | --- |
| 1156 | Erysipelas |
| 27616 | Erysipelas - otitis externa |
| 68242 | Infective otitis externa due to erysipelas |

**CPRD Aurum codes**

| medcodeid | term |
| --- | --- |
| 74483012 | erysipelas |
| 298954015 | infective otitis externa due to erysipelas |
| 298955019 | erysipelas - otitis externa |
| 954061000006115 | notification of erysipelas |

**ICD-10 codes**

| medcodeid | term |
| --- | --- |
| 496327019 | necrotising fasciitis |

## Visceral

#### Definitions

| **Outcome** | **Definition** |
| --- | --- |
| Septicaemia (bacterial or viral) | Codes within 12 months following zoster, with no evidence of visceral condition in 4 weeks prior to zoster (where patients have evidence of the condition in the 4 weeks prior to zoster, any codes following zoster may be due to an episode beginning prior to zoster). |
| Pulmonary embolism/DVT |  |
| Pneumonia (bacterial or viral) |  |
| Acute hepatitis |  |
| Acute pancreatitis |  |
| Acute osteomyelitis |  |
| Acute pleuritis (viral or bacterial) |  |
| Peritonitis |  |
| Myositis |  |
| Myocarditis |  |
| Pericarditis |  |
| Endocarditis |  |

### Septicaemia (bacterial or viral)

**Read codes**

| medcode | readterm | zoster |
| --- | --- | --- |
| 885 | Septicaemia | 0 |
| 1571 | [D]Unspecified viraemia | 0 |
| 1703 | Meningococcal septicaemia | 0 |
| 2136 | Sepsis | 0 |
| 5104 | Gonococcal septicaemia | 0 |
| 7787 | Pneumococcal septicaemia | 0 |
| 10635 | [D]Septic shock | 0 |
| 10872 | Escherichia coli septicaemia | 0 |
| 10978 | Septicaemia due to streptococcus, group B | 0 |
| 12400 | Pseudomonas septicaemia | 0 |
| 12578 | Septicaemia due to enterococcus | 0 |
| 15229 | Streptococcal septicaemia | 0 |
| 16104 | Staphylococcal septicaemia | 0 |
| 18809 | Salmonella septicaemia | 0 |
| 21936 | Meningococcal meningitis with acute meningococcal septicaem | 0 |
| 23079 | [D]Septicaemic shock | 0 |
| 23991 | E.coli septicaemia | 0 |
| 24577 | Meningococcal meningitis with meningococcal septicaemia | 0 |
| 28610 | Haemophilus influenzae septicaemia | 0 |
| 29950 | Septicaemia due to streptococcus, group A | 0 |
| 30102 | Septicaemia due to Staphylococcus aureus | 0 |
| 31517 | Gram negative septicaemia NOS | 0 |
| 31706 | Septicaemia due to anaerobes | 0 |
| 33765 | Septicaemia NOS | 0 |
| 35232 | Septicaemia due to other gram negative organisms | 0 |
| 35451 | Whole blood poisoning | 0 |
| 42825 | Septicaemia due to coagulase-negative staphylococcus | 0 |
| 49590 | Vancomycin resistant enterococcal septicaemia | 0 |
| 52014 | Listeria septicaemia | 0 |
| 53182 | Other specified septicaemias | 0 |
| 53762 | [X]Septicaemia, unspecified | 0 |
| 54077 | Tracheostomy sepsis | 0 |
| 54534 | Serratia septicaemia | 0 |
| 62974 | Bordetella bronchiseptica | 0 |
| 72106 | [X]Other specified septicaemia | 0 |
| 72876 | Other gram negative septicaemia NOS | 0 |
| 72881 | [X]Septicaemia due to other gram-negative organisms | 0 |
| 98545 | [X]Streptococcal septicaemia, unspecified | 0 |
| 99888 | Erysipelothrix septicaemia | 0 |
| 101034 | Biliary sepsis | 0 |
| 101759 | [X]Other streptococcal septicaemia | 0 |
| 104028 | Sepsis | 0 |
| 104141 | Urosepsis | 0 |
| 104150 | Other specified sepsis | 0 |
| 104189 | Sepsis due to Streptococcus group B | 0 |
| 104260 | Sepsis NOS | 0 |
| 104426 | [D]Systemic inflammatory response syndrome [SIRS] | 0 |
| 104474 | Salmonella sepsis | 0 |
| 104492 | Sepsis due to Staphylococcus aureus | 0 |
| 104577 | Sepsis due to Staphylococcus | 0 |
| 104633 | Sepsis due to anaerobes | 0 |
| 104731 | Sepsis due to Streptococcus group A | 0 |
| 104900 | Other streptococcal sepsis | 0 |
| 105053 | Sepsis due to other Gram negative organisms | 0 |
| 105075 | Sepsis due to Gram negative bacteria | 0 |
| 105102 | Sepsis due to anaerobic bacteria | 0 |
| 105198 | Listerial sepsis | 0 |
| 105408 | Sepsis due to Listeria monocytogenes | 0 |
| 105423 | Sepsis due to Streptococcus | 0 |
| 105716 | Streptococcal sepsis, unspecified | 0 |
| 108045 | Sepsis due to Gram negative organisms | 0 |
| 110225 | Sepsis due to staphylococcus NOS | 0 |
| 110263 | Sepsis due to other specified staphylococcus | 0 |
| 112393 | Sepsis due to Haemophilus influenzae | 0 |
| 112542 | Sepsis due to Bacillus anthracis | 0 |
| 112746 | Streptococcal sepsis | 0 |

**CPRD Aurum codes**

| medcodeid | term | zoster |
| --- | --- | --- |
| 18451012 | bordetella bronchiseptica | 0 |
| 41959016 | tracheostomy sepsis | 0 |
| 127157017 | septic shock | 0 |
| 151281010 | sepsis | 0 |
| 286405014 | erysipelothrix septicaemia | 0 |
| 286481019 | septicaemia due to streptococcus, group a | 0 |
| 286484010 | septicaemia due to streptococcus, group b | 0 |
| 286489017 | septicaemia due to streptococcus, group d | 0 |
| 286497012 | septicaemia due to staphylococcus aureus | 0 |
| 286501019 | septicaemia due to coagulase-negative staphylococcus | 0 |
| 286511014 | other specified septicaemias | 0 |
| 288045018 | [x]other streptococcal septicaemia | 0 |
| 288048016 | [x]streptococcal septicaemia, unspecified | 0 |
| 288049012 | [x]septicaemia due to other gram-negative organisms | 0 |
| 288052016 | [x]other specified septicaemia | 0 |
| 288053014 | [x]septicaemia, unspecified | 0 |
| 291664017 | septicaemia | 0 |
| 317357011 | [d]septic shock | 0 |
| 317668018 | [d]unspecified viraemia | 0 |
| 365023014 | staphylococcal septicaemia | 0 |
| 395333017 | septicaemia nos | 0 |
| 454282018 | septicaemia due to enterococcus | 0 |
| 458426011 | meningococcal meningitis with meningococcal septicaemia | 0 |
| 484332018 | streptococcal septicaemia | 0 |
| 492822016 | meningococcal septicaemia | 0 |
| 495504016 | salmonella septicaemia | 0 |
| 495793016 | gonococcal septicaemia | 0 |
| 500396015 | listeria septicaemia | 0 |
| 509175013 | pneumococcal septicaemia | 0 |
| 510257017 | escherichia coli septicaemia | 0 |
| 1206286018 | haemophilus influenzae septicaemia | 0 |
| 1209801015 | urosepsis | 0 |
| 1216098010 | septicaemia due to anaerobes | 0 |
| 1216110018 | [d]septicaemic shock | 0 |
| 1216989010 | serratia septicaemia | 0 |
| 1217028013 | pseudomonas septicaemia | 0 |
| 1232716014 | whole blood poisoning | 0 |
| 2532525012 | vancomycin resistant enterococcal septicaemia | 0 |
| 2840966012 | neutropenic sepsis | 0 |
| 2899947015 | sepsis due to erysipelothrix | 0 |
| 2899948013 | sepsis due to haemophilus influenzae | 0 |
| 2899980010 | sepsis due to gram negative bacteria | 0 |
| 2900059011 | sepsis due to staphylococcus | 0 |
| 2900111014 | sepsis due to bacillus anthracis | 0 |
| 2900218013 | sepsis due to listeria monocytogenes | 0 |
| 2900354014 | sepsis due to staphylococcus aureus | 0 |
| 2900355010 | sepsis due to streptococcus | 0 |
| 2900357019 | sepsis due to streptococcus group b | 0 |
| 2900358012 | sepsis due to streptococcus group d | 0 |
| 2900360014 | sepsis due to streptococcus group a | 0 |
| 2901598017 | sepsis due to anaerobic bacteria | 0 |
| 28961000006118 | other gram negative septicaemia nos | 0 |
| 149081000006113 | septicaemia due to other gram negative organisms | 0 |
| 633541000006114 | e.coli septicaemia | 0 |
| 705871000006118 | meningococcal meningitis with acute meningococcal septicaem | 0 |
| 806571000006110 | gram negative septicaemia nos | 0 |
| 1650161000000119 | biliary sepsis | 0 |
| 1780971000006118 | urosepsis | 0 |
| 1814271000006115 | salmonella sepsis | 0 |
| 1814301000006118 | listerial sepsis | 0 |
| 1814321000006111 | erysipelothrix sepsis | 0 |
| 1814601000006112 | sepsis | 0 |
| 1814621000006119 | streptococcal sepsis | 0 |
| 1814671000006118 | other streptococcal sepsis | 0 |
| 1814681000006115 | streptococcal sepsis, unspecified | 0 |
| 1814711000006119 | sepsis due to other specified staphylococcus | 0 |
| 1814721000006110 | sepsis due to staphylococcus nos | 0 |
| 1814741000006115 | sepsis due to anaerobes | 0 |
| 1814751000006118 | sepsis due to gram negative organisms | 0 |
| 1814781000006114 | sepsis due to other gram negative organisms | 0 |
| 1814791000006112 | other specified sepsis | 0 |
| 1814801000006113 | sepsis nos | 0 |
| 1817031000006115 | [d]systemic inflammatory response syndrome [sirs] | 0 |

**ICD-10 codes**

| code | description | zoster |
| --- | --- | --- |
| A02.1 | Salmonella sepsis | 0 |
| A26.7 | Erysipelothrix sepsis | 0 |
| A32.7 | Listerial sepsis | 0 |
| A40 | Streptococcal sepsis | 0 |
| A40.0 | Sepsis due to streptococcus, group A | 0 |
| A40.1 | Sepsis due to streptococcus, group B | 0 |
| A40.2 | Sepsis due to streptococcus, group D | 0 |
| A40.8 | Other streptococcal sepsis | 0 |
| A40.9 | Streptococcal sepsis, unspecified | 0 |
| A41 | Other sepsis | 0 |
| A41.0 | Sepsis due to Staphylococcus aureus | 0 |
| A41.1 | Sepsis due to other specified staphylococcus | 0 |
| A41.2 | Sepsis due to unspecified staphylococcus | 0 |
| A41.3 | Sepsis due to Haemophilus influenzae | 0 |
| A41.4 | Sepsis due to anaerobes | 0 |
| A41.5 | Sepsis due to other Gram-negative organisms | 0 |
| A41.8 | Other specified sepsis | 0 |
| A41.9 | Sepsis, unspecified | 0 |
| R57.2 | Septic shock | 0 |
| R65 | Systemic Inflammatory Response Syndrome [SIRS] | 0 |
| R65.0 | Systemic Inflammatory Response Syndrome of infectious origin without organ failure | 0 |
| R65.1 | Systemic Inflammatory Response Syndrome of infectious origin with organ failure | 0 |
| R65.9 | Systemic Inflammatory Response Syndrome, unspecified | 0 |

### Pulmonary embolism/DVT

**Read codes**

| medcode | readterm | zoster |
| --- | --- | --- |
| 824 | Deep vein thrombosis | 0 |
| 1266 | Pulmonary embolism | 0 |
| 3392 | DVT - Deep vein thrombosis | 0 |
| 9701 | Pulmonary embolus | 0 |
| 22038 | Deep vein thrombosis of lower limb | 0 |
| 25478 | Deep vein thrombosis, leg | 0 |
| 26650 | Other venous embolism and thrombosis | 0 |
| 34687 | Trendelenburg pulmonary embolectomy | 0 |
| 84367 | Percutaneous transluminal venous thrombolysis NEC | 0 |
| 98526 | Deep vein thrombosis of peroneal vein | 0 |
| 102186 | Percutaneous transluminal venous thrombolysis reconstruction | 0 |
| 104358 | Fogarty embolectomy of vein | 0 |
| 110449 | Provoked deep vein thrombosis | 0 |
| 110542 | Unprovoked deep vein thrombosis | 0 |

**CPRD Aurum codes**

| medcodeid | term | zoster |
| --- | --- | --- |
| 98484016 | pulmonary embolism | 0 |
| 271392011 | trendelenburg pulmonary embolectomy | 0 |
| 300704016 | other venous embolism and thrombosis | 0 |
| 1776763013 | fogarty embolectomy of vein | 0 |
| 2162148012 | dvt - deep vein thrombosis | 0 |
| 2162149016 | deep vein thrombosis | 0 |
| 2162422011 | deep vein thrombosis of lower limb | 0 |
| 193601000006115 | pulmonary embolus | 0 |
| 218551000000118 | deep vein thrombosis, leg | 0 |
| 358041000000114 | percutaneous transluminal venous thrombolysis nec | 0 |
| 884731000006110 | deep venous thrombosis - leg | 0 |
| 884751000006115 | venous thrombosis nos | 0 |
| 905451000006118 | [rfc] pulmonary embolism/pulmonary hypertension | 0 |
| 905491000006112 | [rfc] venous thrombosis | 0 |
| 909471000006114 | [rfc] deep vein thrombosis | 0 |
| 989221000006110 | deep venous thrombosis - leg | 0 |
| 1119161000000115 | deep vein thrombosis of peroneal vein | 0 |
| 1549761000006110 | percutaneous transluminal venous thrombolysis reconstruction | 0 |
| 2488341000000112 | unprovoked deep vein thrombosis | 0 |
| 2488381000000116 | provoked deep vein thrombosis | 0 |

**ICD-10 codes**

| code | description | zoster |
| --- | --- | --- |
| I26 | Pulmonary embolism | 0 |
| I26.0 | Pulmonary embolism with mention of acute cor pulmonale | 0 |
| I26.9 | Pulmonary embolism without mention of acute cor pulmonale | 0 |
| I82 | Other venous embolism and thrombosis | 0 |

**OPCS codes**

| opcscode | opcsterm |
| --- | --- |
| L99.3 | Percutaneous transluminal venous thrombolysis with reconstruction |
| L99.4 | Percutaneous transluminal venous thrombolysis NEC |

### Pneumonia (bacterial or viral)

**Read codes**

| medcode | readterm | zoster |
| --- | --- | --- |
| 68 | Chest infection | 0 |
| 312 | Acute bronchitis | 0 |
| 572 | Pneumonia due to unspecified organism | 0 |
| 886 | Bronchopneumonia due to unspecified organism | 0 |
| 1382 | Acute viral bronchitis unspecified | 0 |
| 1576 | Pneumonia due to mycoplasma pneumoniae | 0 |
| 1849 | Lobar (pneumococcal) pneumonia | 0 |
| 1934 | Laryngotracheobronchitis | 0 |
| 2581 | Chest infection NOS | 0 |
| 3163 | Tracheobronchitis NOS | 0 |
| 3358 | Lower resp tract infection | 0 |
| 3683 | Basal pneumonia due to unspecified organism | 0 |
| 4910 | Interstitial pneumonia | 0 |
| 5202 | Viral pneumonia | 0 |
| 5324 | Atypical pneumonia | 0 |
| 5612 | Pneumonia due to staphylococcus | 0 |
| 5978 | Acute wheezy bronchitis | 0 |
| 6094 | Pneumonia or influenza NOS | 0 |
| 6124 | Acute lower respiratory tract infection | 0 |
| 9043 | Acute pneumococcal bronchitis | 0 |
| 9389 | Chest infection - viral pneumonia | 0 |
| 9639 | Lobar pneumonia due to unspecified organism | 0 |
| 10086 | Pneumonia and influenza | 0 |
| 11072 | Acute purulent bronchitis | 0 |
| 11101 | Acute tracheobronchitis | 0 |
| 11202 | Abscess of lung NOS | 0 |
| 11849 | Other specified pneumonia or influenza | 0 |
| 12423 | Pneumonia due to streptococcus | 0 |
| 13573 | Influenza with bronchopneumonia | 0 |
| 14976 | Viral pneumonia NOS | 0 |
| 15912 | Influenza with pneumonia | 0 |
| 16287 | Chest infection - unspecified bronchopneumonia | 0 |
| 17025 | Chlamydial pneumonia | 0 |
| 17359 | Chest infection - unspecified bronchitis | 0 |
| 19400 | Chest infection - pnemonia due to unspecified organism | 0 |
| 20198 | Acute bronchitis NOS | 0 |
| 21145 | Acute croupous bronchitis | 0 |
| 21185 | Abscess of lung and mediastinum | 0 |
| 21492 | Acute haemophilus influenzae bronchitis | 0 |
| 22795 | Chest infection - other bacterial pneumonia | 0 |
| 22835 | Bronchiolitis obliterans organising pneumonia | 0 |
| 23095 | Bacterial pneumonia NOS | 0 |
| 23333 | Hypostatic pneumonia | 0 |
| 23546 | Pneumonia due to klebsiella pneumoniae | 0 |
| 23726 | Pneumonia with varicella | 1 |
| 24316 | Chest infection with infectious disease EC | 0 |
| 24356 | Hypostatic bronchopneumonia | 0 |
| 24800 | Acute bacterial bronchitis unspecified | 0 |
| 25462 | Varicella pneumonitis | 0 |
| 25694 | Pneumonia due to other specified organisms | 0 |
| 26287 | Klebsiella pneumoniae/cause/disease classifd/oth chapters | 0 |
| 28634 | Other bacterial pneumonia | 0 |
| 29005 | Abscess of lung | 0 |
| 29166 | Chest infection - pneumococcal pneumonia | 0 |
| 29273 | Acute bronchitis due to parainfluenza virus | 0 |
| 29457 | Chest infection - influenza with pneumonia | 0 |
| 29669 | Acute bronchitis and bronchiolitis | 0 |
| 30437 | Pneumonia with whooping cough | 0 |
| 30591 | Pneumonia due to pseudomonas | 0 |
| 30653 | Chest infection - pneumonia organism OS | 0 |
| 31024 | Mycoplasma pneumoniae [PPLO] cause/dis classifd/oth chaptr | 0 |
| 31886 | Acute bronchitis due to mycoplasma pneumoniae | 0 |
| 33478 | Viral pneumonia NEC | 0 |
| 33730 | Single lung abscess | 0 |
| 34251 | Pneumonia due to specified organism NOS | 0 |
| 34659 | Abscess of lung and mediastinum NOS | 0 |
| 35082 | Pneumonia with pertussis | 0 |
| 35189 | Abscess of lung with pneumonia | 0 |
| 35745 | Influenza with pneumonia NOS | 0 |
| 36675 | Pneumonia due to parainfluenza virus | 0 |
| 37447 | Acute lower respiratory tract infection | 0 |
| 37711 | Multiple lung abscess | 0 |
| 37881 | Pneumonia due to haemophilus influenzae | 0 |
| 40498 | Pneumonia with infectious diseases EC | 0 |
| 41137 | Acute bronchitis or bronchiolitis NOS | 0 |
| 43362 | Acute streptococcal bronchitis | 0 |
| 43884 | Pneumonia due to bacteria NOS | 0 |
| 45425 | Pneumonia due to proteus | 0 |
| 48804 | Pneumonia due to haemophilus influenzae | 0 |
| 49398 | Pneumonia with typhoid fever | 0 |
| 49794 | Acute neisseria catarrhalis bronchitis | 0 |
| 50396 | Acute fibrinous bronchitis | 0 |
| 50867 | Pneumonia due to other specified bacteria | 0 |
| 51398 | Pleuropneumonia-like organism (PPLO) infection | 0 |
| 52384 | Pneumonia due to other aerobic gram-negative bacteria | 0 |
| 52520 | [X]Other viral pneumonia | 0 |
| 53753 | [X]Other pneumonia, organism unspecified | 0 |
| 53947 | [X]Pneumonia in viral diseases classified elsewhere | 0 |
| 58896 | Salmonella pneumonia | 0 |
| 60299 | E.coli pneumonia | 0 |
| 62632 | Influenza with pneumonia, influenza virus identified | 0 |
| 63763 | [X]Other bacterial pneumonia | 0 |
| 63858 | Pneumonia due to streptococcus, group B | 0 |
| 65419 | Pneumonia due to escherichia coli | 0 |
| 66362 | Pneumonia with infectious diseases EC NOS | 0 |
| 66397 | [X]Other acute lower respiratory infections | 0 |
| 69782 | Pneumonia with other infectious diseases EC | 0 |
| 70559 | Pneumonia with other infectious diseases EC NOS | 0 |
| 71370 | Acute pseudomembranous bronchitis | 0 |
| 72182 | Pneumonia with salmonellosis | 0 |
| 73100 | [X]Acute bronchitis due to other specified organisms | 0 |
| 73735 | Pneumonia due to pleuropneumonia like organisms | 0 |
| 96059 | Mycoplasma pneumoniae detected | 0 |
| 96583 | [X]Klebsiella pneumoniae/cause/disease classifd/oth chapters | 0 |
| 98381 | [X]Pneumonia due to other specified infectious organisms | 0 |
| 101775 | Acute membranous bronchitis | 0 |
| 103475 | Cryptogenic organising pneumonia | 0 |
| 104121 | Community acquired pneumonia | 0 |
| 104264 | Hospital acquired pneumonia | 0 |
| 106031 | [X]Mycoplasma pneumoniae [PPLO]cause/dis classifd/oth chaptr | 0 |
| 111027 | [X]Pneumonia in bacterial diseases classified elsewhere | 0 |
| 111655 | [X]Pneumonia in other diseases classified elsewhere | 0 |

**CPRD Aurum**

| medcodeid | term | zoster |
| --- | --- | --- |
| 5303018 | salmonella pneumonia | 0 |
| 18268014 | acute bronchitis | 0 |
| 52762016 | hypostatic bronchopneumonia | 0 |
| 56816017 | pneumonia due to streptococcus | 0 |
| 58909014 | acute tracheobronchitis | 0 |
| 69026013 | pneumonia due to pseudomonas | 0 |
| 78272011 | mycoplasma pneumonia | 0 |
| 107173015 | pneumonia due to klebsiella pneumoniae | 0 |
| 107470010 | interstitial pneumonia | 0 |
| 121974010 | abscess of lung | 0 |
| 125510013 | viral pneumonia | 0 |
| 141663013 | hypostatic pneumonia | 0 |
| 142425010 | laryngotracheobronchitis | 0 |
| 301095014 | acute bronchitis and bronchiolitis | 0 |
| 301100011 | acute fibrinous bronchitis | 0 |
| 301103013 | acute purulent bronchitis | 0 |
| 301105018 | acute pneumococcal bronchitis | 0 |
| 301106017 | acute streptococcal bronchitis | 0 |
| 301108016 | acute haemophilus influenzae bronchitis | 0 |
| 301109012 | acute neisseria catarrhalis bronchitis | 0 |
| 301120012 | acute viral bronchitis unspecified | 0 |
| 301121011 | acute bacterial bronchitis unspecified | 0 |
| 301122016 | acute bronchitis nos | 0 |
| 301132011 | acute bronchitis or bronchiolitis nos | 0 |
| 301357010 | pneumonia and influenza | 0 |
| 301363018 | viral pneumonia nec | 0 |
| 301364012 | viral pneumonia nos | 0 |
| 301368010 | other bacterial pneumonia | 0 |
| 301370018 | pneumonia due to other specified bacteria | 0 |
| 301375011 | pneumonia due to bacteria nos | 0 |
| 301376012 | bacterial pneumonia nos | 0 |
| 301377015 | pneumonia due to other specified organisms | 0 |
| 301382010 | pneumonia due to specified organism nos | 0 |
| 301394018 | pneumonia with other infectious diseases ec | 0 |
| 301400016 | varicella pneumonitis | 1 |
| 301403019 | pneumonia with other infectious diseases ec nos | 0 |
| 301404013 | pneumonia with infectious diseases ec nos | 0 |
| 301408011 | pneumonia due to unspecified organism | 0 |
| 301409015 | lobar pneumonia due to unspecified organism | 0 |
| 301413010 | influenza with pneumonia, influenza virus identified | 0 |
| 301414016 | influenza with pneumonia nos | 0 |
| 301430012 | other specified pneumonia or influenza | 0 |
| 301431011 | pneumonia or influenza nos | 0 |
| 301437010 | tracheobronchitis nos | 0 |
| 301680013 | abscess of lung and mediastinum | 0 |
| 301681012 | single lung abscess | 0 |
| 301686019 | abscess of lung with pneumonia | 0 |
| 301687011 | abscess of lung nos | 0 |
| 301688018 | abscess of lung and mediastinum nos | 0 |
| 301809014 | [x]other viral pneumonia | 0 |
| 301810016 | [x]pneumonia due to other aerobic gram-negative bacteria | 0 |
| 301811017 | [x]other bacterial pneumonia | 0 |
| 301812012 | [x]pneumonia due to other specified infectious organisms | 0 |
| 301813019 | [x]pneumonia in bacterial diseases classified elsewhere | 0 |
| 301814013 | [x]pneumonia in viral diseases classified elsewhere | 0 |
| 301817018 | [x]pneumonia in other diseases classified elsewhere | 0 |
| 301818011 | [x]other pneumonia, organism unspecified | 0 |
| 301820014 | [x]acute bronchitis due to other specified organisms | 0 |
| 350041018 | chest infection - unspecified bronchitis | 0 |
| 350044014 | acute bronchitis due to mycoplasma pneumoniae | 0 |
| 350051017 | atypical pneumonia | 0 |
| 350054013 | chlamydial pneumonia | 0 |
| 396090018 | chest infection nos | 0 |
| 396104016 | pneumonia with infectious diseases ec | 0 |
| 396105015 | bronchopneumonia due to unspecified organism | 0 |
| 411490016 | acute wheezy bronchitis | 0 |
| 451130014 | basal pneumonia due to unspecified organism | 0 |
| 474828010 | bronchiolitis obliterans organising pneumonia | 0 |
| 579878017 | acute lower respiratory tract infection | 0 |
| 1222332017 | chest infection - other bacterial pneumonia | 0 |
| 1222333010 | chest infection - pneumonia organism os | 0 |
| 1229740013 | influenza with bronchopneumonia | 0 |
| 1231962012 | pneumonia with pertussis | 0 |
| 1231963019 | pneumonia with whooping cough | 0 |
| 1232627018 | pneumonia due to parainfluenza virus | 0 |
| 1479355018 | community acquired pneumonia | 0 |
| 2164029012 | pneumonia due to mycoplasma pneumoniae | 0 |
| 2475601016 | acute pseudomembranous bronchitis | 0 |
| 2475602011 | acute croupous bronchitis | 0 |
| 2671161016 | pneumonia due to escherichia coli | 0 |
| 2674072012 | hospital acquired pneumonia | 0 |
| 2765492018 | pneumonia due to gram negative bacteria | 0 |
| 219291000006113 | pleuropneumonia-like organism (pplo) infection | 0 |
| 219841000006117 | pneumonia due to haemophilus influenzae | 0 |
| 219851000006115 | pneumonia due to haemophilus influenzae | 0 |
| 219881000006111 | pneumonia due to other aerobic gram-negative bacteria | 0 |
| 219921000006115 | pneumonia due to pleuropneumonia like organisms | 0 |
| 219931000006117 | pneumonia due to proteus | 0 |
| 219971000006119 | pneumonia due to staphylococcus | 0 |
| 219991000006118 | pneumonia due to streptococcus, group b | 0 |
| 220201000006117 | pneumonia with salmonellosis | 0 |
| 220241000006115 | pneumonia with typhoid fever | 0 |
| 220251000006118 | pneumonia with varicella | 1 |
| 394671000006117 | [x]klebsiella pneumoniae/cause/disease classifd/oth chapters | 0 |
| 399241000006111 | [x]mycoplasma pneumoniae [pplo]cause/dis classifd/oth chaptr | 0 |
| 456021000006115 | acute bronchitis due to parainfluenza virus | 0 |
| 457801000006117 | acute lower respiratory tract infection | 0 |
| 546411000006111 | chest infection | 0 |
| 546421000006115 | chest infection - influenza with pneumonia | 0 |
| 546441000006110 | chest infection - pnemonia due to unspecified organism | 0 |
| 546451000006112 | chest infection - pneumococcal pneumonia | 0 |
| 546481000006116 | chest infection - unspecified bronchopneumonia | 0 |
| 546491000006118 | chest infection - viral pneumonia | 0 |
| 546511000006112 | chest infection with infectious disease ec | 0 |
| 633531000006116 | e.coli pneumonia | 0 |
| 683591000006114 | mycoplasma pneumoniae [pplo] cause/dis classifd/oth chaptr | 0 |
| 690181000000115 | mycoplasma pneumoniae detected | 0 |
| 695331000006116 | multiple lung abscess | 0 |
| 733471000006110 | lower resp tract infection | 0 |
| 739941000006111 | lobar (pneumococcal) pneumonia | 0 |
| 754391000006117 | klebsiella pneumoniae/cause/disease classifd/oth chapters | 0 |
| 778871000006116 | influenza with pneumonia | 0 |
| 885041000006119 | acute bronchitis/bronchiolitis | 0 |
| 885191000006112 | lobar -pneumococcal -pneumonia | 0 |
| 885221000006117 | whooping cough pneumonia | 0 |
| 885231000006119 | bronchopneumonia | 0 |
| 885241000006112 | pneumonia nos | 0 |
| 885251000006114 | influenza + pneumonia | 0 |
| 885271000006116 | pneumonia/influenza nos | 0 |
| 907511000006114 | [rfc] chest infection | 0 |
| 932231000006112 | bilateral pneumonia | 0 |
| 1576341000006118 | cause of death- bronchopneumonia | 0 |
| 1760561000006116 | streptococcus pneumoniae | 0 |
| 1772681000006119 | lobar pneumonia | 0 |
| 1772691000006116 | left upper zone pneumonia | 0 |
| 1772701000006116 | lingular pneumonia | 0 |
| 1772711000006118 | basal pneumonia | 0 |
| 1772721000006114 | bilateral basal pneumonia | 0 |
| 1772731000006112 | left lower zone pneumonia | 0 |
| 1772741000006119 | right lower zone pneumonia | 0 |
| 1772751000006117 | right middle zone pneumonia | 0 |
| 1772761000006115 | right upper zone pneumonia | 0 |
| 1772771000006110 | bronchopneumonia | 0 |
| 1772931000006114 | desquamative interstitial pneumonia | 0 |
| 1773251000006116 | nonspecific interstitial pneumonia | 0 |
| 1773261000006119 | fibrotic nonspecific interstitial pneumonia | 0 |
| 1773271000006114 | cellular nonspecific interstitial pneumonia | 0 |
| 1787121000006116 | community acquired pneumonia | 0 |
| 1787131000006118 | hospital acquired pneumonia | 0 |
| 2609031000000111 | influenza with pneumonia due to seasonal influenza virus | 0 |
| 301101010 | acute membranous bronchitis | 0 |
| 301819015 | [x]other acute lower respiratory infections | 0 |
| 474819014 | cryptogenic organising pneumonia | 0 |

**ICD 10 codes**

| code | description | zoster |
| --- | --- | --- |
| B01.2 | Varicella pneumonia | 0 |
| B95.3 | Streptococcus pneumoniae as the cause of diseases classified to other chapters | 0 |
| B96.0 | Mycoplasma pneumoniae [M. pneumoniae] as the cause of diseases classified to other chapters | 0 |
| B96.1 | Klebsiella pneumoniae [K. pneumoniae] as the cause of diseases classified to other chapters | 0 |
| J10.0 | Influenza with pneumonia, seasonal influenza virus identified | 0 |
| J11.0 | Influenza with pneumonia, virus not identified | 0 |
| J12 | Viral pneumonia, not elsewhere classified | 0 |
| J12.0 | Adenoviral pneumonia | 0 |
| J12.2 | Parainfluenza virus pneumonia | 0 |
| J12.8 | Other viral pneumonia | 0 |
| J12.9 | Viral pneumonia, unspecified | 0 |
| J13 | Pneumonia due to Streptococcus pneumoniae | 0 |
| J14 | Pneumonia due to Haemophilus influenzae | 0 |
| J15 | Bacterial pneumonia, not elsewhere classified | 0 |
| J15.0 | Pneumonia due to Klebsiella pneumoniae | 0 |
| J15.1 | Pneumonia due to Pseudomonas | 0 |
| J15.2 | Pneumonia due to staphylococcus | 0 |
| J15.3 | Pneumonia due to streptococcus, group B | 0 |
| J15.4 | Pneumonia due to other streptococci | 0 |
| J15.5 | Pneumonia due to Escherichia coli | 0 |
| J15.6 | Pneumonia due to other Gram-negative bacteria | 0 |
| J15.7 | Pneumonia due to Mycoplasma pneumoniae | 0 |
| J15.8 | Other bacterial pneumonia | 0 |
| J15.9 | Bacterial pneumonia, unspecified | 0 |
| J16 | Pneumonia due to other infectious organisms, not elsewhere classified | 0 |
| J16.0 | Chlamydial pneumonia | 0 |
| J16.8 | Pneumonia due to other specified infectious organisms | 0 |
| J17 | Pneumonia in diseases classified elsewhere | 0 |
| J17.0 | Pneumonia in bacterial diseases classified elsewhere | 0 |
| J17.1 | Pneumonia in viral diseases classified elsewhere | 0 |
| J17.8 | Pneumonia in other diseases classified elsewhere | 0 |
| J18 | Pneumonia, organism unspecified | 0 |
| J18.0 | Bronchopneumonia, unspecified | 0 |
| J18.1 | Lobar pneumonia, unspecified | 0 |
| J18.2 | Hypostatic pneumonia, unspecified | 0 |
| J18.8 | Other pneumonia, organism unspecified | 0 |
| J18.9 | Pneumonia, unspecified | 0 |
| J20 | Acute bronchitis | 0 |
| J20.0 | Acute bronchitis due to Mycoplasma pneumoniae | 0 |
| J20.1 | Acute bronchitis due to Haemophilus influenzae | 0 |
| J20.2 | Acute bronchitis due to streptococcus | 0 |
| J20.4 | Acute bronchitis due to parainfluenza virus | 0 |
| J20.8 | Acute bronchitis due to other specified organisms | 0 |
| J20.9 | Acute bronchitis, unspecified | 0 |
| J22 | Unspecified acute lower respiratory infection | 0 |
| J85 | Abscess of lung and mediastinum | 0 |
| J85.0 | Gangrene and necrosis of lung | 0 |
| J85.1 | Abscess of lung with pneumonia | 0 |
| J85.2 | Abscess of lung without pneumonia | 0 |

### Acute hepatitis

**Read codes**

| medcode | readterm | zoster |
| --- | --- | --- |
| 899 | Hepatitis unspecified | 0 |
| 1440 | Infective hepatitis | 0 |
| 3146 | Viral hepatitis | 0 |
| 5219 | Hepatitis unspecified NOS | 0 |
| 20137 | Unspecified viral hepatitis | 0 |
| 28798 | Nonspecific reactive hepatitis | 0 |
| 30926 | Other specified viral hepatitis without coma | 0 |
| 34358 | Hepatitis in other infectious diseases EC | 0 |
| 41237 | Hepatitis in viral diseases EC | 0 |
| 42222 | Hepatitis in viral diseases EC NOS | 0 |
| 42289 | Hepatitis in infectious diseases EC NOS | 0 |
| 45939 | Sequelae of viral hepatitis | 0 |
| 58876 | Hepatitis in other viral disease | 0 |
| 65335 | Other specified viral hepatitis without mention of coma NOS | 0 |
| 68967 | [X]Viral hepatitis | 0 |
| 69552 | Other specified viral hepatitis with coma | 0 |
| 89587 | Other specified viral hepatitis with hepatic coma NOS | 0 |
| 98805 | [X]Other specified acute viral hepatitis | 0 |
| 101954 | [X]Sequelae of viral hepatitis | 0 |
| 104435 | Acute viral hepatitis NOS | 0 |
| 106017 | Viral hepatitis without hepatic coma | 0 |
| 111975 | Unspecified viral hepatitis with coma | 0 |
| 111976 | Viral hepatitis with hepatic coma | 0 |
| 112028 | [X]Unspecified viral hepatitis with coma | 0 |
| 112029 | [X]Unspecified viral hepatitis without coma | 0 |

**CPRD Aurum**

| medcodeid | term | zoster |
| --- | --- | --- |
| 7302012 | viral hepatitis | 0 |
| 68294019 | viral hepatitis with hepatic coma | 0 |
| 179049010 | viral hepatitis without hepatic coma | 0 |
| 286867013 | other specified viral hepatitis with coma | 0 |
| 286869011 | other specified viral hepatitis with hepatic coma nos | 0 |
| 286870012 | other specified viral hepatitis without coma | 0 |
| 286876018 | unspecified viral hepatitis with coma | 0 |
| 286882015 | unspecified viral hepatitis | 0 |
| 287970018 | sequelae of viral hepatitis | 0 |
| 288168013 | [x]other specified acute viral hepatitis | 0 |
| 288171017 | [x]unspecified viral hepatitis with coma | 0 |
| 288172012 | [x]unspecified viral hepatitis without coma | 0 |
| 303491016 | hepatitis in viral diseases ec | 0 |
| 303497017 | hepatitis in other viral disease | 0 |
| 303499019 | hepatitis in viral diseases ec nos | 0 |
| 303500011 | hepatitis in other infectious diseases ec | 0 |
| 303505018 | hepatitis in infectious diseases ec nos | 0 |
| 303506017 | hepatitis unspecified | 0 |
| 303509012 | hepatitis unspecified nos | 0 |
| 353567019 | nonspecific reactive hepatitis | 0 |
| 1601000006112 | other specified viral hepatitis without mention of coma nos | 0 |
| 222891000000119 | infective hepatitis | 0 |
| 252501000006117 | other viral hepatitis | 0 |
| 426451000006118 | [x]sequelae of viral hepatitis | 0 |
| 432411000006115 | [x]viral hepatitis | 0 |
| 879211000006117 | infectious hepatitis | 0 |
| 879231000006111 | other hepatitis | 0 |
| 906101000006118 | [rfc] hepatitis | 0 |
| 1814911000006116 | acute viral hepatitis nos | 0 |

**ICD 10 codes**

| code | description | zoster |
| --- | --- | --- |
| B17 | Other acute viral hepatitis | 0 |
| B17.8 | Other specified acute viral hepatitis | 0 |
| B17.9 | Acute viral hepatitis, unspecified | 0 |
| B19 | Unspecified viral hepatitis | 0 |
| B19.0 | Unspecified viral hepatitis with hepatic coma | 0 |
| B19.9 | Unspecified viral hepatitis without hepatic coma | 0 |
| B94.2 | Sequelae of viral hepatitis | 0 |
| K75.2 | Nonspecific reactive hepatitis | 0 |

### Acute pancreatitis

**Read codes**

| medcode | readterm | zoster |
| --- | --- | --- |
| 1113 | Acute pancreatitis | 0 |
| 14905 | Acute pancreatitis NOS | 0 |
| 31395 | Acute haemorrhagic pancreatitis | 0 |
| 44571 | Acute suppurative pancreatitis | 0 |
| 49499 | Acute pancreatitis unspecified | 0 |
| 61326 | Pancytopenia with pancreatitis | 0 |
| 104385 | Biliary acute pancreatitis | 0 |
| 105066 | Other acute pancreatitis | 0 |
| 105194 | Idiopathic acute pancreatitis | 0 |

**CPRD Aurum**

| medcodeid | term | zoster |
| --- | --- | --- |
| 303630010 | acute pancreatitis | 0 |
| 303632019 | acute pancreatitis unspecified | 0 |
| 303636016 | acute suppurative pancreatitis | 0 |
| 351123016 | pancytopenia with pancreatitis | 0 |
| 353688019 | idiopathic acute pancreatitis | 0 |
| 396402014 | acute pancreatitis nos | 0 |
| 493743018 | acute haemorrhagic pancreatitis | 0 |
| 1816471000006113 | other acute pancreatitis | 0 |
| 2116751000000111 | biliary acute pancreatitis | 0 |

**ICD 10 codes**

| code | description | zoster |
| --- | --- | --- |
| K85 | Acute pancreatitis | 0 |
| K85.0 | Idiopathic acute pancreatitis | 0 |
| K85.1 | Biliary acute pancreatitis | 0 |
| K85.8 | Other acute pancreatitis | 0 |
| K85.9 | Acute pancreatitis, unspecified | 0 |

### Acute osteomyelitis

**Read codes**

| medcode | readterm | zoster |
| --- | --- | --- |
| 2132 | Unspecified osteomyelitis | 0 |
| 4747 | Unspecified osteomyelitis of the pelvic region and thigh | 0 |
| 5893 | Acute osteomyelitis | 0 |
| 7155 | Bone infection | 0 |
| 10208 | Acute haematogenous osteomyelitis | 0 |
| 14783 | Unspecified osteomyelitis of unspecified site | 0 |
| 15967 | Acute osteomyelitis of other specified site | 0 |
| 16044 | Acute osteomyelitis of the lower leg | 0 |
| 20116 | Hip acute osteomyelitis | 0 |
| 20342 | Osteomyelitis, periostitis, other infections affecting bone | 0 |
| 21800 | Osteomyelitis - jaw | 0 |
| 23599 | Acute osteomyelitis of unspecified site | 0 |
| 24002 | Osteomyelitis of vertebra | 0 |
| 24304 | Unspecified osteomyelitis of the ankle and foot | 0 |
| 24455 | Acute osteomyelitis of the ankle and foot | 0 |
| 25558 | Unspecified osteomyelitis of the lower leg | 0 |
| 34421 | Acute osteomyelitis-tibia | 0 |
| 34626 | Unspecified osteomyelitis NOS | 0 |
| 35090 | Acute osteomyelitis-femur | 0 |
| 37676 | Foot - acute osteomyelitis | 0 |
| 37928 | Bone infection NOS | 0 |
| 37955 | Acute osteomyelitis-phalanx of finger/thumb | 0 |
| 39560 | Ankle - acute osteomyelitis | 0 |
| 39830 | Acute bone infection | 0 |
| 40491 | Acute osteomyelitis-calcaneum | 0 |
| 42160 | Unspecified osteomyelitis of the upper arm | 0 |
| 42905 | Acute osteomyelitis-metatarsal | 0 |
| 42942 | Acute osteomyelitis of the shoulder region | 0 |
| 43058 | Bone infection NOS | 0 |
| 44749 | Acute osteomyelitis-clavicle | 0 |
| 45864 | Acute osteomyelitis of the forearm | 0 |
| 46146 | Pelvis acute osteomyelitis | 0 |
| 46532 | Acute osteomyelitis NOS | 0 |
| 48051 | Unspecified osteomyelitis of other specified site | 0 |
| 48644 | Acute osteomyelitis of the pelvic region and thigh | 0 |
| 50329 | Unspecified osteomyelitis of the hand | 0 |
| 51316 | Unspecified osteomyelitis of the shoulder region | 0 |
| 52775 | Acute osteomyelitis-metacarpal | 0 |
| 53379 | Acute osteomyelitis-fibula | 0 |
| 53854 | Bone infection NOS, of the hand | 0 |
| 54301 | Acute osteomyelitis-phalanx of toe | 0 |
| 54394 | Bone infection NOS, of other specified site | 0 |
| 56046 | Acute osteomyelitis of the upper arm | 0 |
| 56245 | Infection of other tarsal bone | 0 |
| 57140 | Acute osteomyelitis-lumbar spine | 0 |
| 57250 | Acute osteomyelitis-patella | 0 |
| 57619 | Acute osteomyelitis of multiple sites | 0 |
| 57882 | Acute osteomyelitis-ulna | 0 |
| 58066 | Unspecified osteomyelitis of the forearm | 0 |
| 58961 | Other infections involving bone, of the hand | 0 |
| 60400 | Acute osteomyelitis-radius | 0 |
| 61365 | Acute osteomyelitis-thoracic spine | 0 |
| 61612 | Thigh acute osteomyelitis | 0 |
| 62006 | Acute osteomyelitis-talus | 0 |
| 62021 | Acute osteomyelitis-cervical spine | 0 |
| 63841 | Acute osteomyelitis-humerus | 0 |
| 65209 | Other infections involving bone, of the ankle and foot | 0 |
| 67345 | Acute osteomyelitis of the hand | 0 |
| 67357 | Acute osteomyelitis-coccyx | 0 |
| 68844 | Bone infection NOS, of ankle and foot | 0 |
| 69280 | Bone infection NOS, of the lower leg | 0 |
| 69855 | Other infections involving bone, of the lower leg | 0 |
| 70188 | Bone infection NOS, of the upper arm | 0 |
| 71183 | Other infections involving bone, NOS | 0 |
| 71313 | Acute osteomyelitis-pelvis | 0 |
| 71357 | Bone infection NOS, of multiple sites | 0 |
| 71728 | Acute osteomyelitis-sacrum | 0 |
| 72108 | Unspecified osteomyelitis of multiple sites | 0 |
| 72199 | Acute osteomyelitis of jaw | 0 |
| 72873 | [X]Other osteomyelitis | 0 |
| 93793 | Other infections involving bone, of the pelvic region/thigh | 0 |
| 94576 | Bone infection NOS, of the pelvic/thigh | 0 |
| 97366 | Other infections involving bone | 0 |
| 99396 | [X]Other acute osteomyelitis | 0 |
| 100321 | Acute osteomyelitis-other tarsal bone | 0 |
| 111877 | Bone infection NOS, of unspecified site | 0 |

**CPRD Aurum**

| medcodeid | term | zoster |
| --- | --- | --- |
| 54093010 | acute osteomyelitis of jaw | 0 |
| 99455019 | acute osteomyelitis of multiple sites | 0 |
| 311775017 | acute osteomyelitis of unspecified site | 0 |
| 311785016 | acute osteomyelitis of the lower leg | 0 |
| 311790018 | acute osteomyelitis of other specified site | 0 |
| 311792014 | acute osteomyelitis-cervical spine | 0 |
| 311793016 | acute osteomyelitis-thoracic spine | 0 |
| 311794010 | acute osteomyelitis-lumbar spine | 0 |
| 311795011 | acute osteomyelitis-sacrum | 0 |
| 311796012 | acute osteomyelitis-coccyx | 0 |
| 311797015 | acute osteomyelitis-clavicle | 0 |
| 311798013 | acute osteomyelitis-scapula | 0 |
| 311799017 | acute osteomyelitis-humerus | 0 |
| 311800018 | acute osteomyelitis-radius | 0 |
| 311801019 | acute osteomyelitis-ulna | 0 |
| 311802014 | acute osteomyelitis-carpal bone | 0 |
| 311803016 | acute osteomyelitis-metacarpal | 0 |
| 311804010 | acute osteomyelitis-phalanx of finger/thumb | 0 |
| 311805011 | acute osteomyelitis-femur | 0 |
| 311806012 | acute osteomyelitis-patella | 0 |
| 311807015 | acute osteomyelitis-tibia | 0 |
| 311808013 | acute osteomyelitis-fibula | 0 |
| 311809017 | acute osteomyelitis-calcaneum | 0 |
| 311810010 | acute osteomyelitis-talus | 0 |
| 311811014 | acute osteomyelitis-other tarsal bone | 0 |
| 311812019 | acute osteomyelitis-metatarsal | 0 |
| 311813012 | acute osteomyelitis-phalanx of toe | 0 |
| 311815017 | acute haematogenous osteomyelitis | 0 |
| 311816016 | acute osteomyelitis nos | 0 |
| 311855015 | unspecified osteomyelitis of unspecified site | 0 |
| 311856019 | unspecified osteomyelitis of the shoulder region | 0 |
| 311857011 | unspecified osteomyelitis of the upper arm | 0 |
| 311858018 | unspecified osteomyelitis of the forearm | 0 |
| 311859014 | unspecified osteomyelitis of the hand | 0 |
| 311860016 | unspecified osteomyelitis of the pelvic region and thigh | 0 |
| 311861017 | unspecified osteomyelitis of the lower leg | 0 |
| 311862012 | unspecified osteomyelitis of the ankle and foot | 0 |
| 311863019 | unspecified osteomyelitis of other specified site | 0 |
| 311864013 | unspecified osteomyelitis of multiple sites | 0 |
| 311875012 | infection of carpal bone | 0 |
| 311885013 | infection of other tarsal bone | 0 |
| 311888010 | infection of multiple bones | 0 |
| 311889019 | osteomyelitis of vertebra | 0 |
| 311893013 | unspecified osteomyelitis nos | 0 |
| 311951013 | other infections involving bone | 0 |
| 311952018 | other infections involving bone, site unspecified | 0 |
| 311953011 | other infections involving bone, of the shoulder region | 0 |
| 311954017 | other infections involving bone, of the upper arm | 0 |
| 311955016 | other infections involving bone, of the forearm | 0 |
| 311956015 | other infections involving bone, of the hand | 0 |
| 311958019 | other infections involving bone, of the lower leg | 0 |
| 311959010 | other infections involving bone, of the ankle and foot | 0 |
| 311960017 | other infections involving bone, of other specified site | 0 |
| 311961018 | other infections involving bone, of multiple sites | 0 |
| 311962013 | other infections involving bone, nos | 0 |
| 311963015 | bone infection nos | 0 |
| 311964014 | bone infection nos, of unspecified site | 0 |
| 311965010 | bone infection nos, of the shoulder region | 0 |
| 311966011 | bone infection nos, of the upper arm | 0 |
| 311967019 | bone infection nos, of the forearm | 0 |
| 311968012 | bone infection nos, of the hand | 0 |
| 311969016 | bone infection nos, of the pelvic/thigh | 0 |
| 311970015 | bone infection nos, of the lower leg | 0 |
| 311971016 | bone infection nos, of ankle and foot | 0 |
| 311975013 | bone infection nos, of multiple sites | 0 |
| 312695018 | [x]other acute osteomyelitis | 0 |
| 312699012 | [x]other osteomyelitis | 0 |
| 359767013 | foot - acute osteomyelitis | 0 |
| 359768015 | ankle - acute osteomyelitis | 0 |
| 360276014 | bone infection | 0 |
| 400320011 | acute osteomyelitis of the pelvic region and thigh | 0 |
| 400321010 | acute osteomyelitis of the ankle and foot | 0 |
| 400324019 | unspecified osteomyelitis | 0 |
| 400325018 | bone infection nos, of other specified site | 0 |
| 1234478019 | acute osteomyelitis-pelvis | 0 |
| 1494966011 | acute osteomyelitis of the upper arm | 0 |
| 1494968012 | thigh acute osteomyelitis | 0 |
| 1495531012 | acute osteomyelitis of the hand | 0 |
| 2471514017 | acute osteomyelitis | 0 |
| 28391000006113 | other infections involving bone, of the pelvic region/thigh | 0 |
| 40691000006117 | osteomyelitis, periostitis, other infections affecting bone | 0 |
| 220981000000110 | osteomyelitis - jaw | 0 |
| 237641000006118 | pelvis acute osteomyelitis | 0 |
| 455901000006117 | acute bone infection | 0 |
| 458721000006119 | acute osteomyelitis of the forearm | 0 |
| 458761000006113 | acute osteomyelitis of the shoulder region | 0 |
| 520081000006110 | bone infection nos | 0 |
| 823741000006111 | hip acute osteomyelitis | 0 |
| 892051000006113 | acute osteomyelitis - shoulder | 0 |
| 892061000006110 | acute osteomyelitis - elbow | 0 |
| 892071000006115 | acute osteomyelitis - wrist | 0 |
| 892081000006117 | acute osteomyelitis - hand | 0 |
| 892091000006119 | acute osteomyelitis - hip | 0 |
| 892101000006113 | acute osteomyelitis - knee | 0 |
| 892111000006111 | acute osteomyelitis - multiple | 0 |
| 892201000006115 | osteomyelitis nos | 0 |
| 892211000006117 | osteomyelitis nos - shoulder | 0 |
| 892221000006113 | osteomyelitis nos - elbow | 0 |
| 892231000006111 | osteomyelitis nos - wrist | 0 |
| 892241000006118 | osteomyelitis nos - hand | 0 |
| 892251000006116 | osteomyelitis nos - hip | 0 |
| 892261000006119 | osteomyelitis nos - knee | 0 |
| 892271000006114 | osteomyelitis nos - ankle/foot | 0 |
| 892281000006112 | osteomyelitis nos - multiple | 0 |
| 892291000006110 | osteomyelitis nos | 0 |

**ICD 10 codes**

| code | description | zoster |
| --- | --- | --- |
| M46.2 | Osteomyelitis of vertebra | 0 |
| M46.20 | Osteomyelitis of vertebra | 0 |
| M46.21 | Osteomyelitis of vertebra | 0 |
| M46.22 | Osteomyelitis of vertebra | 0 |
| M46.23 | Osteomyelitis of vertebra | 0 |
| M46.24 | Osteomyelitis of vertebra | 0 |
| M46.25 | Osteomyelitis of vertebra | 0 |
| M46.26 | Osteomyelitis of vertebra | 0 |
| M46.27 | Osteomyelitis of vertebra | 0 |
| M46.28 | Osteomyelitis of vertebra | 0 |
| M46.29 | Osteomyelitis of vertebra | 0 |
| M86 | Osteomyelitis | 0 |
| M86.0 | Acute haematogenous osteomyelitis | 0 |
| M86.00 | Acute haematogenous osteomyelitis | 0 |
| M86.01 | Acute haematogenous osteomyelitis | 0 |
| M86.02 | Acute haematogenous osteomyelitis | 0 |
| M86.03 | Acute haematogenous osteomyelitis | 0 |
| M86.04 | Acute haematogenous osteomyelitis | 0 |
| M86.05 | Acute haematogenous osteomyelitis | 0 |
| M86.06 | Acute haematogenous osteomyelitis | 0 |
| M86.07 | Acute haematogenous osteomyelitis | 0 |
| M86.08 | Acute haematogenous osteomyelitis | 0 |
| M86.09 | Acute haematogenous osteomyelitis | 0 |
| M86.1 | Other acute osteomyelitis | 0 |
| M86.10 | Other acute osteomyelitis | 0 |
| M86.11 | Other acute osteomyelitis | 0 |
| M86.12 | Other acute osteomyelitis | 0 |
| M86.13 | Other acute osteomyelitis | 0 |
| M86.14 | Other acute osteomyelitis | 0 |
| M86.15 | Other acute osteomyelitis | 0 |
| M86.16 | Other acute osteomyelitis | 0 |
| M86.17 | Other acute osteomyelitis | 0 |
| M86.18 | Other acute osteomyelitis | 0 |
| M86.19 | Other acute osteomyelitis | 0 |
| M86.8 | Other osteomyelitis | 0 |
| M86.80 | Other osteomyelitis | 0 |
| M86.81 | Other osteomyelitis | 0 |
| M86.82 | Other osteomyelitis | 0 |
| M86.83 | Other osteomyelitis | 0 |
| M86.84 | Other osteomyelitis | 0 |
| M86.85 | Other osteomyelitis | 0 |
| M86.86 | Other osteomyelitis | 0 |
| M86.87 | Other osteomyelitis | 0 |
| M86.88 | Other osteomyelitis | 0 |
| M86.89 | Other osteomyelitis | 0 |
| M86.9 | Osteomyelitis, unspecified | 0 |
| M86.90 | Osteomyelitis, unspecified | 0 |
| M86.91 | Osteomyelitis, unspecified | 0 |
| M86.92 | Osteomyelitis, unspecified | 0 |
| M86.93 | Osteomyelitis, unspecified | 0 |
| M86.94 | Osteomyelitis, unspecified | 0 |
| M86.95 | Osteomyelitis, unspecified | 0 |
| M86.96 | Osteomyelitis, unspecified | 0 |
| M86.97 | Osteomyelitis, unspecified | 0 |
| M86.98 | Osteomyelitis, unspecified | 0 |
| M86.99 | Osteomyelitis, unspecified | 0 |

### Acute pleuritic

**Read codes**

| medcode | readterm | zoster |
| --- | --- | --- |
| 978 | Pleurisy | 0 |
| 19207 | Acute dry pleurisy | 0 |
| 31603 | Staphylococcal pleurisy | 0 |
| 31689 | Bacterial pleurisy with effusion | 0 |
| 32818 | Pneumococcal pleurisy | 0 |
| 37599 | Basal pleurisy | 0 |
| 40819 | Diaphragmatic pleurisy | 0 |
| 43345 | Pneumococcal pleurisy with effusion | 0 |
| 44842 | Bacterial pleurisy with effusion NOS | 0 |
| 47354 | Fibrinous pleurisy | 0 |
| 49452 | Purulent pleurisy | 0 |
| 55211 | Serous pleurisy NOS | 0 |
| 57092 | Encysted pleurisy | 0 |
| 60142 | Exudative pleurisy NOS | 0 |
| 68470 | Serofibrinous pleurisy NOS | 0 |
| 69352 | Streptococcal pleurisy | 0 |
| 93010 | Staphylococcal pleurisy with effusion | 0 |
| 108784 | Streptococcal pleurisy with effusion | 0 |

**CPRD Aurum**

| medcodeid | term | zoster |
| --- | --- | --- |
| 5407016 | pneumococcal pleurisy | 0 |
| 6320012 | staphylococcal pleurisy | 0 |
| 12711015 | streptococcal pleurisy with effusion | 0 |
| 22395010 | diaphragmatic pleurisy | 0 |
| 39802016 | staphylococcal pleurisy with effusion | 0 |
| 40785018 | pneumococcal pleurisy with effusion | 0 |
| 46467017 | encysted pleurisy | 0 |
| 128069014 | fibrinous pleurisy | 0 |
| 141587016 | streptococcal pleurisy | 0 |
| 141595017 | bacterial pleurisy with effusion | 0 |
| 301644013 | pleurisy | 0 |
| 301647018 | acute dry pleurisy | 0 |
| 301650015 | basal pleurisy | 0 |
| 301661011 | bacterial pleurisy with effusion nos | 0 |
| 301669013 | exudative pleurisy nos | 0 |
| 301670014 | serofibrinous pleurisy nos | 0 |
| 301671013 | serous pleurisy nos | 0 |
| 1231843013 | purulent pleurisy | 0 |

**ICD 10 codes**

| code | description | zoster |
| --- | --- | --- |
| R09.1 | Pleurisy | 0 |

### Peritonitis

**Read codes**

| medcode | readterm | zoster |
| --- | --- | --- |
| 948 | [D]Acute abdomen | 0 |
| 2179 | Peritonitis | 0 |
| 5558 | Abscess of suppurative peritonitis | 0 |
| 15656 | O/E - acute abdomen | 0 |
| 15784 | Other suppurative peritonitis NOS | 0 |
| 27298 | Peritonitis NOS | 0 |
| 28895 | Acute peritonitis | 0 |
| 36165 | Other specified peritonitis NOS | 0 |
| 42334 | Other suppurative peritonitis | 0 |
| 42568 | Other specified peritonitis | 0 |
| 54385 | [D]Acute abdomen | 0 |
| 94998 | [X]Other peritonitis | 0 |
| 101301 | Peritonitis in infectious diseases EC NOS | 0 |
| 110756 | Peritonitis in infectious diseases EC | 0 |

**CPRD Aurum**

| medcodeid | term | zoster |
| --- | --- | --- |
| 81079010 | peritonitis | 0 |
| 112270010 | acute peritonitis | 0 |
| 303241017 | peritonitis in infectious diseases ec | 0 |
| 303246010 | peritonitis in infectious diseases ec nos | 0 |
| 303265019 | other suppurative peritonitis nos | 0 |
| 303266018 | other specified peritonitis | 0 |
| 303271013 | other specified peritonitis nos | 0 |
| 303272018 | peritonitis nos | 0 |
| 303776019 | [x]other peritonitis | 0 |
| 317578010 | [d]acute abdomen | 0 |
| 353742019 | abscess of suppurative peritonitis | 0 |
| 396379019 | other suppurative peritonitis | 0 |
| 402481016 | o/e - acute abdomen | 0 |
| 293121000006115 | [d]acute abdomen | 0 |
| 854611000006118 | acute abdomen | 0 |

**ICD 10 codes**

| code | description | zoster |
| --- | --- | --- |
| K65 | Peritonitis | 0 |
| K65.0 | Acute peritonitis | 0 |
| K65.8 | Other peritonitis | 0 |
| K65.9 | Peritonitis, unspecified | 0 |
| R10.0 | Acute abdomen | 0 |

### Myositis

**Read codes**

| medcode | readterm | zoster |
| --- | --- | --- |
| 3649 | Myalgia or myositis NOS | 0 |
| 5492 | Myositis unspecified | 0 |
| 27512 | Myalgia and myositis unspecified | 0 |
| 36866 | Infective myositis | 0 |
| 39740 | Interstitial myositis | 0 |
| 43126 | [X]Other myositis | 0 |
| 67444 | Infective myositis-neck | 0 |
| 93312 | Infective myositis-leg | 0 |
| 94459 | Infective myositis-hand | 0 |
| 100916 | Infective myositis-thigh | 0 |
| 102652 | Infective myositis-back | 0 |
| 112666 | Infective myositis-foot | 0 |

**CPRD Aurum**

| medcodeid | term | zoster |
| --- | --- | --- |
| 49663011 | infective myositis | 0 |
| 93019012 | interstitial myositis | 0 |
| 311572016 | infective myositis-neck | 0 |
| 311573014 | infective myositis-back | 0 |
| 311574015 | infective myositis-shoulder | 0 |
| 311575019 | infective myositis-arm | 0 |
| 311576018 | infective myositis-forearm | 0 |
| 311577010 | infective myositis-hand | 0 |
| 311578017 | infective myositis-pelvis | 0 |
| 311579013 | infective myositis-thigh | 0 |
| 311580011 | infective myositis-leg | 0 |
| 311581010 | infective myositis-foot | 0 |
| 311701010 | myositis unspecified | 0 |
| 311704019 | myalgia or myositis nos | 0 |
| 312633011 | [x]other myositis | 0 |
| 311696015 | myalgia and myositis unspecified | 0 |

**ICD 10 codes**

| code | description | zoster |
| --- | --- | --- |
| M60 | Myositis | 0 |
| M60.0 | Infective myositis | 0 |
| M60.00 | Infective myositis | 0 |
| M60.01 | Infective myositis | 0 |
| M60.02 | Infective myositis | 0 |
| M60.03 | Infective myositis | 0 |
| M60.04 | Infective myositis | 0 |
| M60.05 | Infective myositis | 0 |
| M60.06 | Infective myositis | 0 |
| M60.07 | Infective myositis | 0 |
| M60.08 | Infective myositis | 0 |
| M60.09 | Infective myositis | 0 |
| M60.1 | Interstitial myositis | 0 |
| M60.10 | Interstitial myositis | 0 |
| M60.11 | Interstitial myositis | 0 |
| M60.12 | Interstitial myositis | 0 |
| M60.13 | Interstitial myositis | 0 |
| M60.14 | Interstitial myositis | 0 |
| M60.15 | Interstitial myositis | 0 |
| M60.16 | Interstitial myositis | 0 |
| M60.17 | Interstitial myositis | 0 |
| M60.18 | Interstitial myositis | 0 |
| M60.19 | Interstitial myositis | 0 |
| M60.8 | Other myositis | 0 |
| M60.80 | Other myositis | 0 |
| M60.81 | Other myositis | 0 |
| M60.82 | Other myositis | 0 |
| M60.83 | Other myositis | 0 |
| M60.84 | Other myositis | 0 |
| M60.85 | Other myositis | 0 |
| M60.86 | Other myositis | 0 |
| M60.87 | Other myositis | 0 |
| M60.88 | Other myositis | 0 |
| M60.89 | Other myositis | 0 |
| M60.9 | Myositis, unspecified | 0 |
| M60.90 | Myositis, unspecified | 0 |
| M60.91 | Myositis, unspecified | 0 |
| M60.92 | Myositis, unspecified | 0 |
| M60.93 | Myositis, unspecified | 0 |
| M60.94 | Myositis, unspecified | 0 |
| M60.95 | Myositis, unspecified | 0 |
| M60.96 | Myositis, unspecified | 0 |
| M60.97 | Myositis, unspecified | 0 |
| M60.98 | Myositis, unspecified | 0 |
| M60.99 | Myositis, unspecified | 0 |
| M63.2 | Myositis in other infectious diseases classified elsewhere | 0 |

### Myocarditis

**Read codes**

| medcode | readterm | zoster |
| --- | --- | --- |
| 10415 | Myocarditis NOS | 0 |
| 22639 | Acute myocarditis | 0 |
| 41527 | Acute myocarditis NOS | 0 |
| 61492 | Acute myocarditis, unspecified | 0 |
| 62736 | Acute myocarditis in diseases EC, NOS | 0 |
| 63078 | Other acute myocarditis NOS | 0 |
| 71848 | Septic myocarditis NOS | 0 |
| 72110 | Other acute myocarditis | 0 |
| 72409 | Idiopathic myocarditis NOS | 0 |
| 73153 | [X]Myocarditis in viral diseases classified elsewhere | 0 |
| 98638 | [X]Other acute myocarditis | 0 |
| 110717 | [X]Acute myocarditis, unspecified | 0 |
| 111837 | Acute myocarditis in diseases EC | 0 |

**CPRD Aurum**

| medcodeid | term | zoster |
| --- | --- | --- |
| 77832010 | acute myocarditis | 0 |
| 299928017 | acute myocarditis in diseases ec | 0 |
| 299938010 | acute myocarditis in diseases ec, nos | 0 |
| 299939019 | other acute myocarditis | 0 |
| 299940017 | acute myocarditis, unspecified | 0 |
| 299944014 | idiopathic myocarditis nos | 0 |
| 299948012 | septic myocarditis nos | 0 |
| 299949016 | other acute myocarditis nos | 0 |
| 299950016 | acute myocarditis nos | 0 |
| 300198015 | myocarditis nos | 0 |
| 300904013 | [x]other acute myocarditis | 0 |
| 300905014 | [x]acute myocarditis, unspecified | 0 |
| 300907018 | [x]myocarditis in viral diseases classified elsewhere | 0 |
| 300909015 | [x]myocarditis in other diseases classified elsewhere | 0 |
| 399321000006111 | [x]myocarditis in other infectious+parasitic diseases ce | 0 |
| 905461000006116 | [rfc] endocarditis, myocarditis, pericarditis | 0 |

**ICD 10 codes**

| code | description | zoster |
| --- | --- | --- |
| I40 | Acute myocarditis | 0 |
| I40.0 | Infective myocarditis | 0 |
| I40.8 | Other acute myocarditis | 0 |
| I40.9 | Acute myocarditis, unspecified | 0 |
| I41 | Myocarditis in diseases classified elsewhere | 0 |
| I41.1 | Myocarditis in viral diseases classified elsewhere | 0 |
| I41.2 | Myocarditis in other infectious and parasitic diseases classified elsewhere | 0 |
| I41.8 | Myocarditis in other diseases classified elsewhere | 0 |
| I51.4 | Myocarditis, unspecified | 0 |

### Pericarditis

**Read codes**

| medcode | readterm | zoster |
| --- | --- | --- |
| 3399 | Acute pericarditis | 0 |
| 8411 | Viral pericarditis NOS | 0 |
| 14646 | Other and unspecified acute pericarditis | 0 |
| 15089 | Acute pericarditis in diseases EC NOS | 0 |
| 27606 | Acute pericarditis - unspecified | 0 |
| 29551 | Acute pericarditis in diseases EC | 0 |
| 36755 | Acute pericarditis NOS | 0 |
| 59102 | Acute idiopathic pericarditis | 0 |
| 100907 | [X]Other forms of acute pericarditis | 0 |
| 108258 | [X]Pericarditis in other diseases classified elsewhere | 0 |

**CPRD Aurum**

| medcodeid | term | zoster |
| --- | --- | --- |
| 26378016 | acute pericarditis | 0 |
| 299875018 | acute pericarditis in diseases ec | 0 |
| 299888015 | acute pericarditis in diseases ec nos | 0 |
| 299890019 | other and unspecified acute pericarditis | 0 |
| 299891015 | acute pericarditis - unspecified | 0 |
| 299901010 | acute pericarditis nos | 0 |
| 300887019 | [x]other forms of acute pericarditis | 0 |
| 300891012 | [x]pericarditis in other diseases classified elsewhere | 0 |
| 350428012 | viral pericarditis nos | 0 |
| 395758012 | acute idiopathic pericarditis | 0 |
| 419721000006116 | [x]pericarditis in other infectious+parasitic diseases ce | 0 |
| 905461000006116 | [rfc] endocarditis, myocarditis, pericarditis | 0 |

**ICD 10 codes**

| code | description | zoster |
| --- | --- | --- |
| I30 | Acute pericarditis | 0 |
| I30.0 | Acute nonspecific idiopathic pericarditis | 0 |
| I30.1 | Infective pericarditis | 0 |
| I30.8 | Other forms of acute pericarditis | 0 |
| I30.9 | Acute pericarditis, unspecified | 0 |
| I32 | Pericarditis in diseases classified elsewhere | 0 |
| I32.1 | Pericarditis in other infectious and parasitic diseases classified elsewhere | 0 |
| I32.8 | Pericarditis in other diseases classified elsewhere | 0 |

### Endocarditis

**Read codes**

| medcode | readterm | zoster |
| --- | --- | --- |
| 939 | Endocarditis, valve unspecified, NOS | 0 |
| 12775 | Acute and subacute endocarditis | 0 |
| 27843 | Infective endocarditis in diseases EC, NOS | 0 |
| 31979 | Endocarditis, valve unspecified | 0 |
| 34290 | Acute endocarditis NOS | 0 |
| 38876 | Acute and subacute endocarditis unspecified | 0 |
| 46237 | Endocarditis in disease EC | 0 |
| 48340 | Acute and subacute infective endocarditis in diseases EC | 0 |
| 51472 | Endocarditis, valve unspecified, OS | 0 |
| 66121 | Acute and subacute endocarditis unspecified, NOS | 0 |
| 100924 | Acute myoendocarditis NOS | 0 |
| 110531 | [X]Endocarditis,valve unspecified,in diseases CE | 0 |

**CPRD Aurum**

| medcodeid | term | zoster |
| --- | --- | --- |
| 299902015 | acute and subacute endocarditis | 0 |
| 299912010 | acute and subacute infective endocarditis in diseases ec | 0 |
| 299919018 | infective endocarditis in diseases ec, nos | 0 |
| 299920012 | acute and subacute endocarditis unspecified | 0 |
| 299922016 | acute myoendocarditis nos | 0 |
| 299923014 | acute periendocarditis nos | 0 |
| 299927010 | acute and subacute endocarditis unspecified, nos | 0 |
| 300020018 | endocarditis, valve unspecified | 0 |
| 300024010 | endocarditis, valve unspecified, os | 0 |
| 300025011 | endocarditis in disease ec | 0 |
| 300027015 | endocarditis, valve unspecified, nos | 0 |
| 379761000006111 | [x]endocarditis,valve unspecified,in diseases ce | 0 |
| 884241000006113 | acute/subac. endocarditis nos | 0 |
| 884331000006111 | non-rheum. endocarditis nos | 0 |
| 905461000006116 | [rfc] endocarditis, myocarditis, pericarditis | 0 |
| 990531000006116 | non-rheum. endocarditis nos | 0 |
| 299921011 | acute endocarditis nos | 0 |

**ICD 10 codes**

| code | description | zoster |
| --- | --- | --- |
| I33 | Acute and subacute endocarditis | 0 |
| I33.0 | Acute and subacute infective endocarditis | 0 |
| I33.9 | Acute endocarditis, unspecified | 0 |
| I38 | Endocarditis, valve unspecified | 0 |
| I39 | Endocarditis and heart valve disorders in diseases classified elsewhere | 0 |
| I39.8 | Endocarditis, valve unspecified, in diseases classified elsewhere | 0 |

1. Public Health England. Chapter 28a : Shingles. In: Immunisation against infectious disease. The Green Book. 2016

2. Walker JL, Andrews NJ, Amirthalingam G, et al. Effectiveness of herpes zoster vaccination in an older United Kingdom population. *Vaccine* 2018:36:2371-2377.
